# Comprehensive Mapping of Cyclotides from *Viola philippica* by Using Mass Spectrometry-Based Strategy

**DOI:** 10.3390/molecules29184344

**Published:** 2024-09-13

**Authors:** Liyan Yu, Hailiang Pan, Xiaohang Chen, Shan Gong, Qipeng Zhang, Yandong Zhang, Zhajun Zhan

**Affiliations:** 1 Key Laboratory for Green Pharmaceutical Technologies and Related Equipment of Ministry of Education, College of Pharmaceutical Science, Zhejiang University of Technology, No. 18 Chaowang Road, Gongshu District, Hangzhou 310014, China; yuliyan2160@126.com; 2 Zhejiang Peptides Biotech Co., Ltd., No. 8 Hengyizhi Road, Shengzhou, Shaoxing 312400, China

**Keywords:** *Viola philippica*, cyclotides, cyclic peptides, OEMS

## Abstract

Cyclotides are plant cyclic peptides with exceptional stability and diverse bioactivity, making them promising candidates for biomedical applications. Therefore, the study of cyclotides has attracted increasing attention in recent years. However, the existing cyclotide detection methods face limitations in sensitivity, accuracy, and reliability. To address these challenges, we developed an integrated strategy using a combination of strong cation exchange chromatography techniques for removing interfering small molecules, Orbitrap Exploris 480 mass spectrometry (OEMS); this is a detection and database searching-based method for cyclotide verification, which greatly improved the sensitivity, accuracy, and reliability of cyclotide identification. This strategy was subsequently employed for cyclotide mapping in *Viola* with a minute amount of starting tissue, resulting the identification of 65 known and 18 potentially novel cyclotides, which is the largest dataset of cyclotides for *Viola philippica*. This strategy provided valuable insights into the cyclotide diversity and distribution in *V. philippica*, with potential applications in drug discovery and other biomedical fields.

## 1. Introduction

Cyclotides are unique class of plant peptides characterized by their cyclic backbone and three disulfide bonds, which confer exceptional stability and resistance to proteolytic degradation. These cyclic peptides, typically composed of 28–37 amino acids, have been identified in various plant families, such as *Rubiaceae*, *Cucurbitaceae, Fabaceae*, and *Solanaceae,* and exhibit a wide range of biological activities [1,2,3]. The significance of cyclotides lies in their diverse bioactivities, making them attractive candidates for application in the field of biomedicine. These bioactivities include antimicrobial, anti-HIV, antifouling, hemolytic, immunosuppressive, and cytotoxic activities, suggesting their potential as promising drug leads and therapeutic agents for various diseases and medical conditions [4,5,6,7,8,9,10,11].

Several studies have focused on the identification and characterization of cyclotides in different plant species. In a previous study conducted by He et al., a total of eight new cyclotides (Viphi A-H) and eight known cyclotides were isolated from *Viola philippica* by multiple enzyme digestion and MALDI-TOF MS/MS identification [12]. In another study by Narayani et al., the cyclotides in *Viola odorata* from different locations in India were investigated using liquid chromatography coupled with mass spectrometry [13]. They detected 166 cyclotide-like masses and identified 71 cyclotides using automated spectrum matching. Additionally, three new cyclotides, namely vodo I1, vodo I2, and vodo I3, were sequenced using tandem mass spectrometry. Furthermore, Niyomploy et al. explored the cyclotide diversity in *Rinorea* species from Southeast Asia. They isolated seven cyclotides from *R. virgata* and found a known cyclotide in *R. bengalensis* [14]. Dang et al. focused on the *Viola* species from Vietnam, which had not been previously investigated for cyclotides. They isolated ten cyclotides from *Viola arcuata*, *Viola tonkinensis*, and *Viola austrosinensis*, four of which were novel [15]. Aslam et al. employed a strategy involving reduction, enzymatic digestion, and mass spectrometry sequencing to identify the precursor sequences and cyclotide domains in *V. odorata* leaf tissue [16]. They uncovered 31 cyclotide domains within 122 partial peptide sequences, including 19 putative novel cyclotides and acyclotides. Four precursor sequences were characterized, revealing variations in amino acid residues, while maintaining the classic knotted cyclotide fold.

Despite their immense potential, the existing methods for cyclotide detection suffer from several limitations, encompassing three main aspects. Firstly, in the previous studies, the used sample preparation methods lack a step for the removal of interfering small molecules present in plant extracts. The highly abundant small molecules in plants poses a significant interference in the identification of cyclotides. Secondly, there is a lack of high-accuracy and high-sensitivity cyclotide identification methods at the omics level. The existing techniques, such as MALDI-TOF, have low resolution and cannot accurately analyze the cyclotides in complex samples, while the currently used LC-MS/MS methods also face limitations in sensitivity, taking a large starting sample amount for detection. Thirdly, for the identification of novel cyclotides, several studies used the de novo sequencing approach, but with a high false positive rate, resulting in the low reliability of the obtained novel cyclotides. To address these limitations, we developed a mass spectrometry-based proteomic strategy for the identification of cyclotides in a quite efficient way by using a tiny amount of plant tissue.

## 2. Results and Discussion

### 2.1. Establishment of a Mass Spectrometry-Based Strategy for Cyclotide Identification

**Figure 1:**
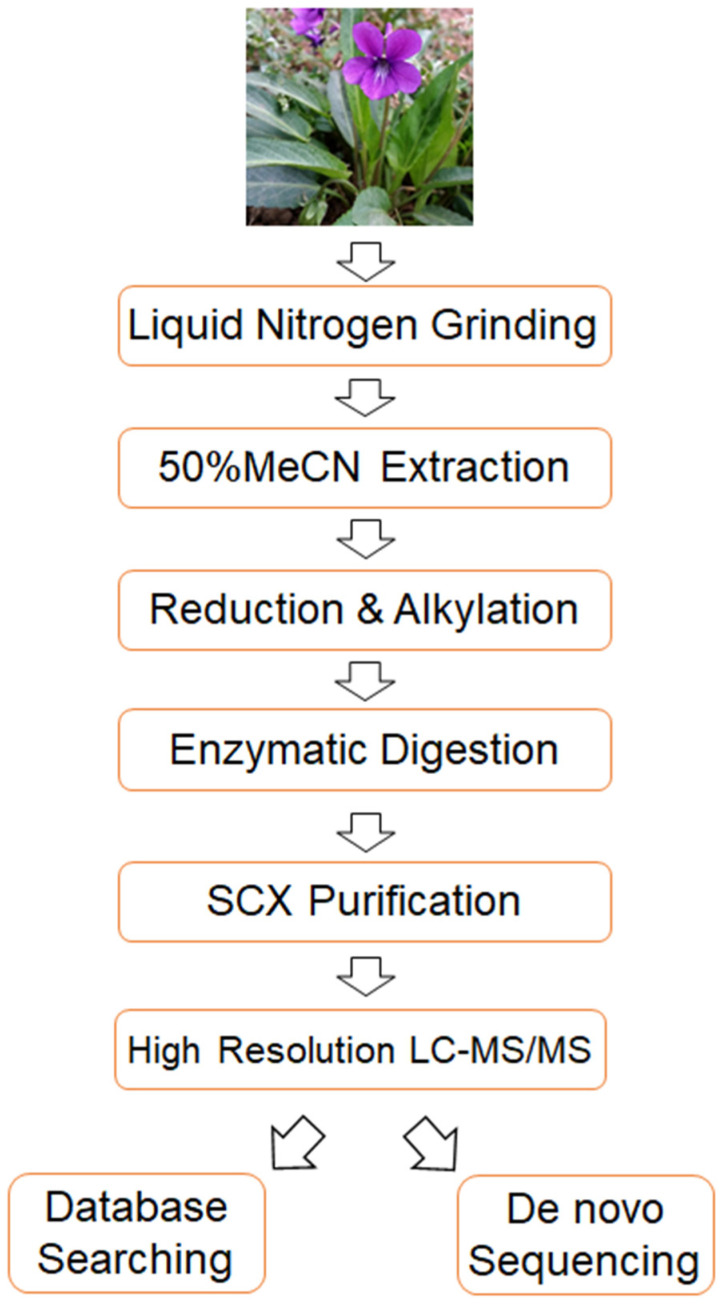
A flow chart of the method developed in this study.

To address the aforementioned challenges in cyclotide detection, we established a highly sensitive and accurate mass spectrometry-based strategy. The flow chart of the strategy is shown in Figure 1. Briefly, it consists of four key steps. First of all, the extracted cyclotides undergo strong cation exchange chromatography (SCX) to remove small molecules, thereby enhancing the sensitivity of cyclotide detection. Secondly, the obtained cyclotides are analyzed using Orbitrap Exploris 480 mass spectrometry, which enhance the sensitivity and accuracy of cyclotide detection. Thirdly, a cyclotide-specific sequence database is constructed, and the acquired mass spectrometry data are analyzed using this database, allowing for the identification of numerous known cyclotides using trypsin alone. Finally, de novo sequencing is employed to identify novel cyclotides from the mass spectrometry data, and a database searching-based method is developed to confirm the results of de novo sequencing, thereby improving the reliability of novel cyclotide identification. Overall, the proposed method enhances the sensitivity, accuracy and reliability of cyclotide identification, enabling the detection of numerous known and potentially novel cyclotides from only a tiny amount of starting material (4 µg of total extract from *Viola philippica* fresh leaves in this study).

### 2.2. The Use of SCX Improves Cyclotide Detection

Plants are important sources of cyclotides, and cyclotides have been detected in varieties of plant families, including *Violaceae*, *Rubiaceae*, *Fabaceae*, *Solanaceae*, and *Cucurbitaceae* [3,11,12,13,14,15,16]. However, the presence of different small molecules in plant tissues, especially in leaves, poses challenges in cyclotide identification. In the conventional strategies, reverse-phase chromatography was usually used for cyclotide separation, as small molecules such as pigments exhibit strong retention on the C18 column, and therefore were very hard to remove using the conventional strategies, leading to significant interference in the following mass spectrometry analysis. Strong cation exchange chromatography (SCX) is a common method used for peptide purification and fractionation in proteomic studies because the stronger binding of peptides allows for effective separation from weakly bound small molecules like pigments [17,18]. Therefore, SCX was introduced to remove small molecules from crude extracts for the purification of cyclotides.

To evaluate the performance of SCX, we compared the differences in the mass spectrometry analysis of cyclotide samples before and after SCX purification. The results showed that when analyzing 1 μg of cyclotide samples without SCX purification, only 18 cyclotides were identified, with only a few cyclotide peaks detected. However, after SCX purification, 46 cyclotides were identified in one LC-MS run, representing a 2.55 times larger improvement (Table S1). Additionally, a significant increase in detected cyclotide peaks was also observed. These results demonstrate that the use of SCX purification enhanced the performance of cyclotide detection in mass spectrometry analysis.

### 2.3. Profiling of Cyclotides from V. philippica Leaves Using Database Searching Strategy

CyBase is a well-known cyclotide database, which contains 747 cyclotides identified from different plant species. To extensively identify these previous known cyclotides from *V. philippica*, we developed a database search method based on a cyclotide-specific sequence library by using CyBase. The methodology and steps were as follows: Firstly, a cyclotide-specific sequence library was constructed from CyBase by in silico digestion based on the enzymatic cleavage characteristics. Taking kalata B1 for example, its original sequence in CyBase and the *V. philippica* database is “GLPVCGETCVGGTCNTPGCTCSWPVCTRN”. After in silico trypsin digestion, the sequence was converted to “NGLPVCGETCVGGTCNTPGCTCSWPVCTR”, where the C-terminal cleavage site of the enzyme was placed at the C-terminus of the sequence, while the remaining amino acids were arranged at the N-terminal. In this example, the terminal amino acid “N” was shifted to the N-terminus, while the trypsin cleavage site “R” remained at the C-terminus. As *V. philippica* does not contain the linear peptide sequence “NGLPVCGETCVGGTCNTPGCTCSWPVCTR”, the detection of this peptide by mass spectrometry confirms its cyclic nature prior to trypsin treatment. This silico trypsin digestion process was applied to all the cyclotides in CyBase to obtain the required sequence database. After completing the sequence library generation, we directly import it into the data retrieval software, Proteome Discoverer 2.4. The mass spectrometry raw data were then searched using standard proteomics data processing methods. This method overcomes the limitations of manual database searching and manual spectrum annotation in the traditional approaches. The data processing software automatically performs database searching and provides matched cyclotide sequences along with annotated tandem mass spectra and fragment ions, facilitating subsequent manual validation.

Combining SCX purification, Orbitrap Exploris 480 mass spectrometry, and the cyclotide-specific database searching method, a total of 65 cyclotides were successfully identified with high confidence using only 4 μg total extract from the *V. philippica* leaves, as presented in Table S2. The manually confirmed MS/MS spectra for these cyclotides are provided in the supplementary data (Supplementary Figures). Though these cyclotides were known previously, not all of them have been found in *V. philippica* before. Therefore, the results improved our knowledge of the diversity of cyclotides in *V. philippica*.

**Figure 2:**
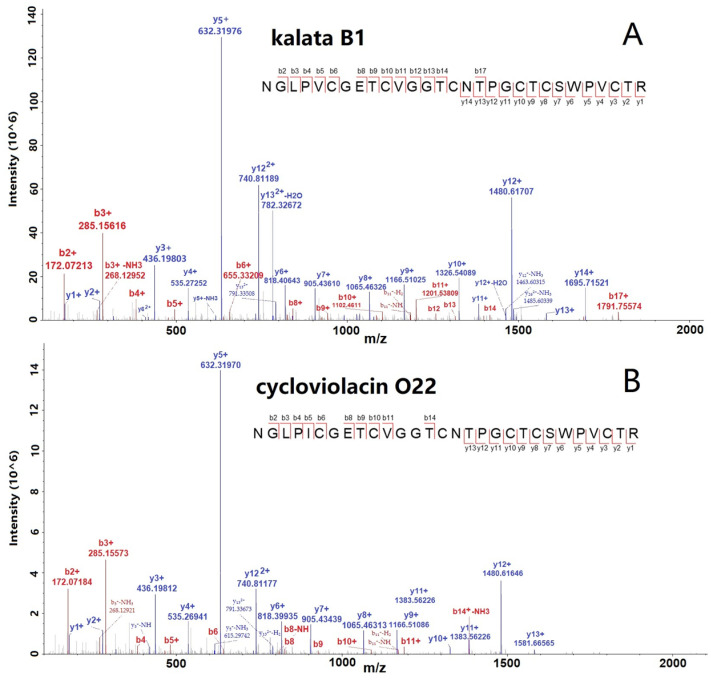
MS/MS spectra of two selected cyclotides, kalata B1 (**A**) and cycloviolacin O22 (**B**), identified from *V. philippica* by using database searching strategy.

The spectra of two selected cyclotides, kalata B1 and Cycloviolacin O22, are shown in Figure 2A,B with many b- and y-ions, indicating the confident identification of cyclotides. Furthermore, we conducted the abundance distribution analysis of the identified 65 cyclotides based on the signals detected in the mass spectrometry data. After sorting, it was observed that cycloviolacin O12 exhibited the highest intensity among the cyclotides, followed by kalata B1, cycloviolacin O22, cycloviolacin T1, and varv peptide D, which ranked from second to fifth, respectively. The intensity ranking of the other cyclotides is illustrated in Table S2.

### 2.4. Identification of Putative Novel Cyclotides from V. philippica Leaves by De Novo Sequencing

The identification of novel cyclotides is of paramount importance, but remains a challenging task. Currently, the most widely used method for the identification of novel cyclotides is the de novo sequencing approach based on mass spectrometry. This method has been extensively employed and has led to the identification of numerous novel cyclotides. But this method usually suffers from a high false-positive rate, which compromises the reliability of the obtained results [19]. To address this issue, we presented a secondary validation approach based on database searching to verify the de novo sequencing results, enhancing the reliability of the newly identified cyclotides.

Initially, the mass spectrometry raw data were processed using MaxQuant software version 2.0.3.0 Subsequently, the identified cyclotide sequences were used to construct a sequence library. Finally, the original mass spectrometry data were subjected to secondary database searching against the sequence library. The cyclotide results with the highest reliability were filtered based on peptide scoring. The detailed experimental procedures are described in the Materials and Methods Section 3. By employing the methodology outlined in this study, we successfully identified 18 new cyclotides using only 4 μg total extract from the *V. philippica* leaves, as shown in Table 1 and Table S3. The new cyclotides were named following the nomenclature scheme proposed by Broussalis et al. using the first letters of the genus and the specific epithet of each species [20].

All the obtained MS/MS spectra were manually validated to checked if there were enough b- or y-ions to unambiguously determine all the amino acid residues. If the sequence was not unambiguously determined, the amino acid residues were underlined. Because b1 and b2 ions are always missing in MS experiments, the sequence of the first two or three residues usually cannot be unambiguously determined. Moreover, as de novo sequencing cannot distinguish leucine from isoleucine, all the I/L residues in the putative novel cyclotide sequence were marked as “X”, as shown in a previous study [13].

**Figure 3:**
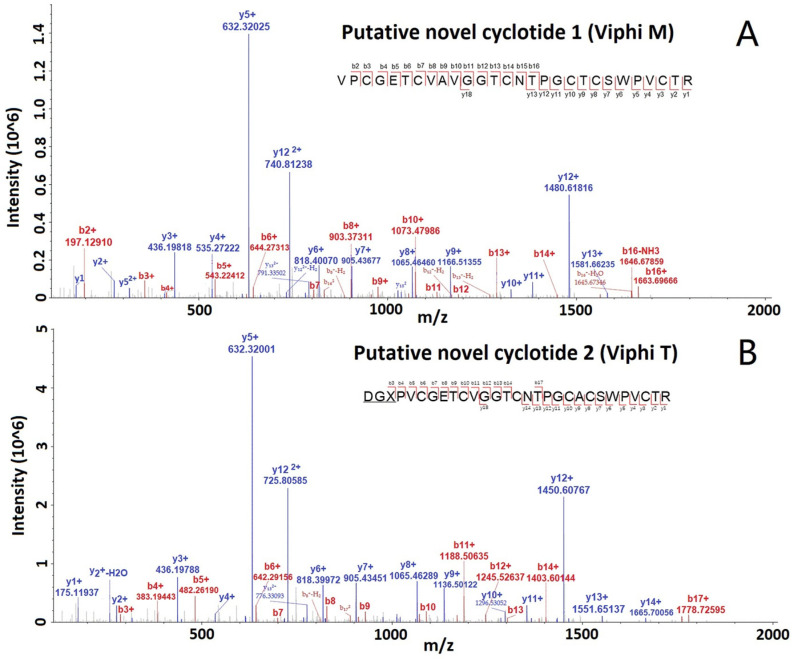
MS/MS spectra of two selected cyclotides, Viphi M (**A**) and Viphi T (**B**), identified from *V. philippica* by using de novo sequencing and database searching-based validation.

All the MS/MS spectra for these cyclotides are provided in the Supplementary Figures. The spectra of two selected putative novel cyclotides, Viphi M and Viphi T, are shown in Figure 3, with a lot of b- and y-ions, and the matched ions are consecutive, indicating the accurate identification of the two cyclotides.

**Figure 4:**
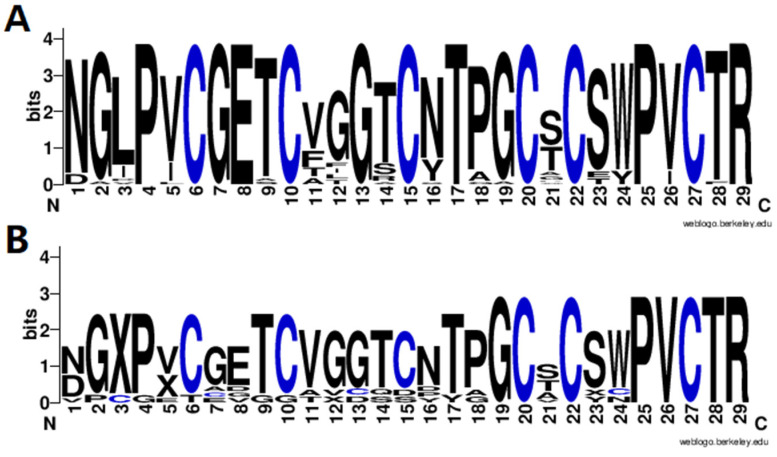
Sequence motif analysis of the identified known (**A**) and novel (**B**) cyclotides.

Finally, the amino acid sequence distribution of the identified cyclotides was analyzed using WebLogo3 software [21] to show the diversity of cyclotides in *V. philippica,* and the results are shown in Figure 4. It can be observed that the cyclotides exhibit a highly conserved distribution of amino acids, including six cysteine residues and amino acids in the other positions. It is obvious that the motif of putative novel cyclotides (Figure 4B) was very similar with that of the known cyclotides from CyBase (Figure 4A), suggesting the reliable identification of novel cyclotides. To further confirm these cyclotides, more validation experiments are needed, including in vitro synthesis and activity testing.

## 3. Materials and Methods

### 3.1. Cyclotide Extraction from V. philippica Leaves

Samples of *V. philippica* were collect from Beidou Town, Renshou County, Meishan City, Sichuan Province, China. The plant material was identified by the authors and was deposited at the College of Pharmaceutical Science, Zhejiang University of Technology. Two biological replicates and two technical replicates for each biological replicate were prepared.

For cyclotide extraction, 50 mg of *V. philippica* leaves was placed in pre-cooled mortar with liquid nitrogen and ground to a fine powder. Then, the sample was mixed with a four-times-larger volume of lysis buffer (containing 50% acetonitrile and 1% formic acid) and subjected to ultrasonication for lysis [14]. After centrifugation at 4 °C using 12,000× *g* for 10 min, the supernatant was transferred to a new centrifuge tube, and the concentration was determined using a BCA protein assay kit.

### 3.2. Reduction, Alkylation, and Enzymatic Digestion

One hundred µg of total extract was mixed with dithiothreitol (DTT) to achieve a final concentration of 10 mM and reduced at 37 °C for 60 min. Then, iodoacetamide was added to achieve a final concentration of 20 mM and incubated at room temperature in the dark for 45 min. Trypsin was added in a protein-to-enzyme ratio of 50:1, and enzymatic digestion was carried out for 12 h. After digestion, the peptide mixture was acidified with trifluoroacetic acid (TFA) to pH 2–3, followed by centrifugation at room temperature at 12,000× *g* for 10 min. The supernatant was transferred to a new centrifuge tube and subjected to desalting using a reverse-phase solid-phase extraction (SPE) column. The desalted peptides were then vacuum-dried and stored for further analysis.

### 3.3. Strong Cation Exchange Chromatography Purification

The concentrated cyclotide sample was reconstituted in 0.5% acetic acid aqueous solution and loaded onto a strong cation exchange SPE column (Guangzhou FLM Scientific, Guangzhou, China, 9B-T011-01100). The SPE column was first washed twice with 0.5% acetic acid aqueous solution, followed by two washes with 0.5% acetic acid/80% acetonitrile solution. Finally, the peptides were eluted from the SPE column with 0.1% ammonia/20% acetonitrile solution, and the eluates were collected, vacuum-dried, and stored for subsequent analysis.

### 3.4. High-Resolution Liquid Chromatography–Mass Spectrometry Detection

The cyclotide sample prepared from 100 µg total extract was dissolved by adding 400 µL mobile phase A (0.1% formic acid and 5% acetonitrile in water) and separated using an EASY-nLC 1200 ultra-high-performance liquid chromatography system (Thermo Scientific, Bremen, Germany) with a home-made analytical column that was 25 cm long and had a 100 μm inner diameter; it was packed in-house with ReproSil-Pur C18-AQ 1.9 μm resin (ReproSil-Pur^®^, Dr. Maisch GmbH, Ammerbuch, Germany). In each LC-MS/MS run, 4 µL cyclotide sample was first loaded onto the analytical column, and then analyzed with the gradient elution method. The gradient was set as follows, 0–38 min, 8%~30% B; 38–52 min, 30%~50% B; 52–56 min, 50%~80% B; 56–60 min, 80% B (0.1% formic acid and 90% acetonitrile in water), with a constant flow rate of 500 nL/min. The peptides were ionized in an NSI (nano-electrospray ionization) ion source, and then the ions were subjected to analysis using an Orbitrap Exploris 480 mass spectrometer (Thermo Scientific, Bremen, Germany). The ion source voltage was set at 2.3 kV. Both the precursor ions and their fragment ions were detected and analyzed using high-resolution Orbitrap. The first MS scan range was set from 400 to 1600 *m*/*z*, with a resolution of 60,000, and the starting point of the second MS scan was fixed at 110 *m*/*z*, and the resolution was set at 15,000. Data acquisition was performed in data-dependent acquisition (DDA) mode, where the 25 most intense precursor ions were selected for higher-energy collisional dissociation (HCD) fragmentation with 27% collision energy and subsequent MS/MS analysis. To improve the efficiency of data acquisition, automatic gain control (AGC) was set at 100%, and the signal threshold was set at 50,000 ions/s. The maximum injection time was set automatically, and a dynamic exclusion time of 20 s was applied to avoid the repeated scanning of precursor ions.

### 3.5. Database Construction and Spectral Data Interpretation

All the cyclotide sequences from CyBase [22] (http://cybase.org.au/, accessed on 6 October 2023) were subjected to theoretical trypsin digestion, and new peptide sequences were generated to build a cyclotide-specific sequence database. Spectral data interpretation was performed using Proteome Discoverer 2.4. The database search parameters were set as follows: the cyclotide-specific amino acid sequence was used as the database, Trypsin/P was selected as the enzyme, the number of missed cleavage sites was set to 2, the mass tolerance for precursor ions was set at 10 ppm, and the mass tolerance for fragment ions was set at 0.02 Da. The carbamidomethylation of cysteine was set as a fixed modification, and the oxidation of methionine was set as a variable modification. Peptide ion scores greater than 20 and peptide confidence levels of “High” were required for identification, with a false discovery rate (FDR) controlled within 1%.

### 3.6. De Novo Sequencing of Mass Spectrometry Data

The de novo sequencing of the mass spectrometry data was performed using MaxQuant v2.1.0.0 [23]. The search parameters were set as follows: the de novo sequencing module of the software was selected, Trypsin/P was chosen as the enzyme, the mass tolerance for precursor ions was set at 10 ppm, and the mass tolerance for fragment ions was set at 0.02 Da. The carbamidomethylation of cysteine was set as a fixed modification, and the oxidation of methionine was set as a variable modification.

For the verification of the results, the identified cyclotides from de novo sequencing were assembled into a new peptide sequence database and imported into Proteome Discoverer 2.4 [24] for re-analysis. The search parameters were set as described above for database search. All the obtained MS/MS spectra were manually validated to checked if there were enough b- or y-ions to unambiguously determine all the amino acid residues. If the sequence was not unambiguously determined, the amino acid residues were underlined. Moreover, as de novo sequencing cannot distinguish leucine from isoleucine, all the I/L residues in the putative novel cyclotide sequence were marked as “X”.

## 4. Conclusions

In this study, we developed a mass spectrometry-based strategy for the identification of cyclotides in *V. philippica*. Through the utilization of strong cation exchange chromatography, automated spectrum matching, and de novo peptide sequencing, we successfully identified 65 previously known cyclotides and discovered 18 putative novel cyclotides. This extensive characterization provides valuable insights into the cyclotide diversity and distribution in *V. philippica.*

The application of mass spectrometry in cyclotide research has revolutionized the field by enabling rapid and comprehensive analysis. Our study demonstrates the power of high-resolution mass spectrometry-based strategies in cyclotide detection and identification. Furthermore, the discovery of 18 putative novel cyclotides expands the cyclotide library. These findings highlight the potential of mass spectrometry-based approaches in cyclotide research.

## Figures and Tables

**Table 1:** Putative novel cyclotides identified from *V. philippica*.

Name	Cyclotide Sequence	Length
Viphi I	VPCGDPSPTCVNTCNTPGCSCSWPVCTR	28
Viphi J	XGPVCADTCTXGTCYTAGCSCSWPVCTR	28
Viphi K	XGPVCGETCTXGTCYTAGCSCSWPVCTR	28
Viphi L	NGXPVCGETCVCYSSDPGCTCSWPVCTR	28
Viphi M	VPCGETCVAVGGTCNTPGCTCSWPVCTR	28
Viphi N	DGXPXCGETCVGGTCNTPGCSCSWPVCTR	29
Viphi O	DGXPVCGETCVGGTCNTPGCSCSWPVCTR	29
Viphi P	NGXPXCGETCVGGTCNTPGCVCSWPVCTR	29
Viphi Q	DGXPVCGETCTXGTCYTAGCSCSWPVCTR	29
Viphi R	NGXPXCGETCVGGTCDTPGCTCSWPVCTR	29
Viphi S	NGXPXCGETCVGDSDPTPGCTCXCPVCTR	29
Viphi T	DGXPVCGETCVGGTCNTPGCACSWPVCTR	29
Viphi U	NGXPVCEGTCVGGTCNYGGCSCSWPVCTR	29
Viphi V	VPCGETCVGGAVCQSNTPGCTCSWPVCTR	29
Viphi W	NGXPVCADTCVGGTCNTPGCACYNPVCTR	29
Viphi X	NGXPXCADTCVGGTCNTPGCSCSMAPVCTR	30
Viphi Y	VCYNGXTMCSSCVWXPCTVTAXVGCSCSDK	30
Viphi Z	NGXPXCEGTCVGGTCNTPGCSCSMAPVCTR	30

## Data Availability

Data are contained within the article and Supplementary Materials.

## Supplementary Materials

The following supporting information can be downloaded at: https://www.mdpi.com/article/10.3390/molecules29184344/s1, Table S1: The cyclotides identified from *V. philippica* before and after SCX purification; Table S2: The cyclotides identified from *V. philippica* by the database searching strategy; Table S3: The putative novel cyclotides identified from *V. philippica* by de novo sequencing; Supplementary Figures: MS2 spectrum of known cyclotides.

## References

[1] Craik D.J., Daly N.L., Bond T., Waine C. (1999). Plant cyclotides: A unique family of cyclic and knotted proteins that defines the cyclic cystine knot structural motif. J. Mol. Biol..

[2] Gould A., Camarero J.A. (2017). Cyclotides: Overview and biotechnological applications. ChemBioChem.

[3] de Veer S.J., Kan M.W., Craik D.J. (2019). Cyclotides: From structure to function. Chem. Rev..

[4] Pränting M., Lööv C., Burman R., Göransson U., Andersson D.I. (2010). The cyclotide cycloviolacin O_2_ from *Viola odorata* has potent bactericidal activity against Gram-negative bacteria. J. Antimicrob. Chemother..

[5] Wang C., Colgrave M.L., Gustafson K.R., Ireland D.C., Goransson U., Craik D.J. (2008). Anti-HIV cyclotides from the Chinese medicinal herb *Viola yedoensis*. J. Nat. Prod..

[6] Bokesch H.R., Pannell L.K., Cochran P.K., Sowder R.C., McKee T.C., Boyd M.R. (2001). A novel anti-HIV macrocyclic peptide from *Palicourea condensata*. J. Nat. Prod..

[7] Gustafson K.R., McKee T.C., Bokesch H.R. (2004). Anti-HIV cyclotides. Curr. Protein Pept. Sci..

[8] Göransson U., Sjögren M., Svangård E., Claeson P., Bohlin L. (2004). Reversible antifouling effect of the cyclotide cycloviolacin O_2_ against barnacles. J. Nat. Prod..

[9] Chen B., Colgrave M.L., Wang C., Craik D.J. (2006). Cycloviolacin H_4_, a hydrophobic cyclotide from *Viola hederaceae*. J. Nat. Prod..

[10] Gründemann C., Koehbach J., Huber R., Gruber C.W. (2012). Do plant cyclotides have potential as immunosuppressant peptides?. J. Nat. Prod..

[11] Svangård E., Göransson U., Hocaoglu Z., Gullbo J., Larsson R., Claeson P., Bohlin L. (2004). Cytotoxic cyclotides from *Viola tricolor*. J. Nat. Prod..

[12] He W., Chan L.Y., Zeng G., Daly N.L., Craik D.J., Tan N. (2011). Isolation and characterization of cytotoxic cyclotides from *Viola philippica*. Peptides.

[13] Narayani M., Chadha A., Srivastava S. (2017). Cyclotides from the Indian medicinal plant *Viola odorata* (Banafsha): Identification and characterization. J. Nat. Prod..

[14] Niyomploy P., Chan L.Y., Harvey P.J., Poth A.G., Colgrave M.L., Craik D.J. (2018). Discovery and characterization of cyclotides from *Rinorea* species. J. Nat. Prod..

[15] Dang T., Chan L.Y., Huang Y.-H., Nguyen L.T.T., Kaas Q., Huynh T., Craik D.J. (2020). Exploring the sequence diversity of cyclotides from *Vietnamese viola* species. J. Nat. Prod..

[16] Aslam L., Kaur R., Sharma V., Kapoor N., Mahajan R. (2021). Isolation and characterization of cyclotides from the leaves of *Viola odorata* L. using peptidomic and bioinformatic approach. 3 Biotech.

[17] Rappsilber J., Mann M., Ishihama Y. (2007). Protocol for micro-purification, enrichment, pre-fractionation and storage of peptides for proteomics using StageTips. Nat. Protoc..

[18] Washburn M.P., Wolters D., Yates J.R. (2001). Large-scale analysis of the yeast proteome by multidimensional protein identification technology. Nat. Biotechnol..

[19] Blank-Landeshammer B., Kollipara L., Biß K., Pfenninger M., Malchow S., Shuvaev K., Zahedi R.P., Sickmann A. (2017). Combining de novo peptide sequencing algorithms, a synergistic approach to boost both identifications and confidence in bottom-up proteomics. J. Proteome Res..

[20] Broussalis A.M., Göransson U., Coussio J.D., Ferraro G., Martino V., Claeson P. (2001). First cyclotide from *Hybanthus* (Violaceae). Phytochemistry.

[21] Crooks G.E., Hon G., Chandonia J.M., Brenner S.E. (2004). WebLogo: A sequence logo generator. Genome Res..

[22] Wang C., Kaas Q., Chiche L., Craik D.J. (2008). CyBase: A database of cyclic protein sequences and structures, with applications in protein discovery and engineering. Nucleic Acids Res..

[23] Tyanova S., Temu T., Cox J. (2016). The MaxQuant computational platform for mass spectrometry-based shotgun proteomics. Nat. Protoc..

[24] Orsburn B.C. (2021). Proteome Discoverer-A community enhanced data processing suite for protein informatics. Proteomes.

