# Supplementary material for: Comprehensive Mapping of Cyclotides from Viola philippica by Using Mass Spectrometry-Based Strategy
Source: Molecules. 2024 Sep 13;29(18):4344. doi: 10.3390/molecules29184344 (PMC11434059; doi:10.3390/molecules29184344)

MS2 spectrum of known cyclotides

alca_2_NGIPCGESCVFIPCISGVLGCSCSNK

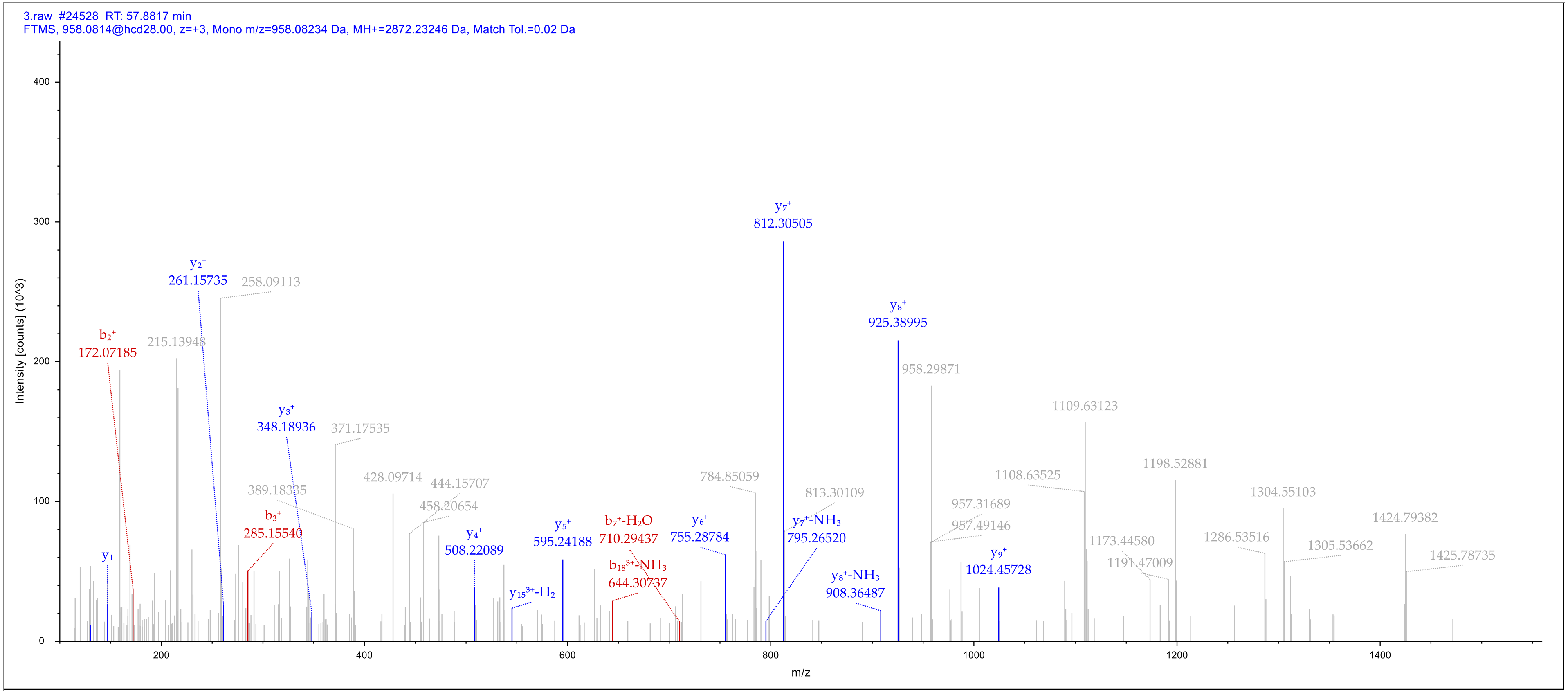

cycloviolacin_H3_NGLPVCGETCFGGTCNTPGCICDPWPVCTR

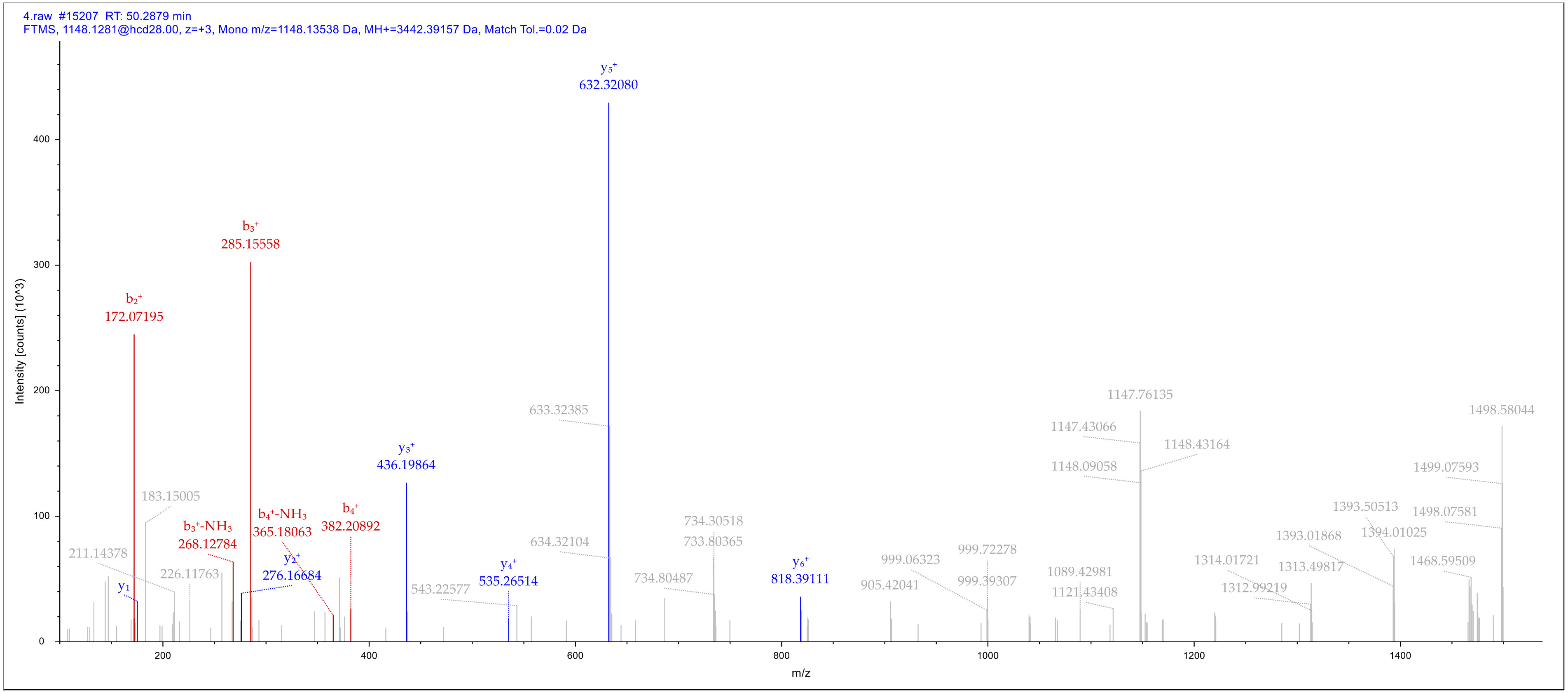

cycloviolacin_O12_NGLPICGETCVGGTCNTPGCSCSWPVCTR

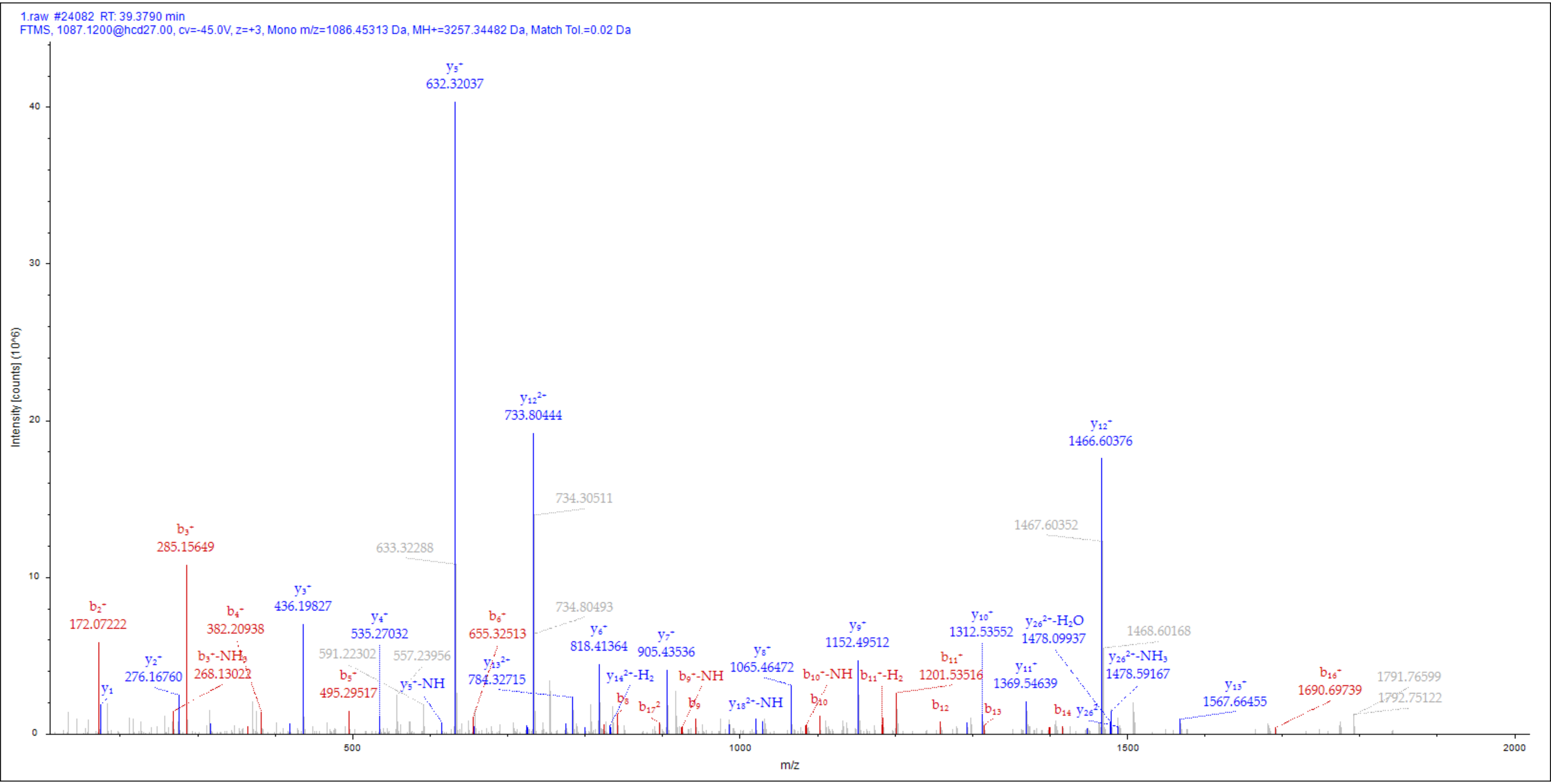

cycloviolacin_O2_NGIPCGESCVWIPCISSAIGCSCK

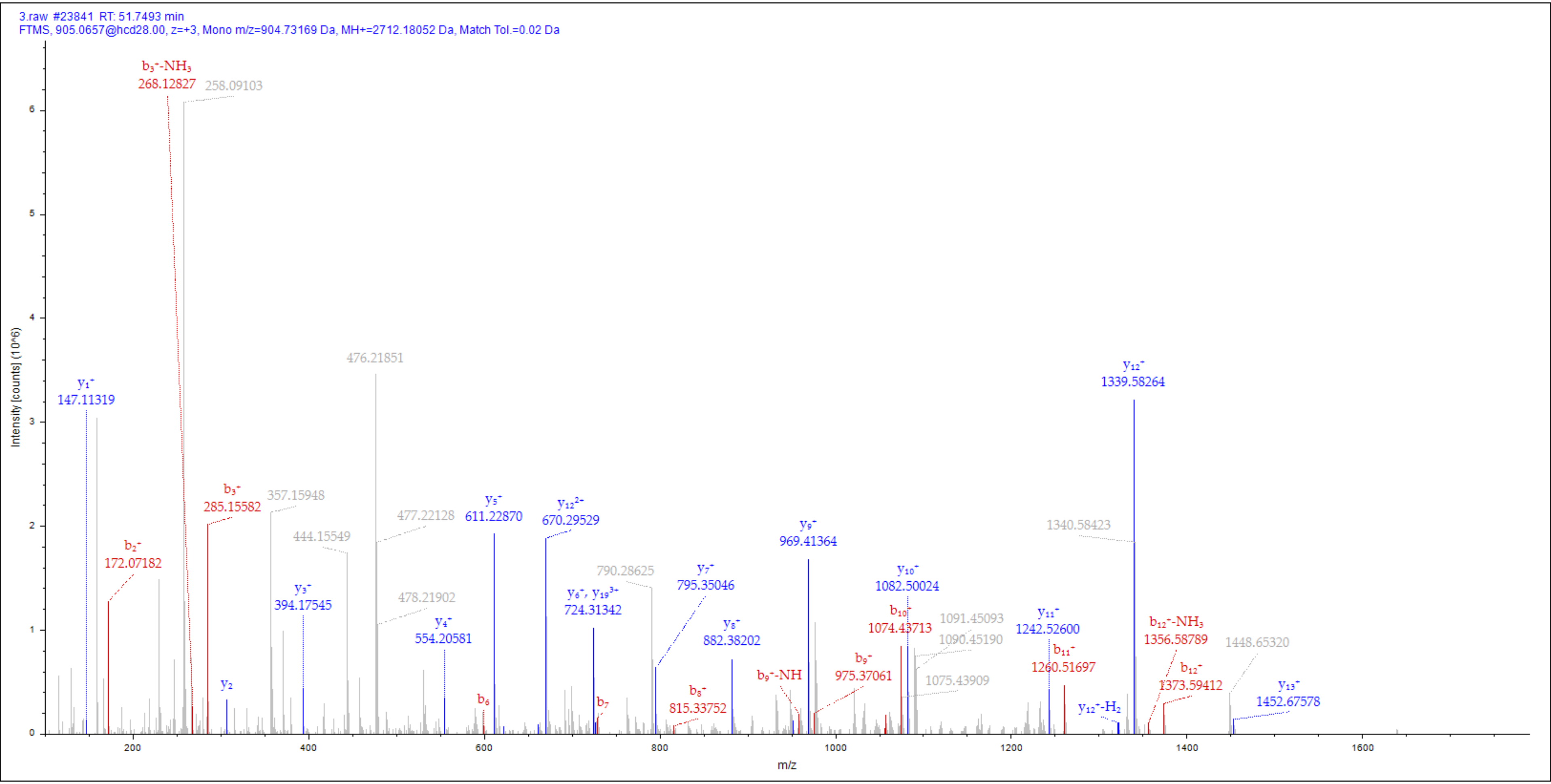

cycloviolacin_O21_NGLPVCGETCVTGSCYTPGCTCSWPVCTR

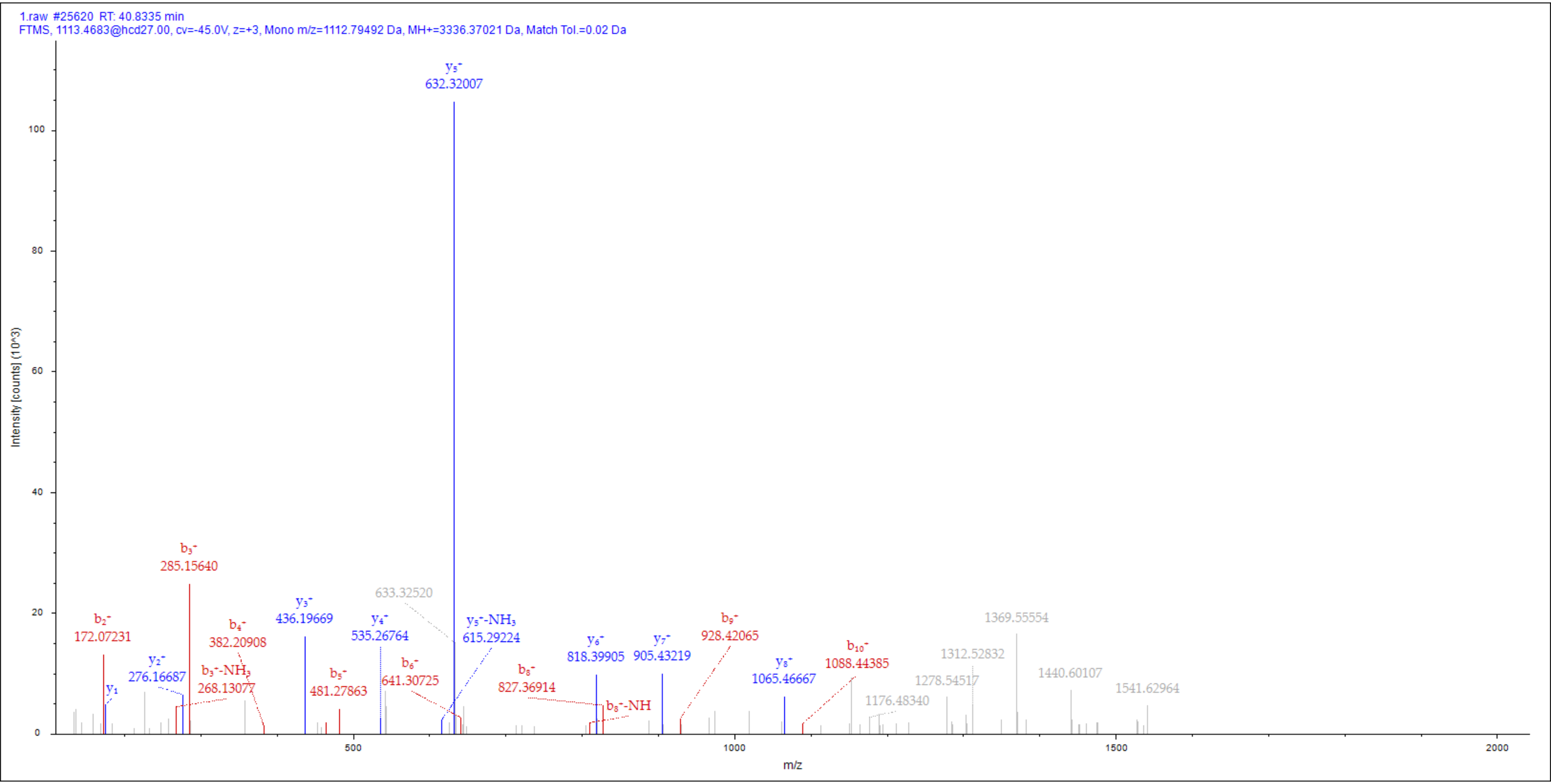

cycloviolacin_O22_NGLPICGETCVGGTCNTPGCTCSWPVCTR

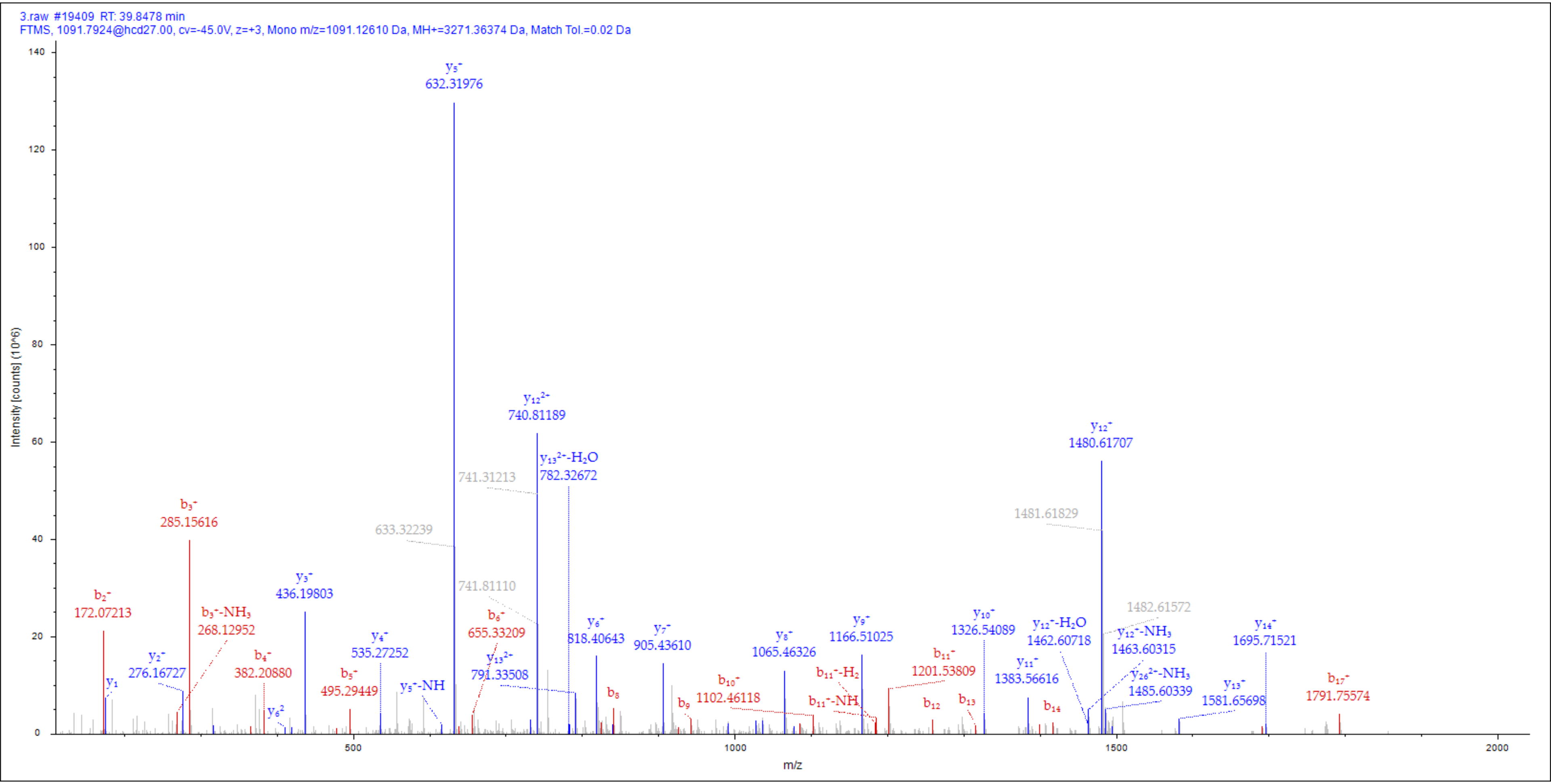

cycloviolacin_O8_NGTLPCGESCVWIPCISSVVGCSCK

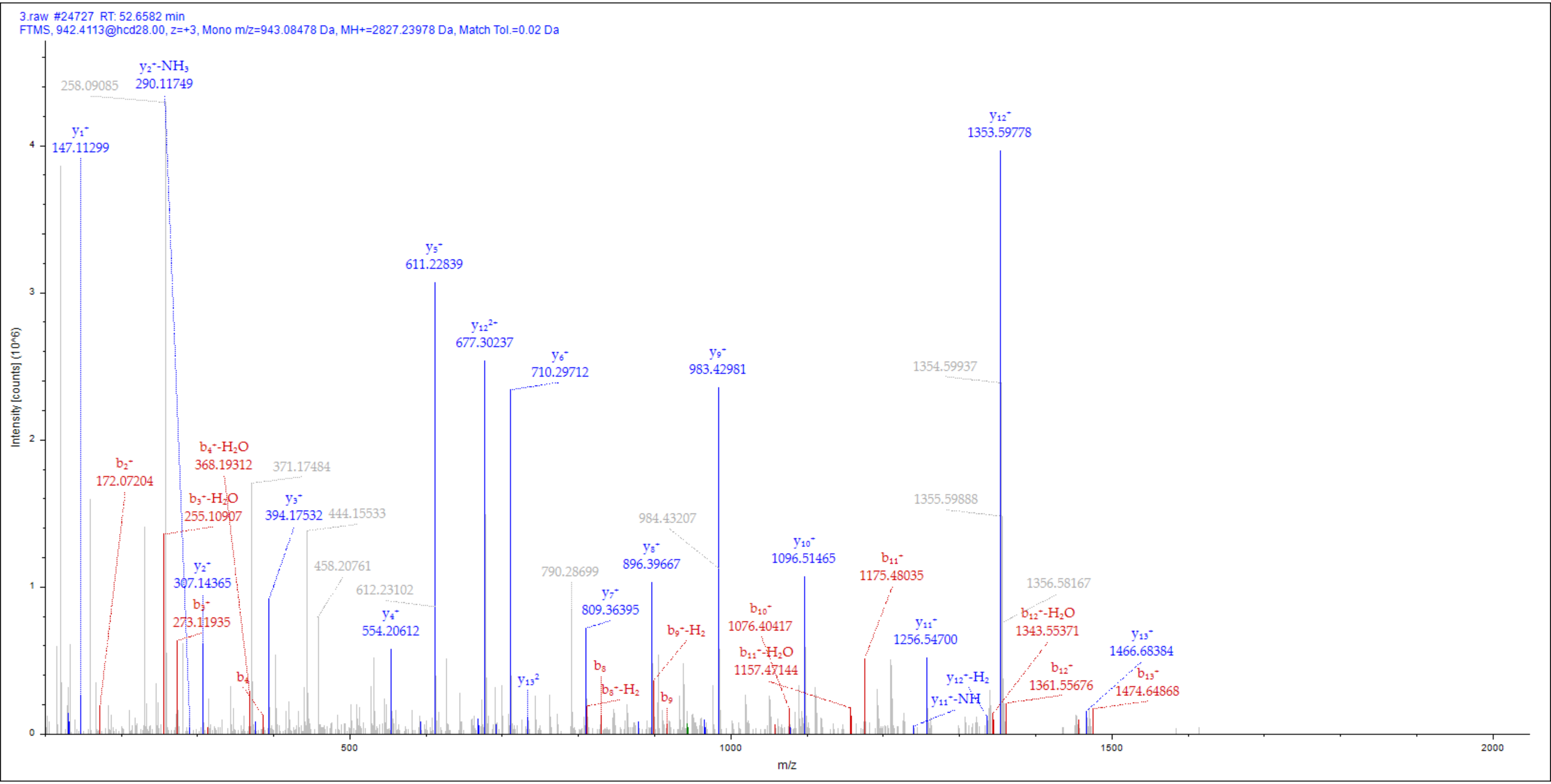

cycloviolacin_T1_NGIPVCGETCVGGTCNTPGCSCSWPVCTR

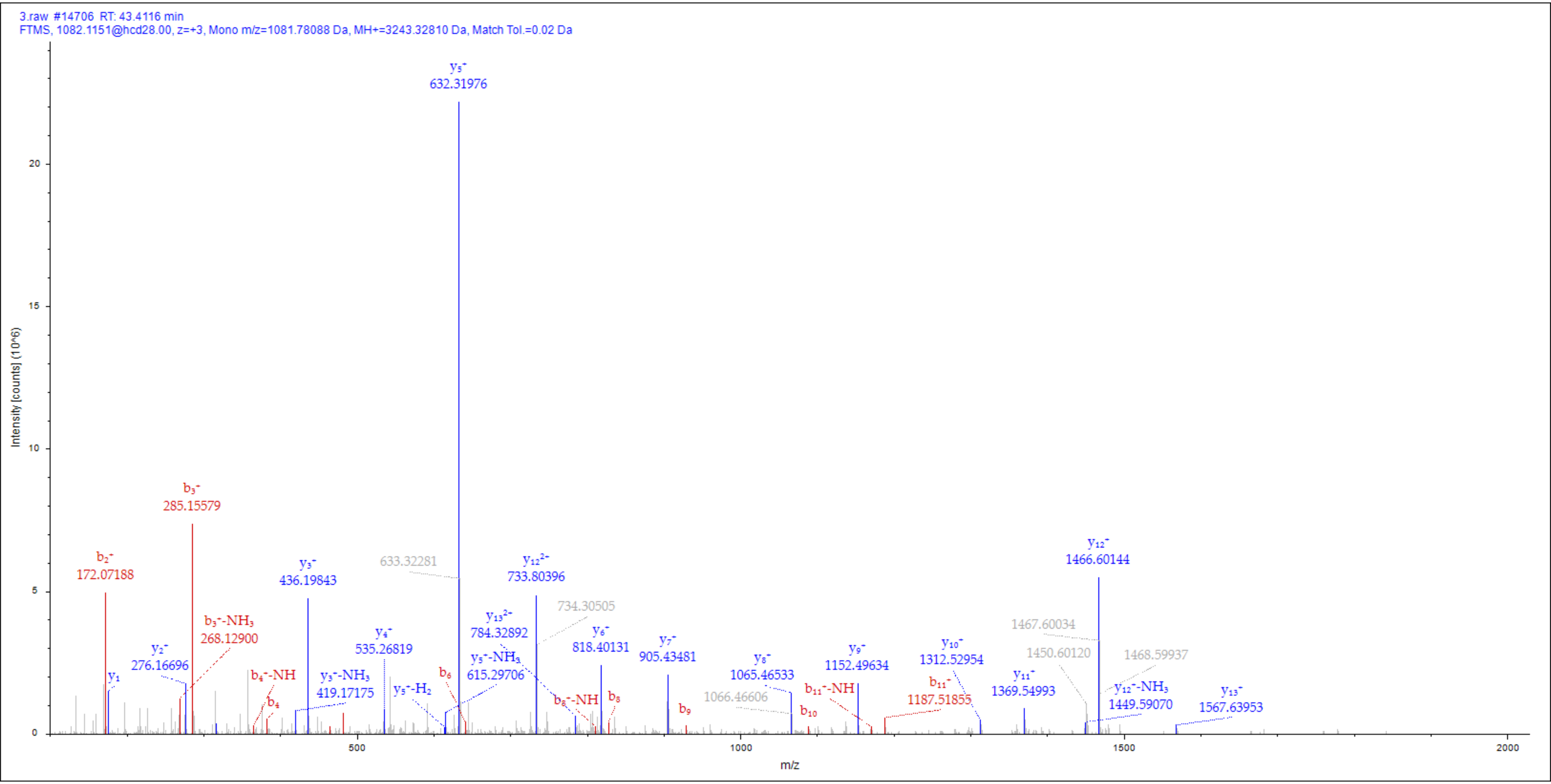

cycloviolacin_Y5_VCYNGIPCAESCVWIPCTVTALVGCSCSDK

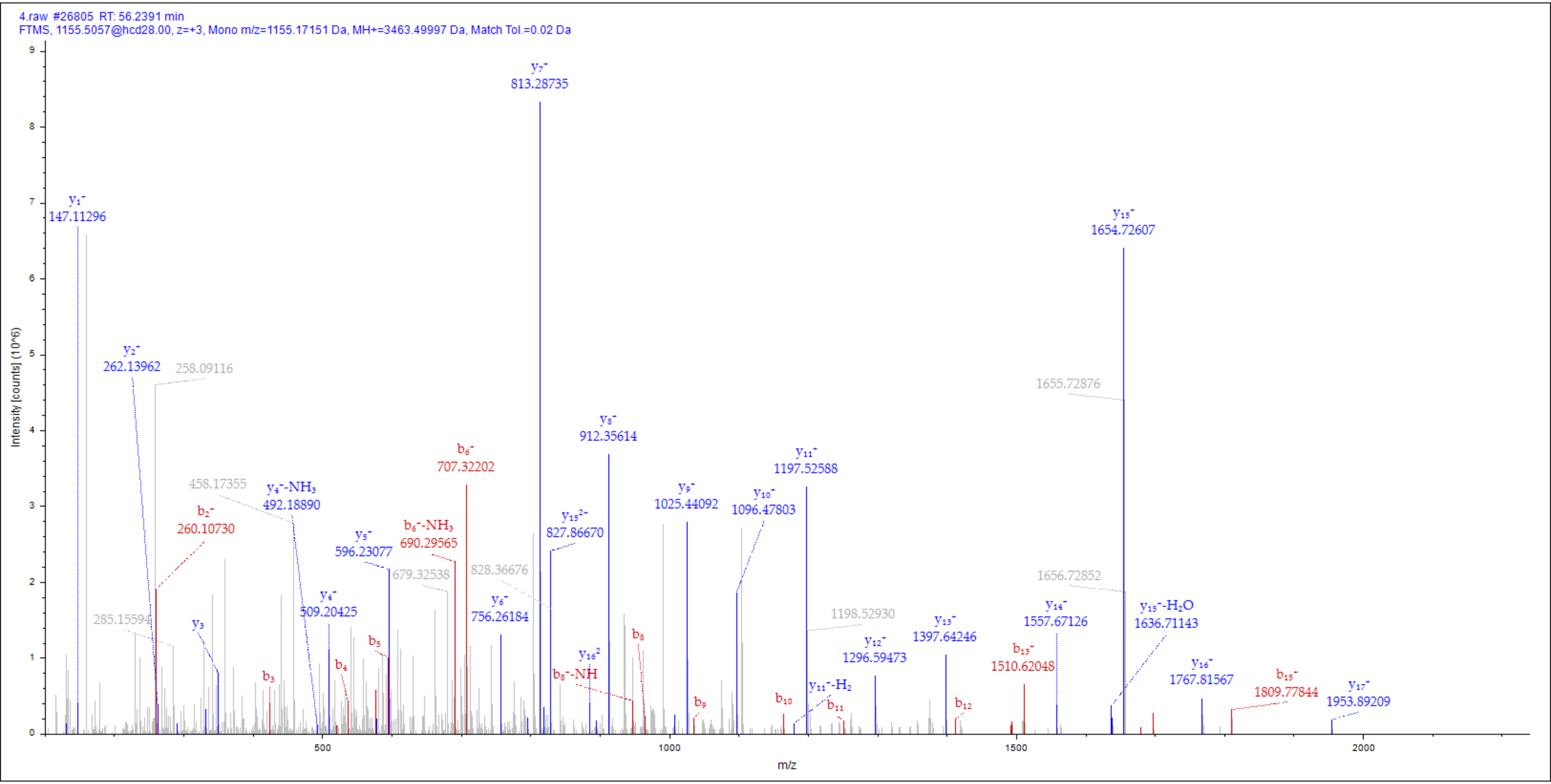

cyl2_NGTFPCGESCVWIPCISSVVGCSCK

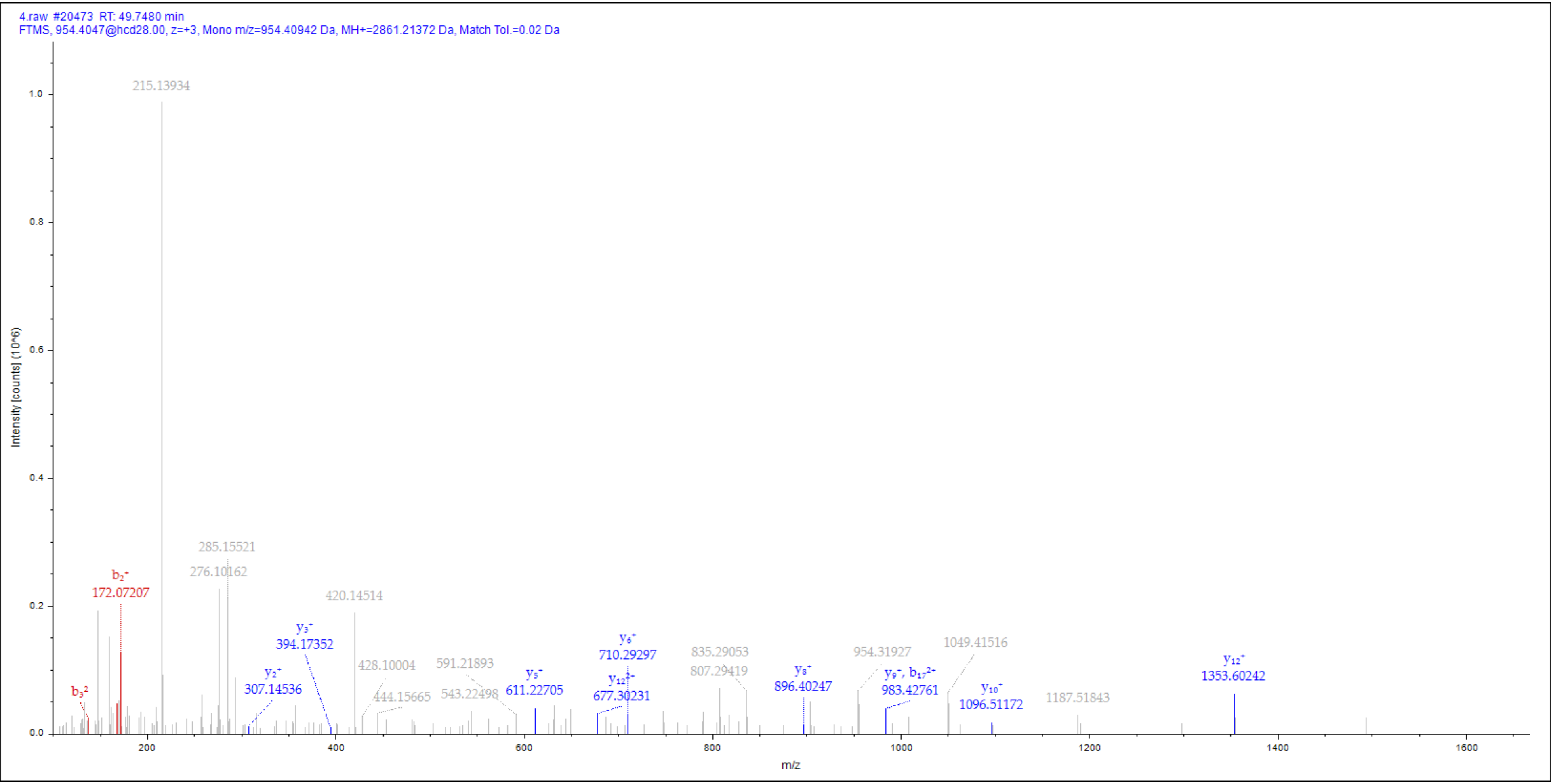

Globa_D_NGIPCGETCVFMPCISGPMGCSCK

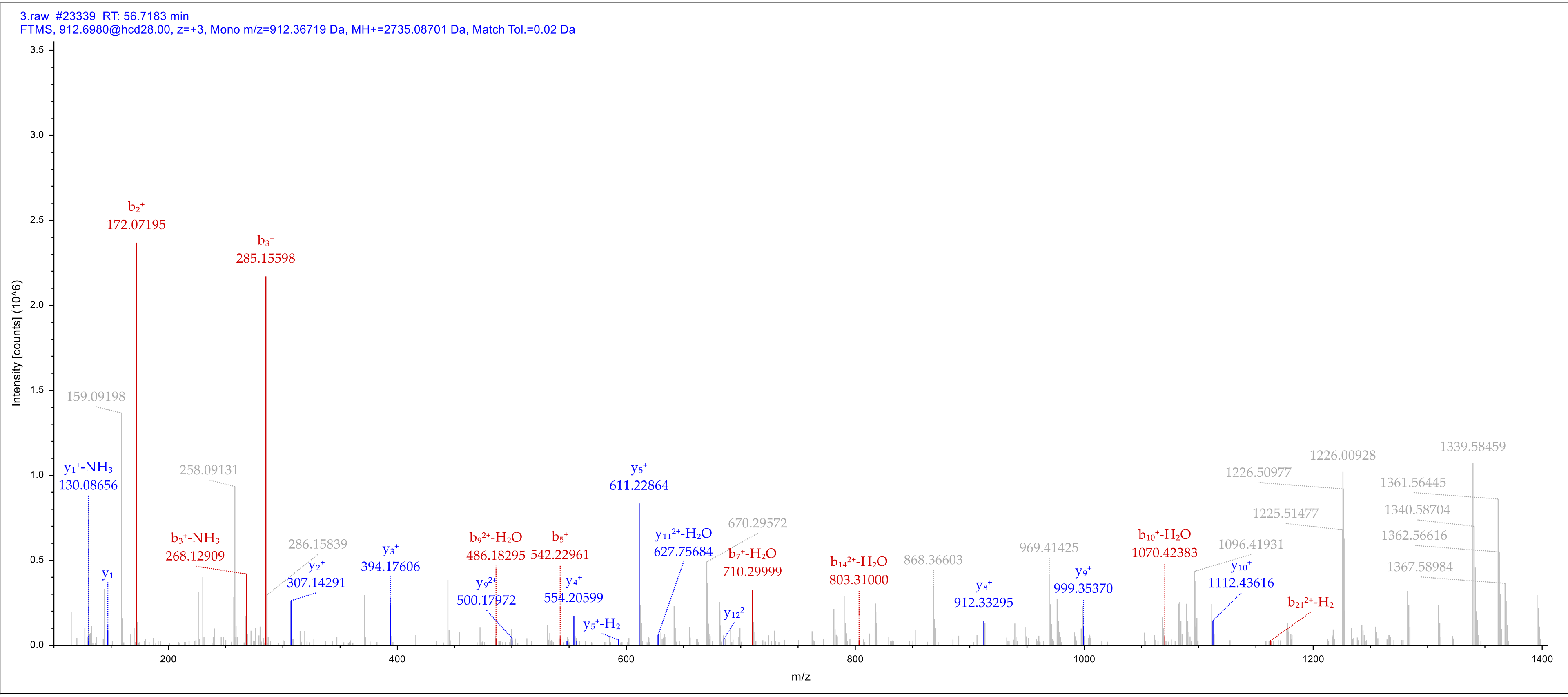

hyen_AH_NGIIPCGESCVWIPCTVTAVMGCSCK

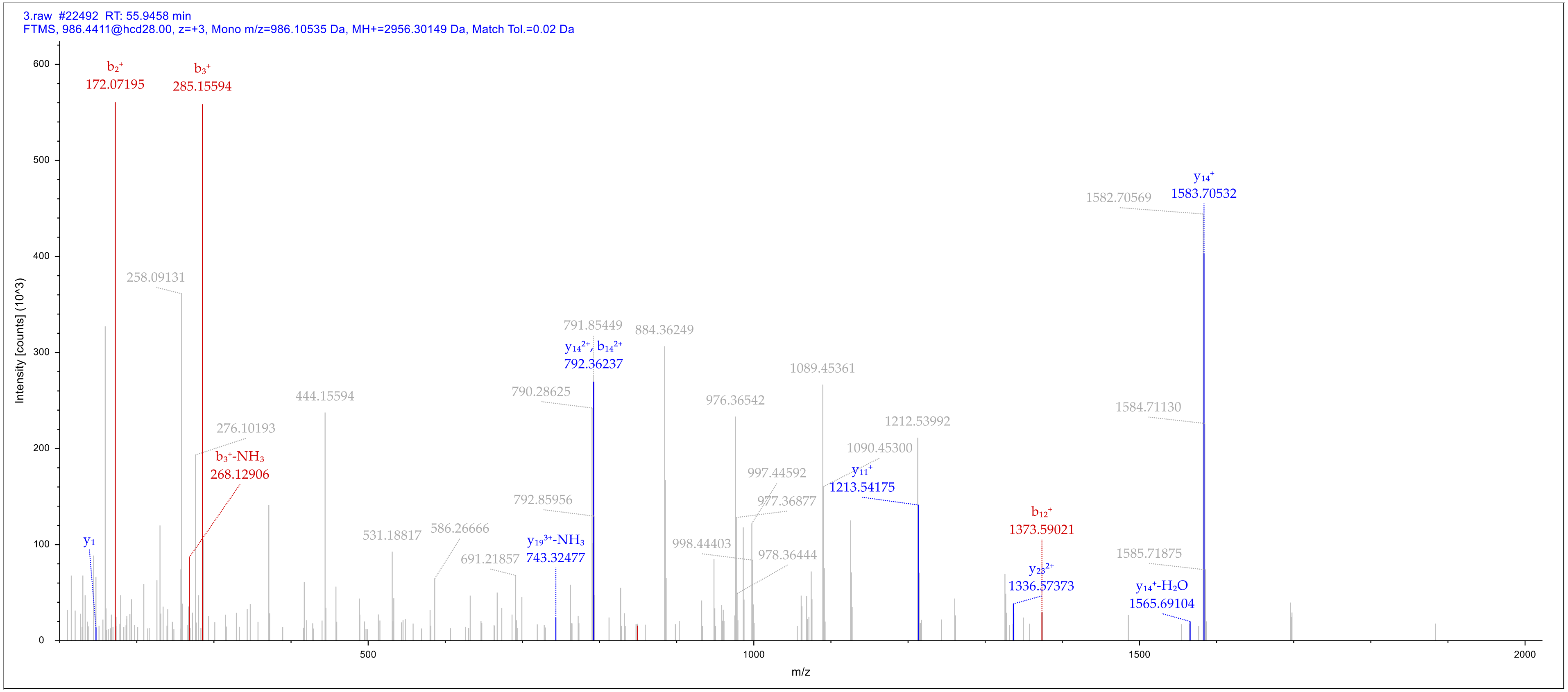

hyen_I_VCYMDGSTPCGESCVWIPCISGIVGCSCSNK

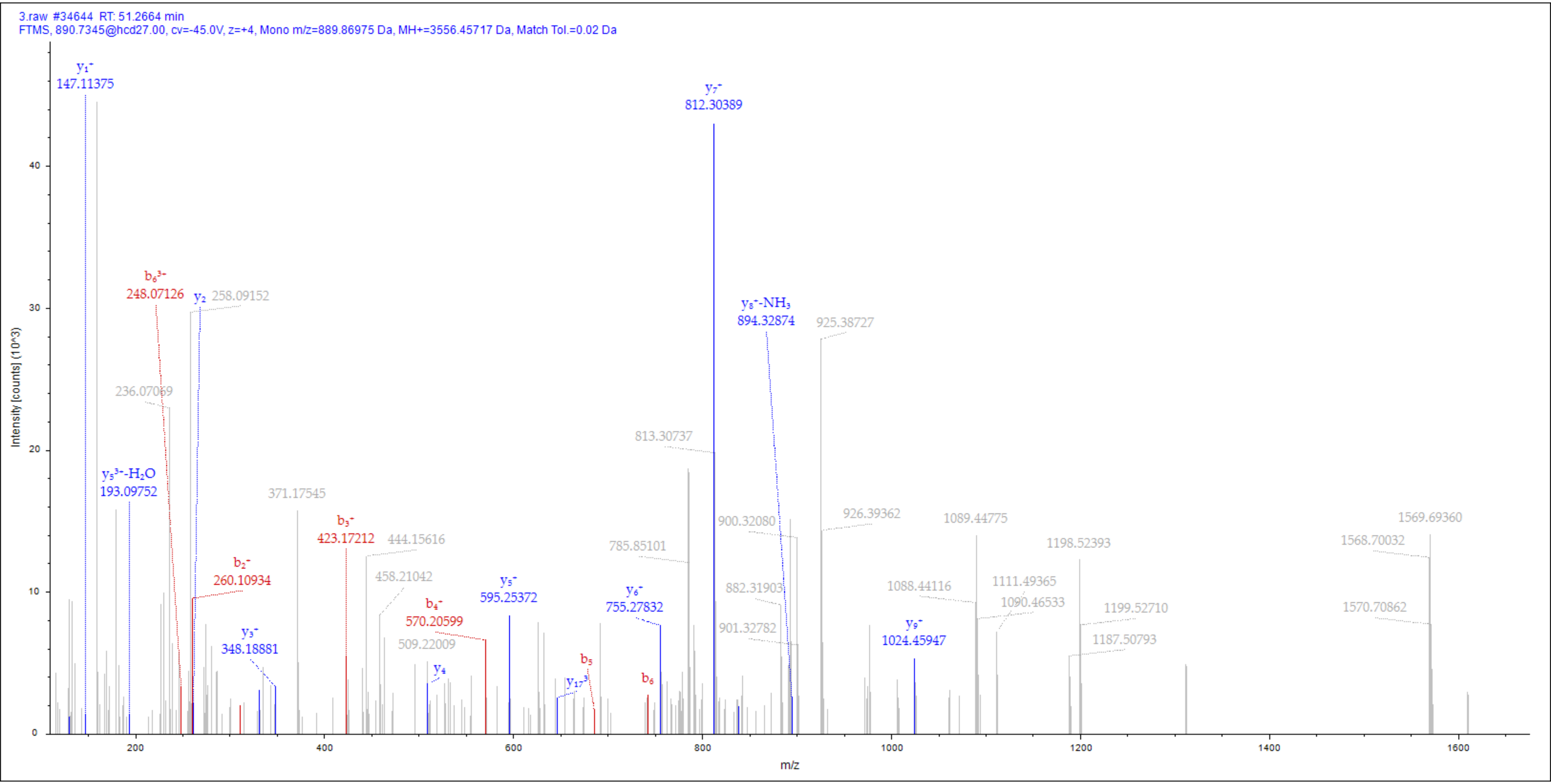

hyen_J_VCYMDGSVPCGESCVWIPCITSIAGCSCSNK

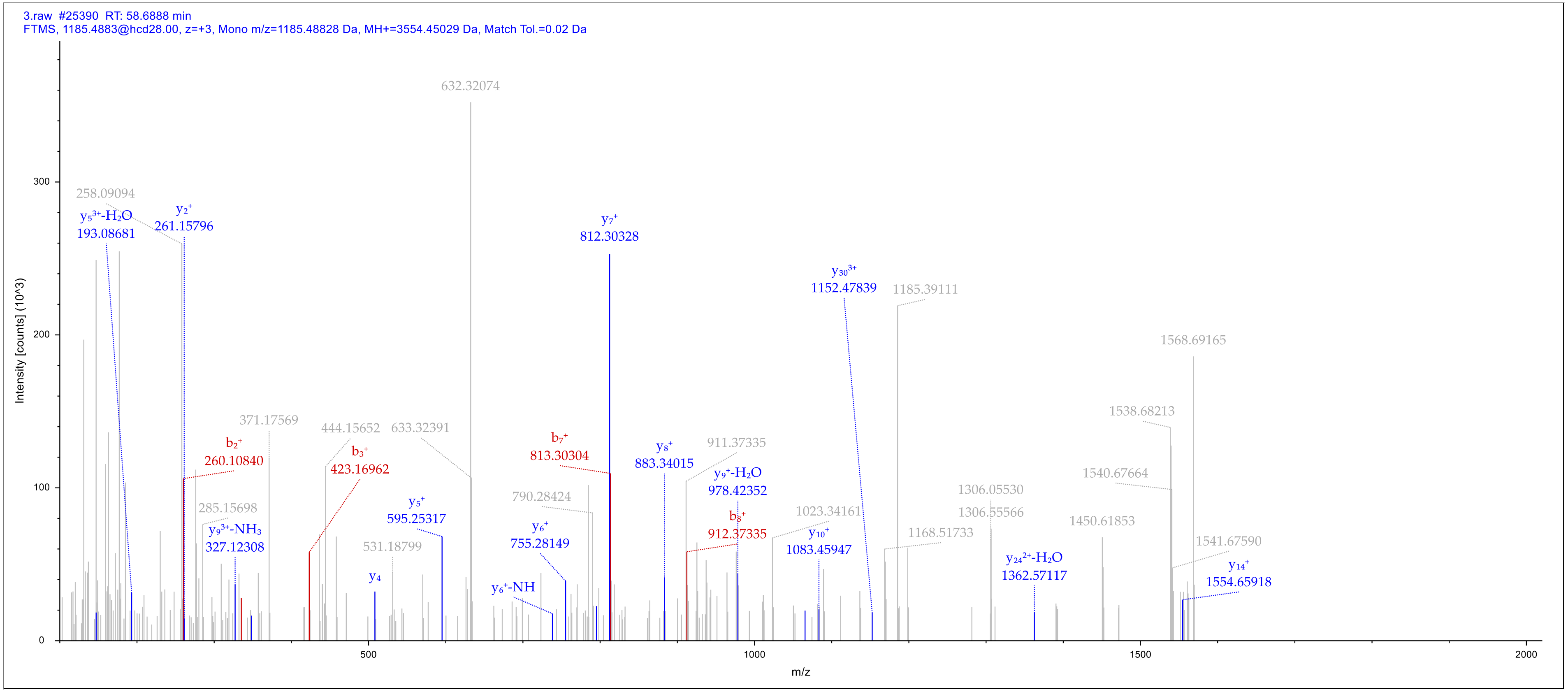

hyen_S_NGIPCGESCVYIPCFTAAIGCSCSNK

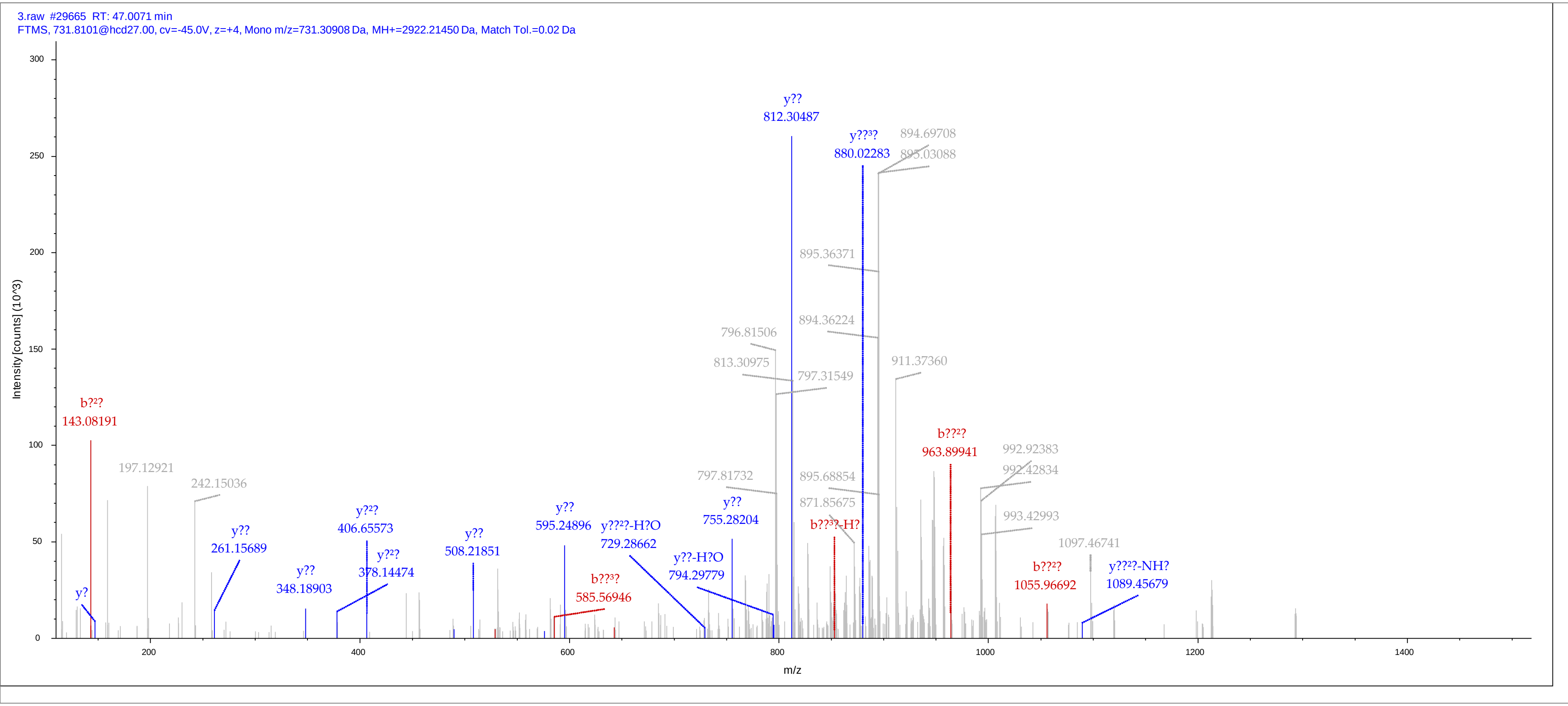

hyen_Y_NGIPCGESCVFIPCFTSAIGCSCK

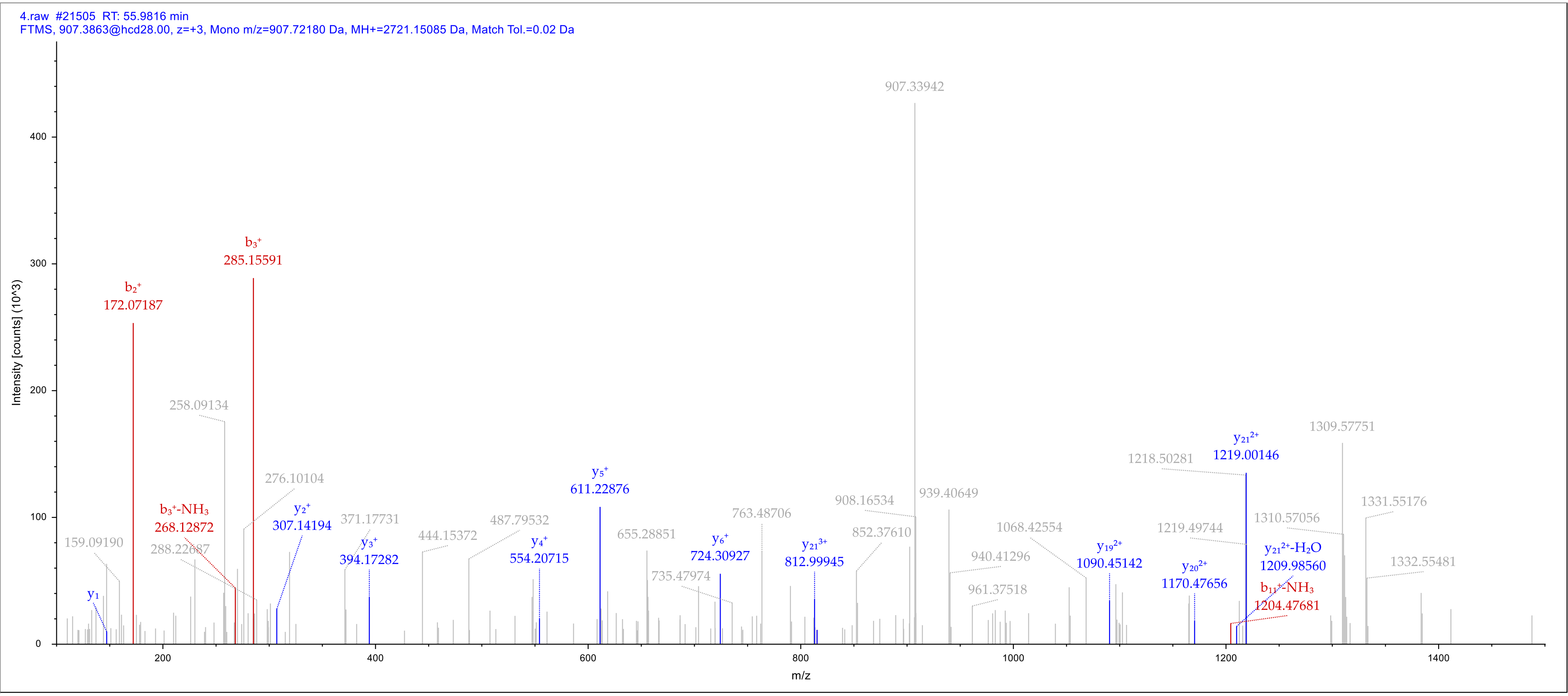

kalata_B1_NGLPVCGETCVGGTCNTPGCTCSWPVCTR

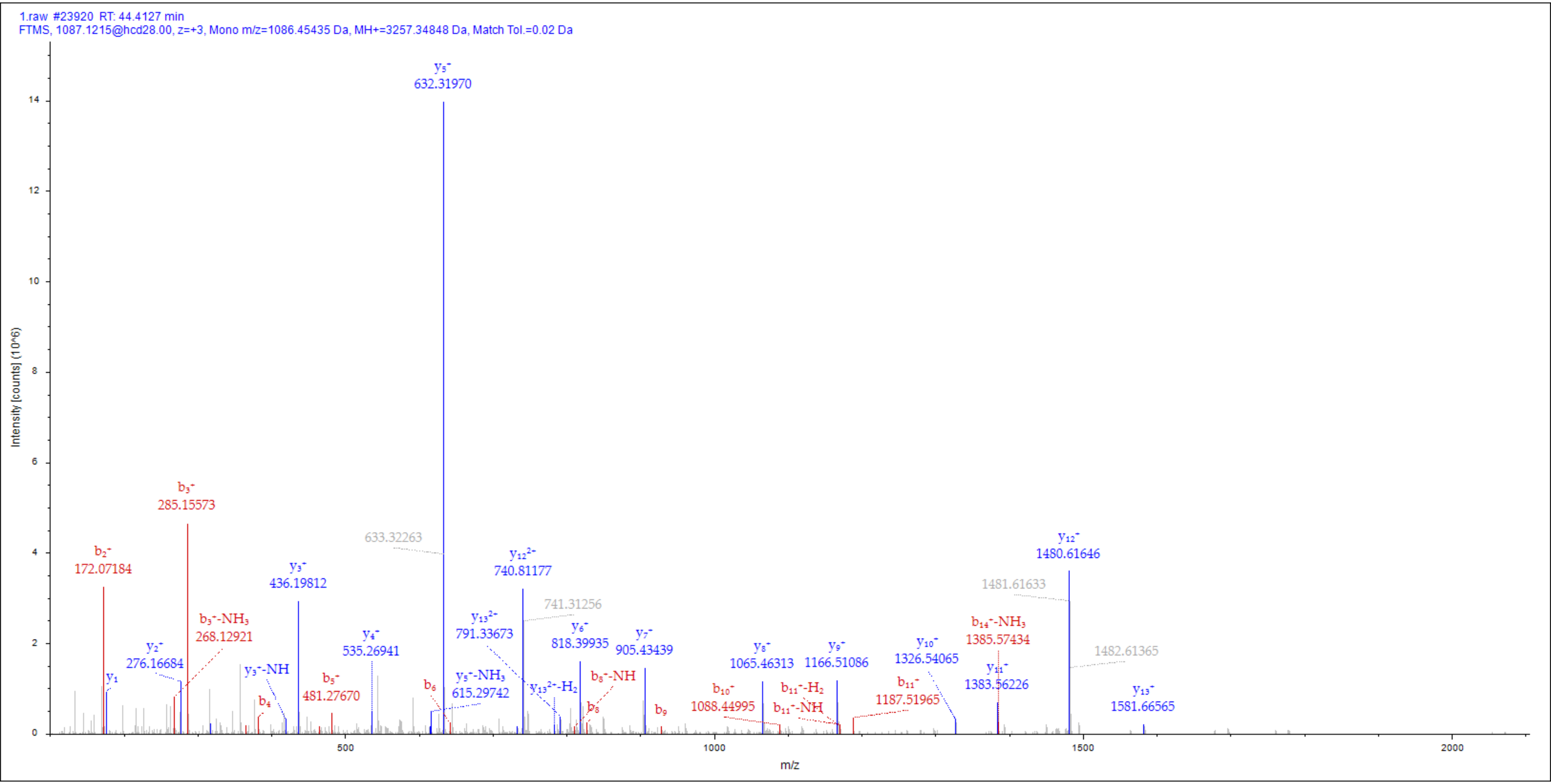

kalata_B4_DGLPVCGETCVGGTCNTPGCTCSWPVCTR

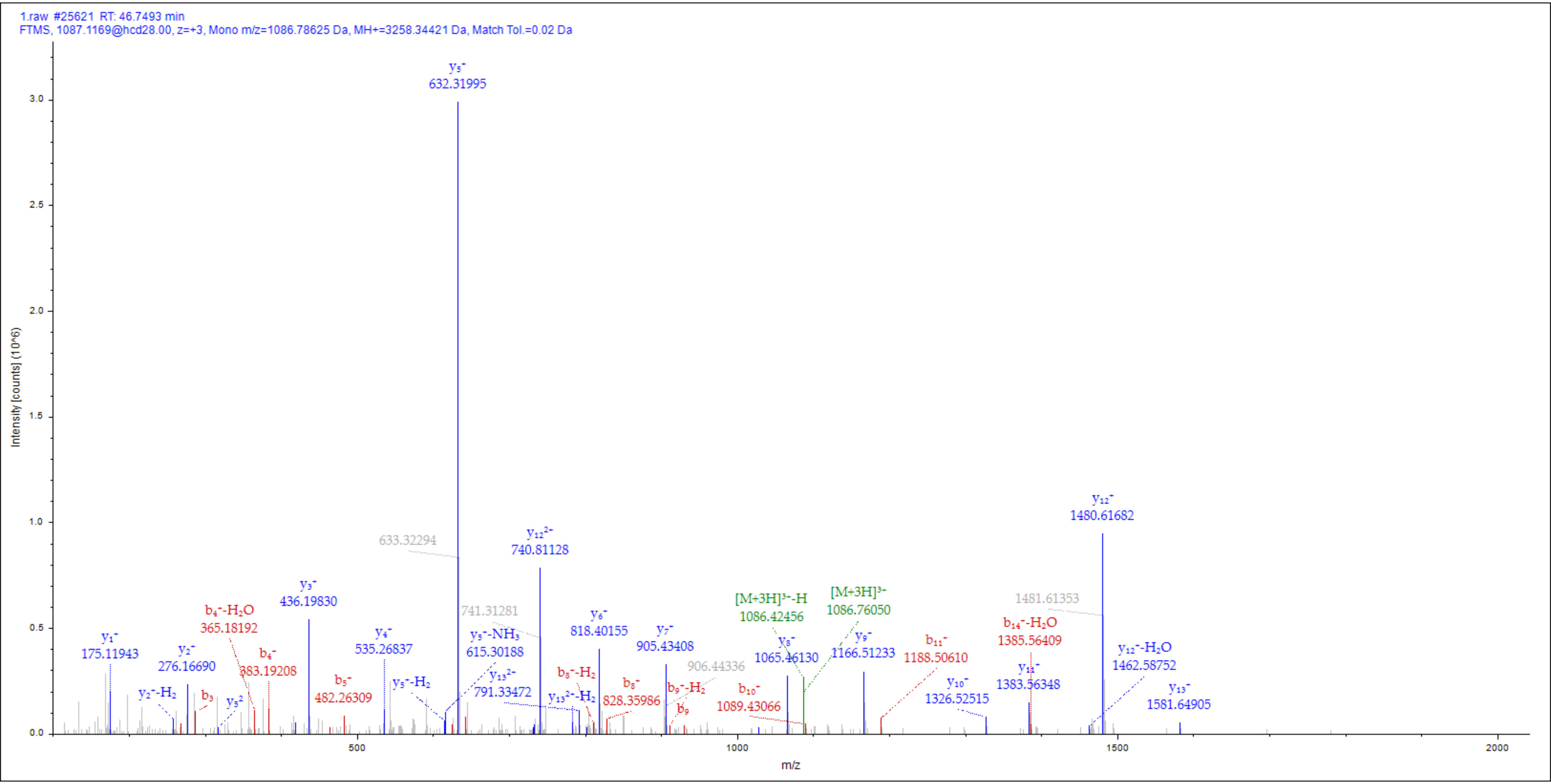

mech_7_DGIPICGETCTIGTCNTPGCTCSWPVCTR

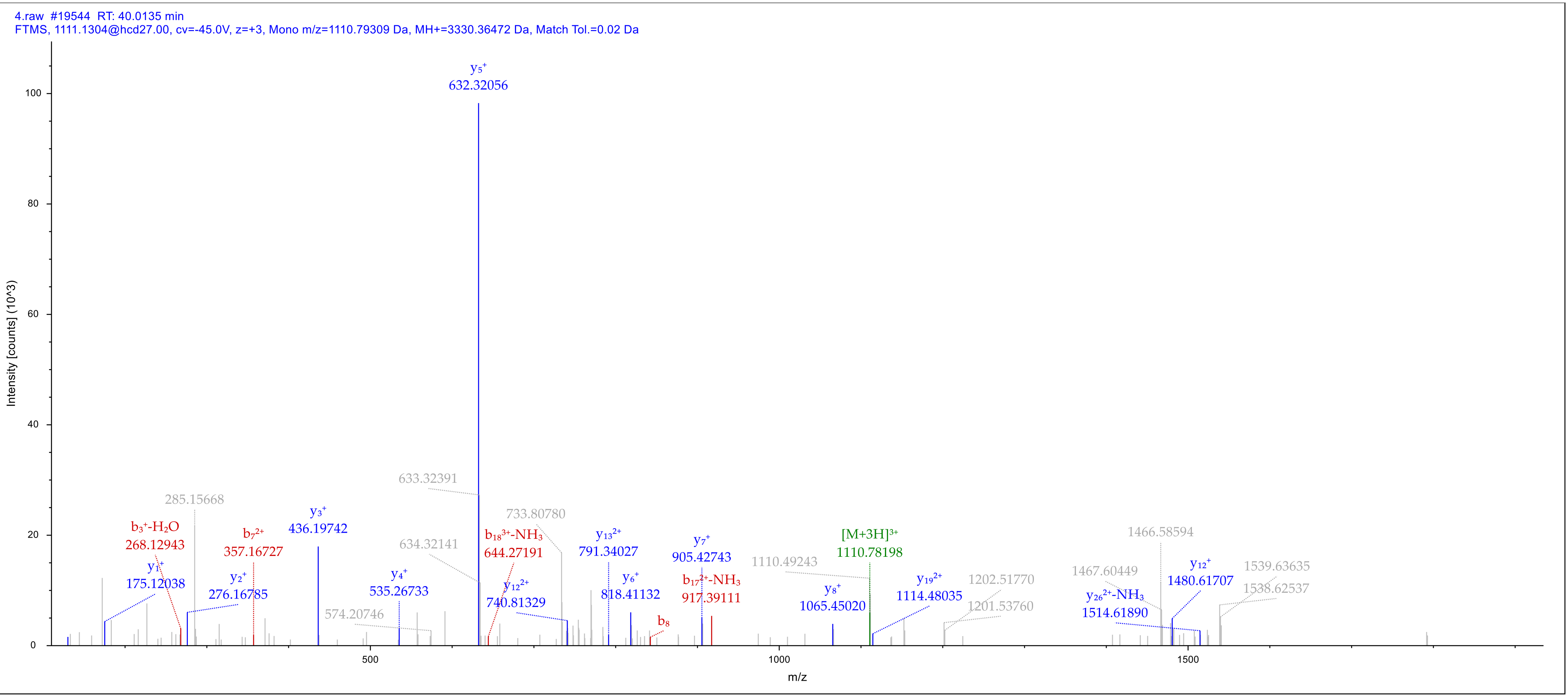

Mema_A_NGLPCAESCVWLPCTVTALLGCSCK

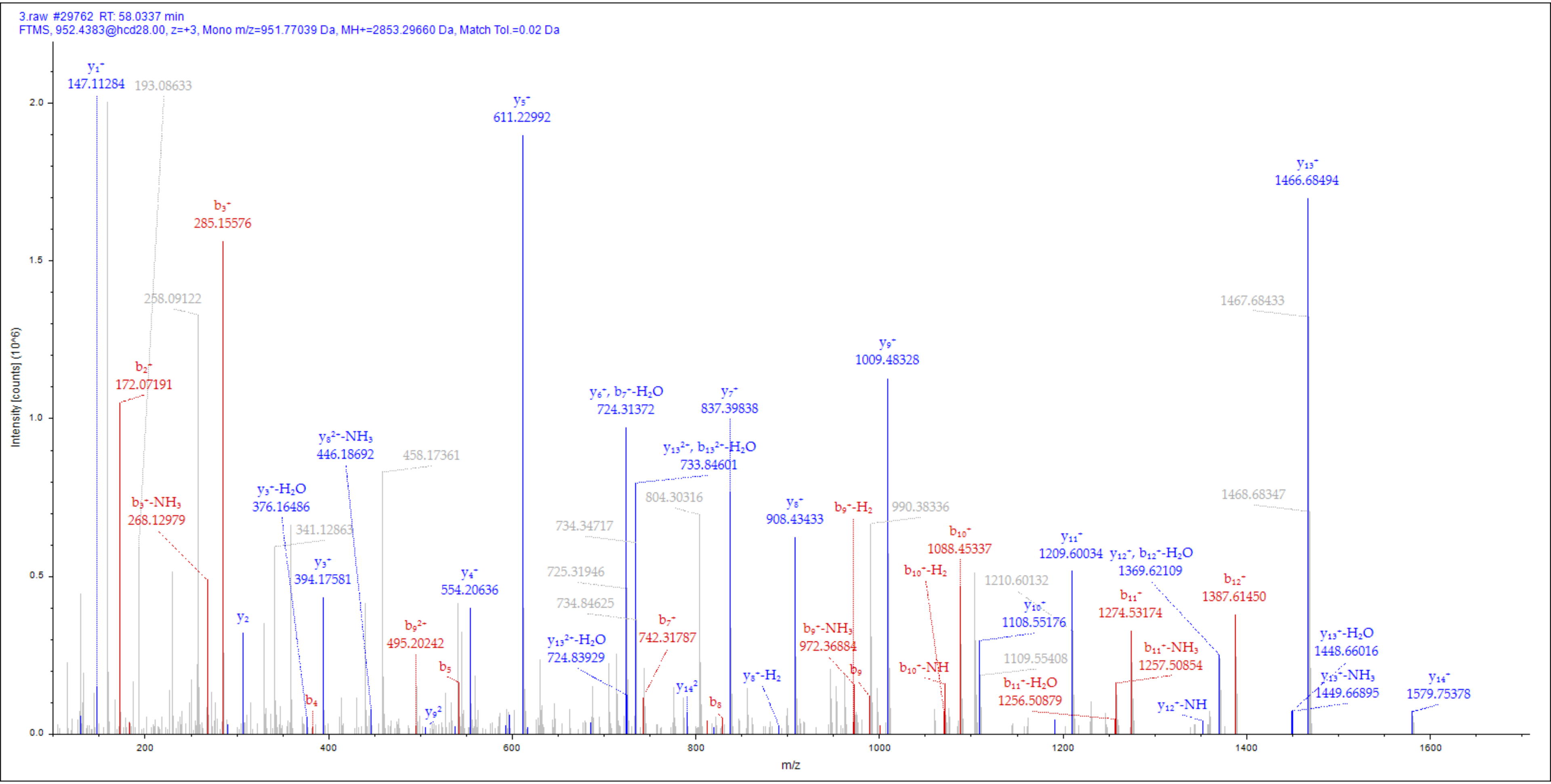

Mra30_NGIPCGESCVFIPCLTSAIGCSCK

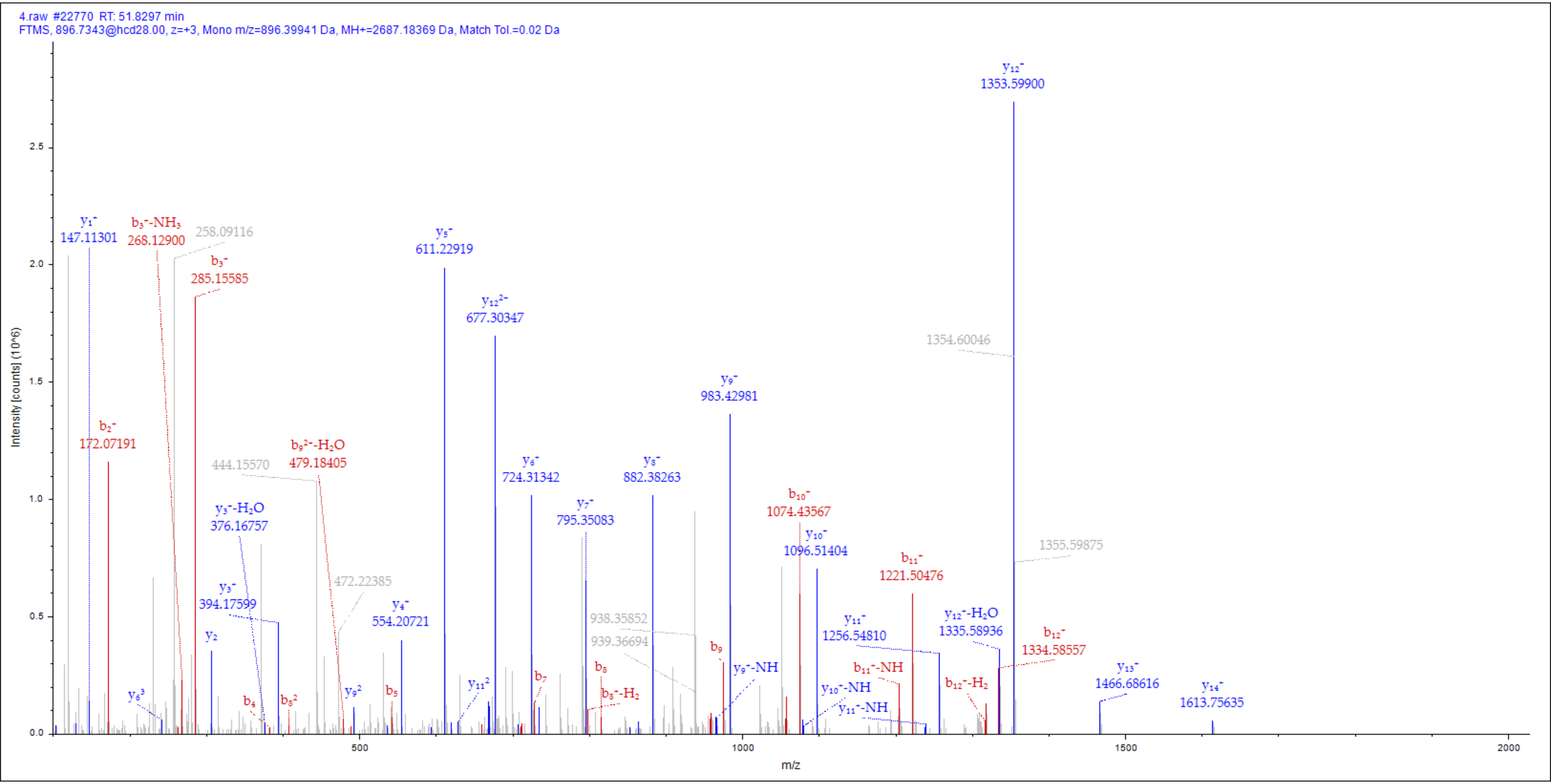

paltet_1_NGLPICGETCFTGTCNTPGCTCSYPVCTR

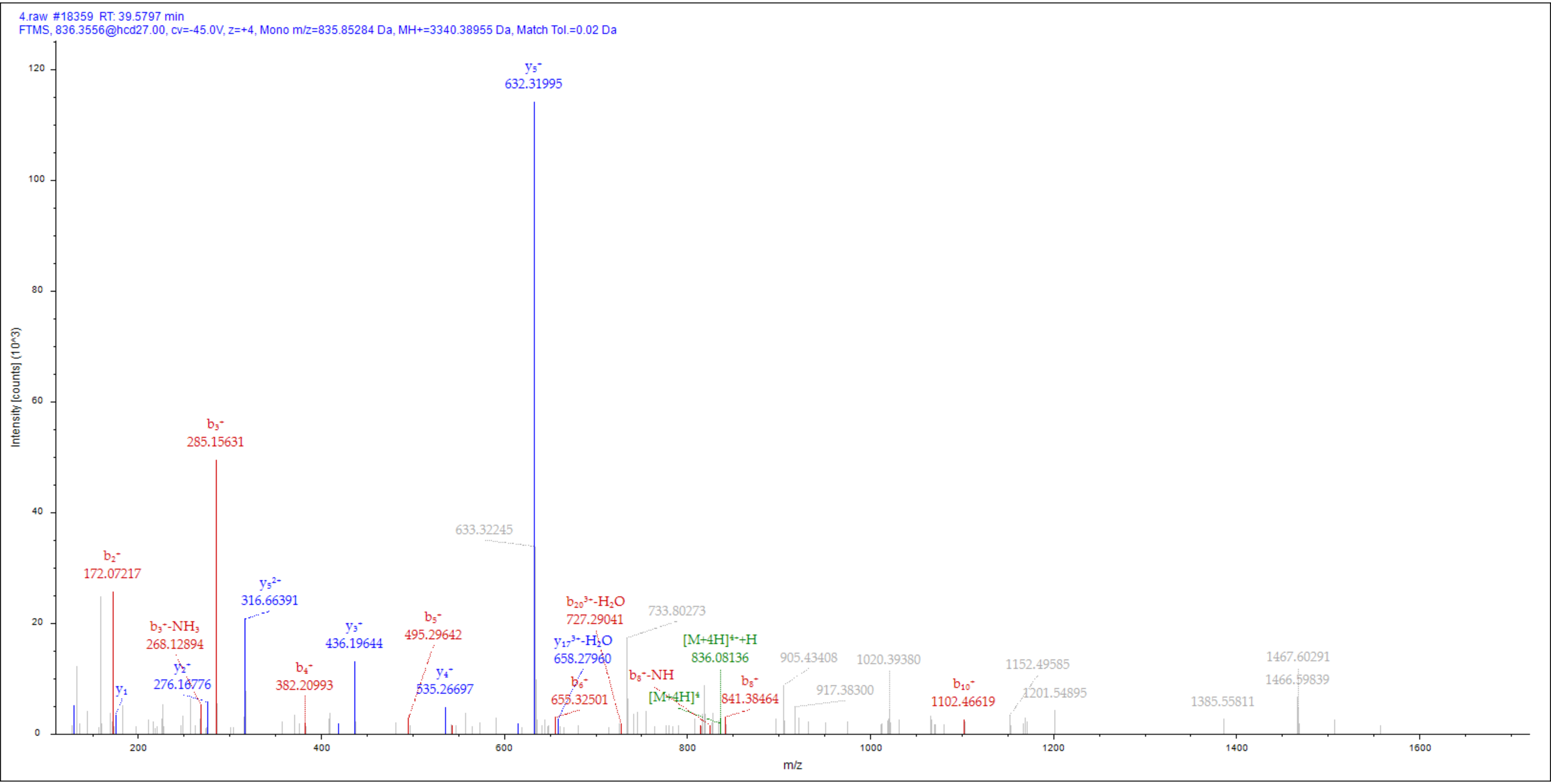

pase_A_NGLPVCGETCVGGTCNTPGCVCSWPVCTR

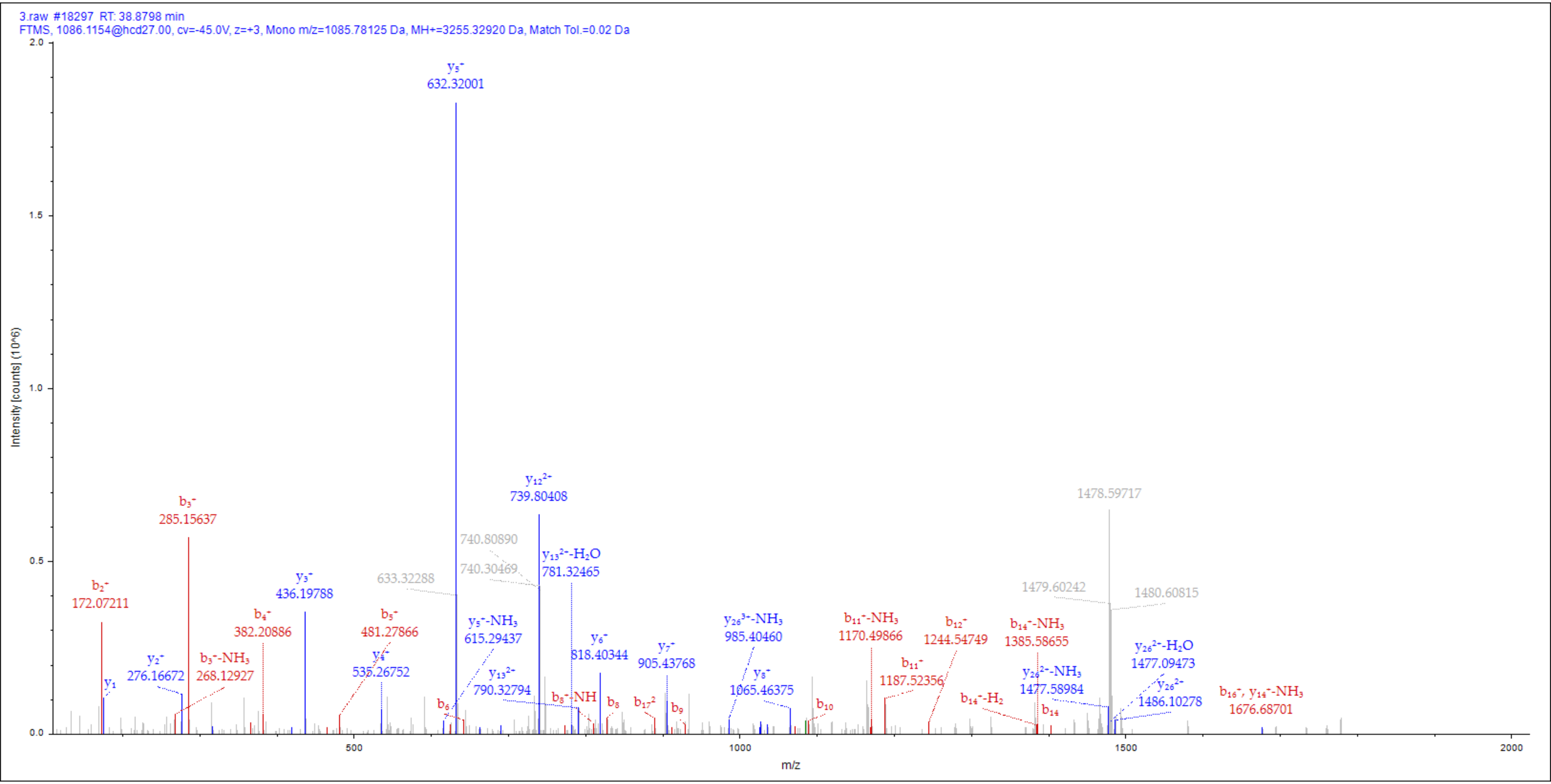

psyleio_D_DGLPVCGESCFGGTCNTPGCSCTWPVCTR

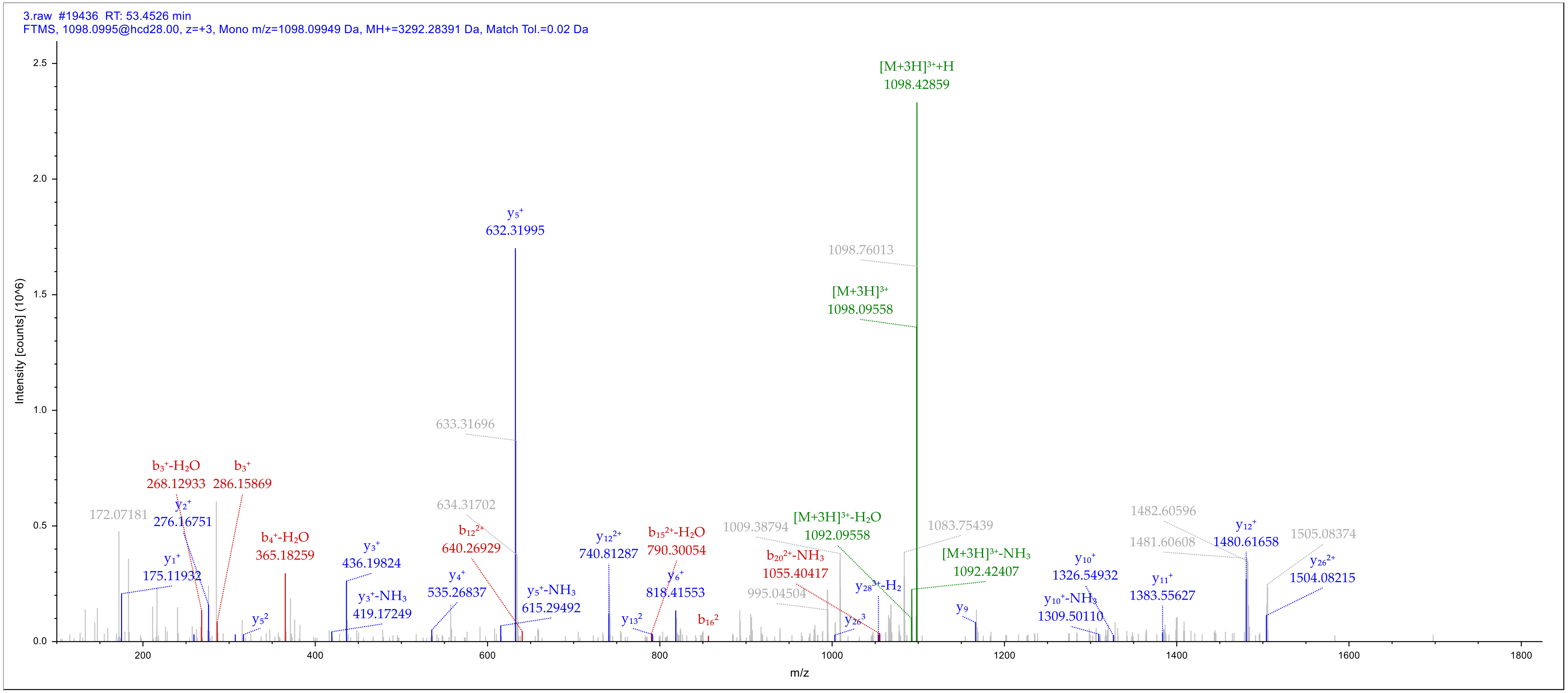

vaby_C_NGLPVCGETCAGGRCNTPGCSCSWPVCTR

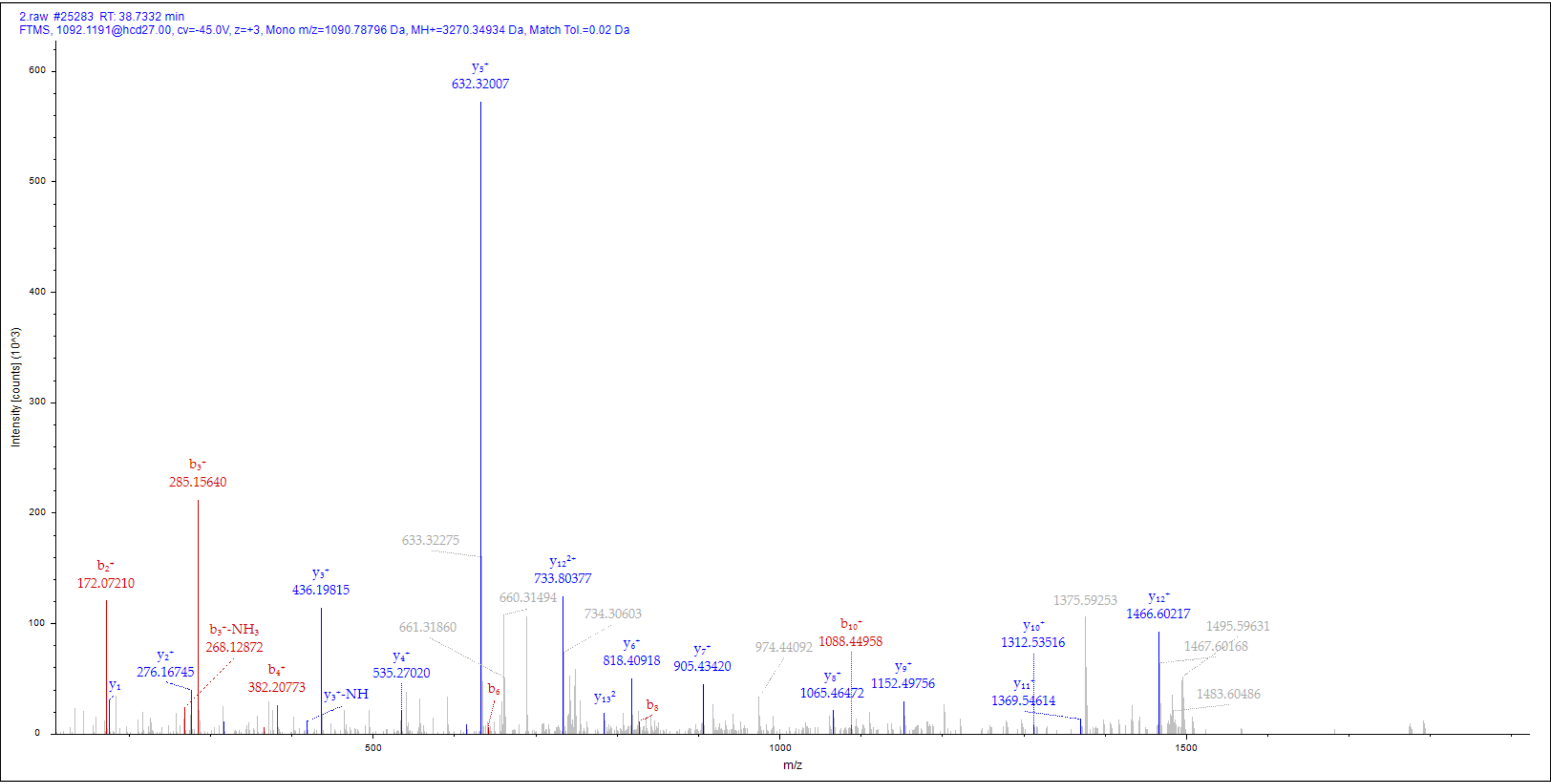

varv_peptide_D_NGLPICGETCVGGSCNTPGCSCSWPVCTR

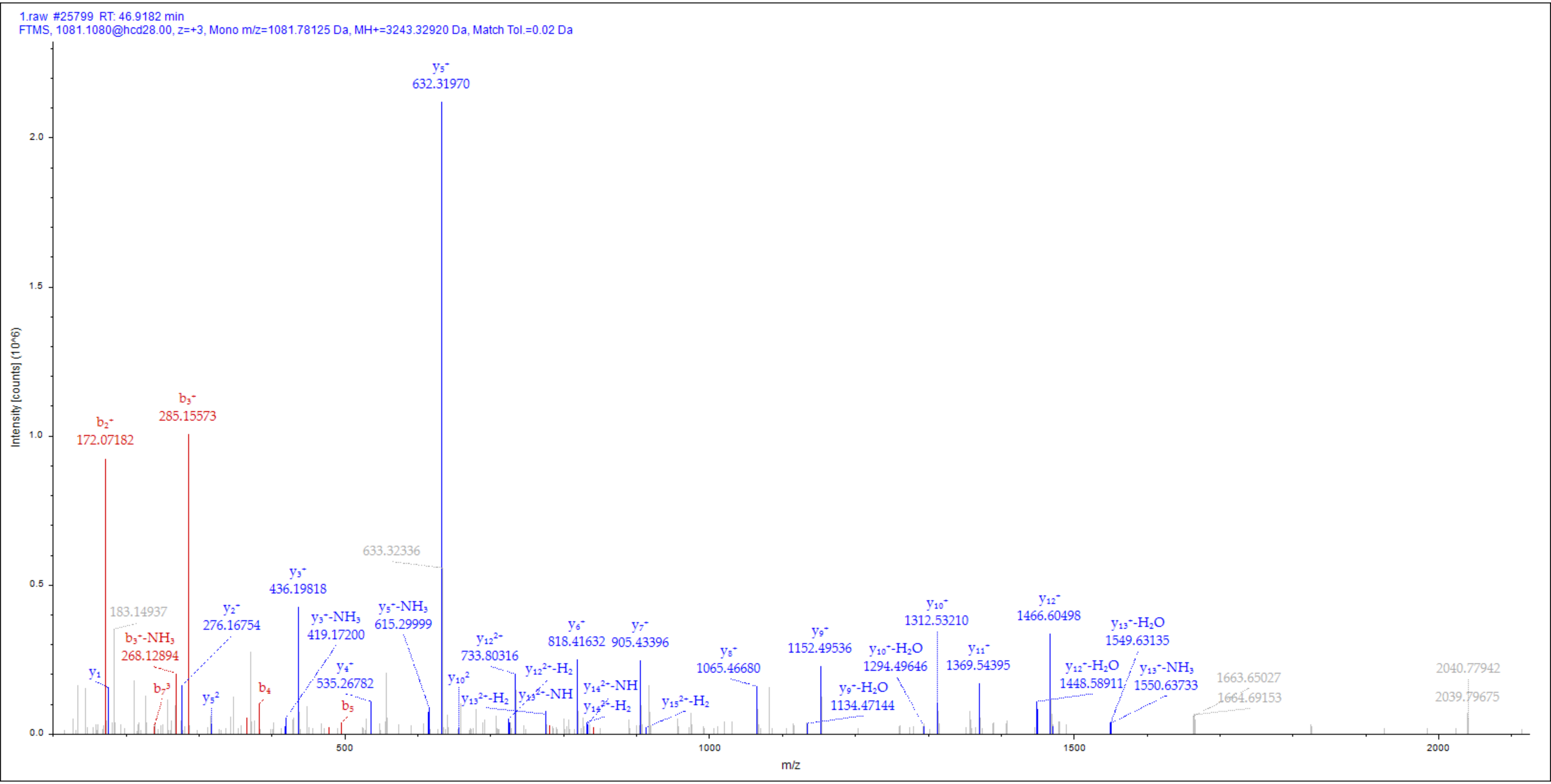

varv_peptide_F_NGVPICGETCTLGTCYTAGCSCSWPVCTR

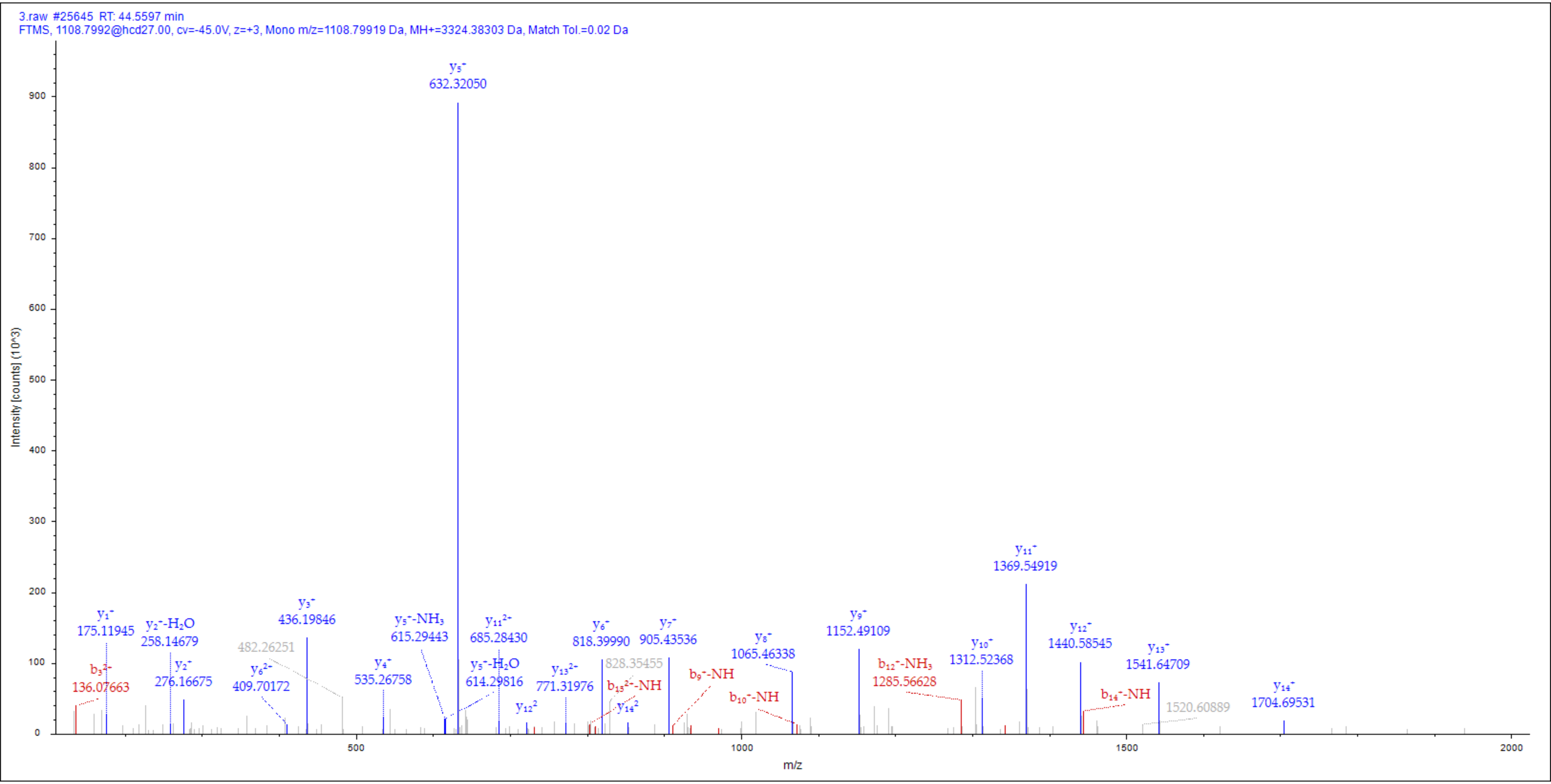

Viba_11_NGIPCGESCVWIPCISGAIGCSCK

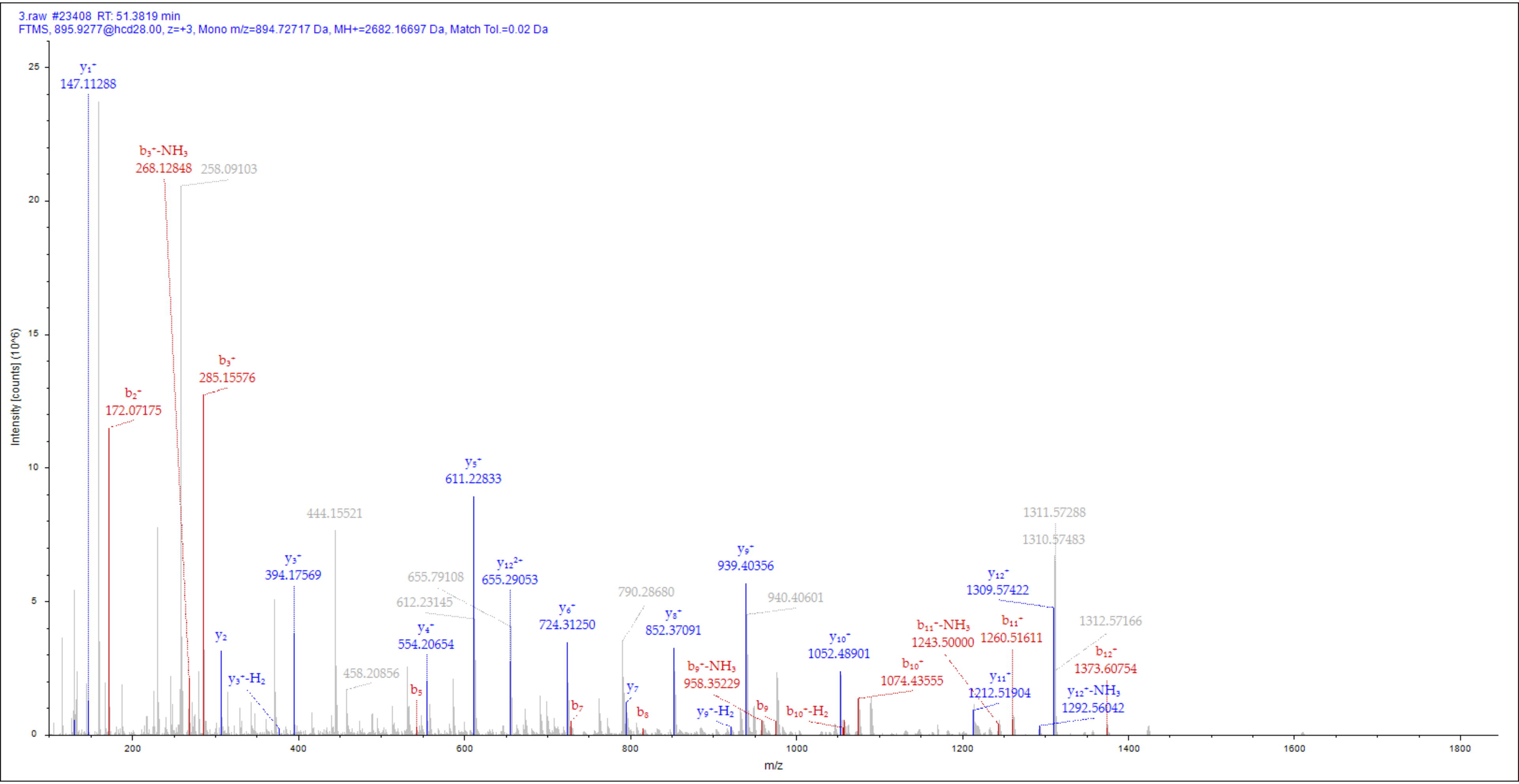

Viba_12_DKVCYNGIPCAESCVWIPCTVTALLGCSCK

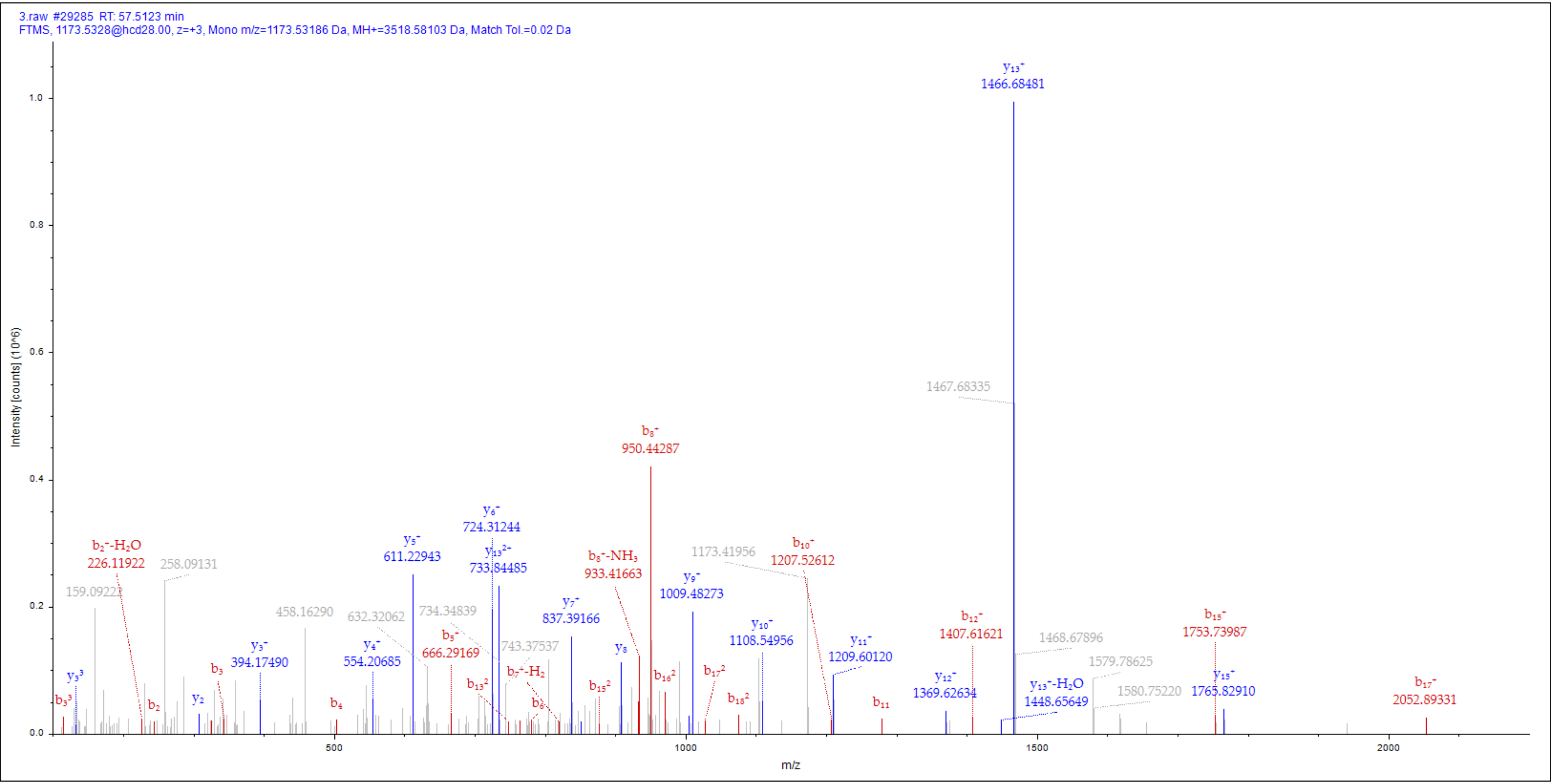

Viba_12_VCYNGIPCAESCVWIPCTVTALLGCSCK

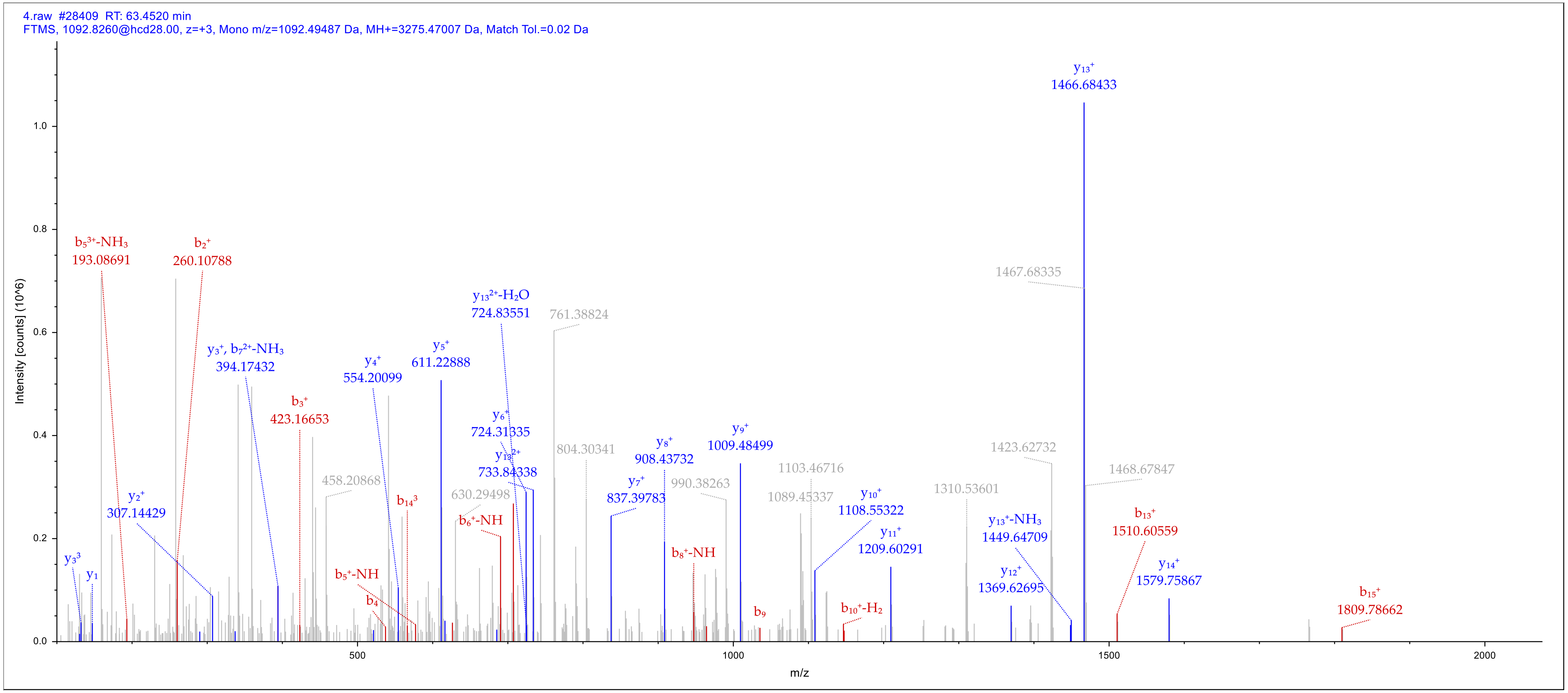

Viba_15_NGLPVCGETCVGGTCNTPGCACSWPVCTR

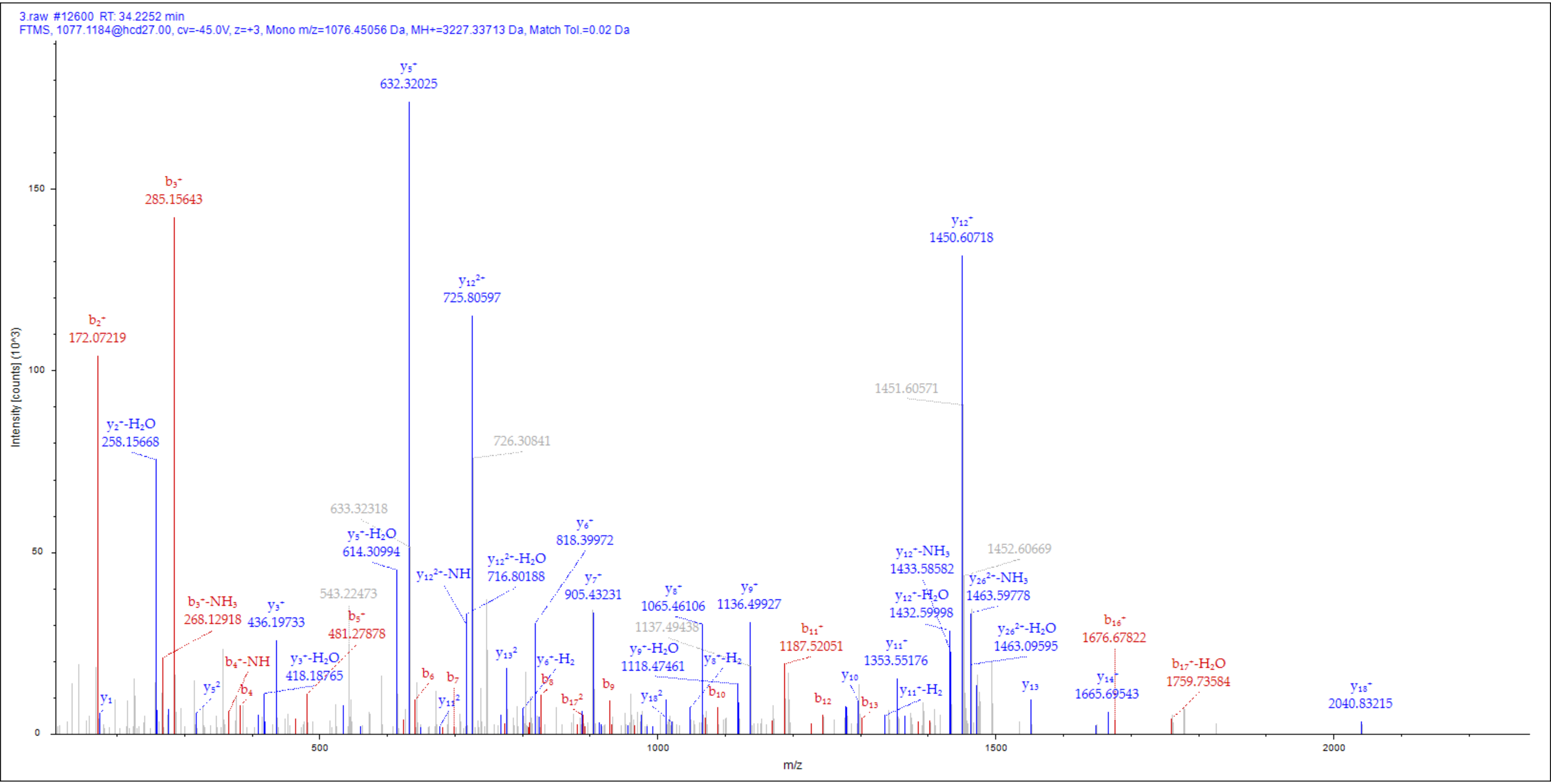

Viba_17_NGLPVCGETCVGGTCNTPGCGCSWPVCTR

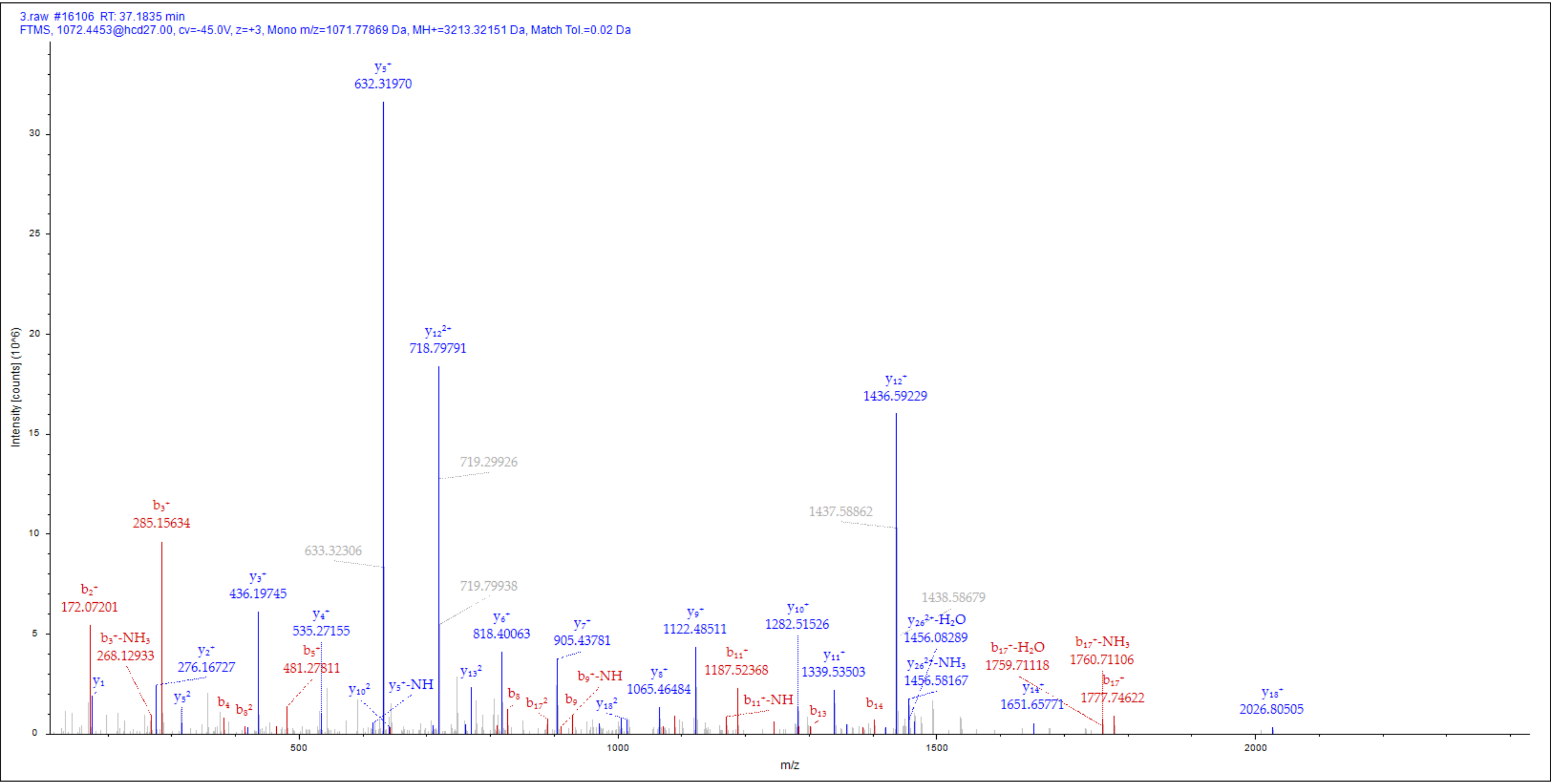

viba_32_NGLPVCGEACVGGTCNTPGCSCSWPVCTR

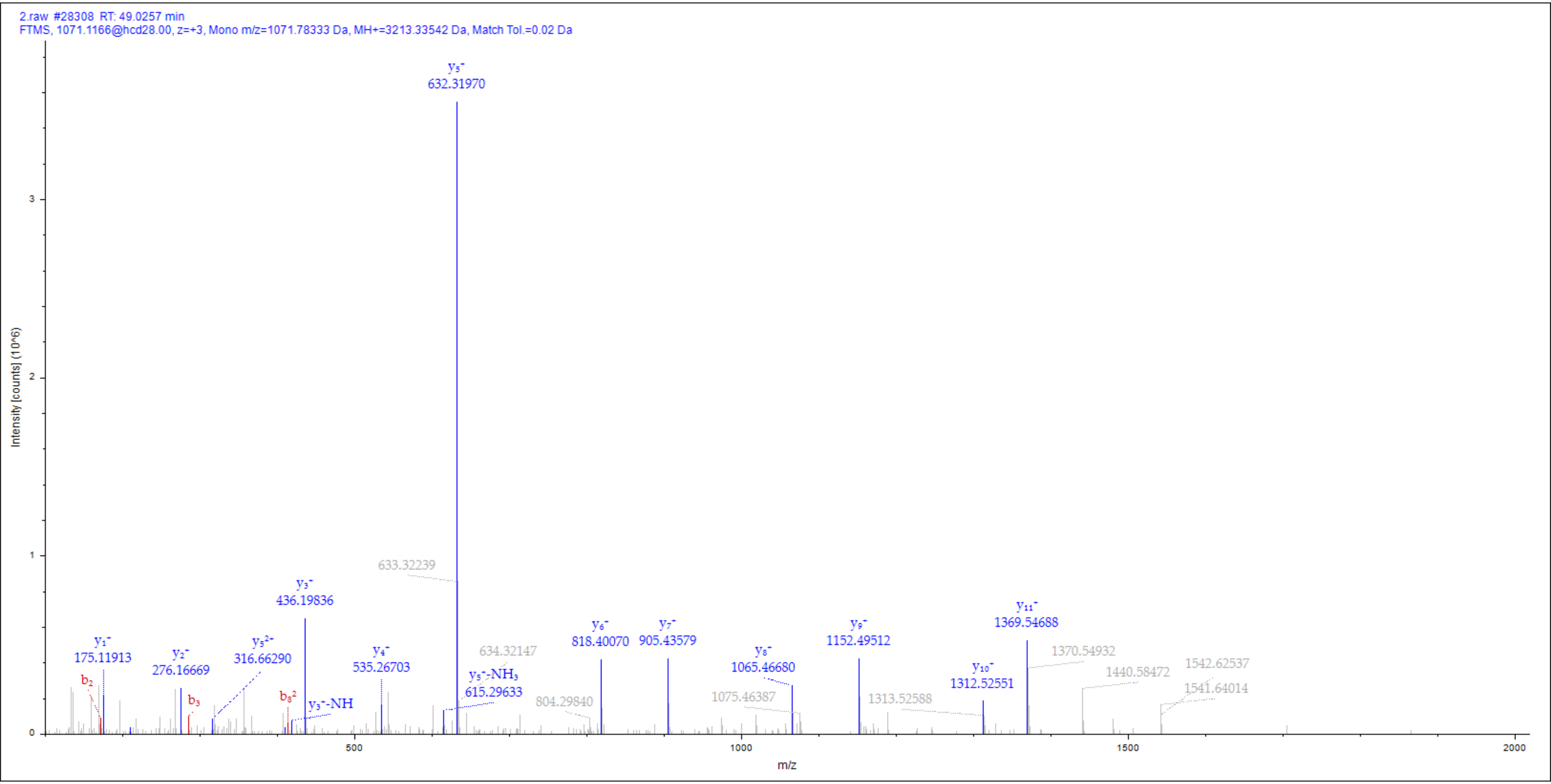

Viba_7_NGVIPCGESCVFIPCISSVIGCSCK

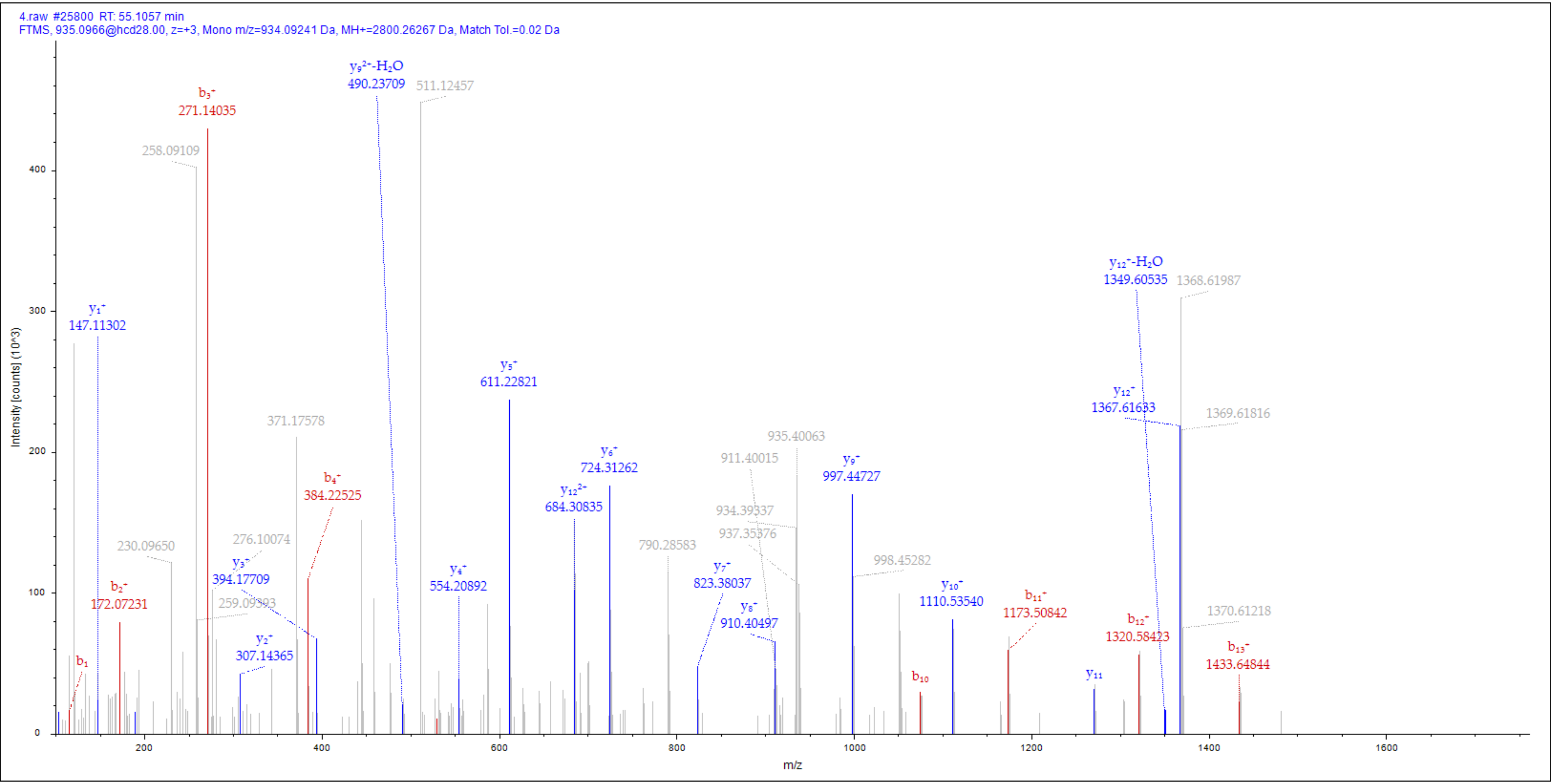

Viba19_NGLPVCGETCFGGTCNTPGCSCEWPVCTR

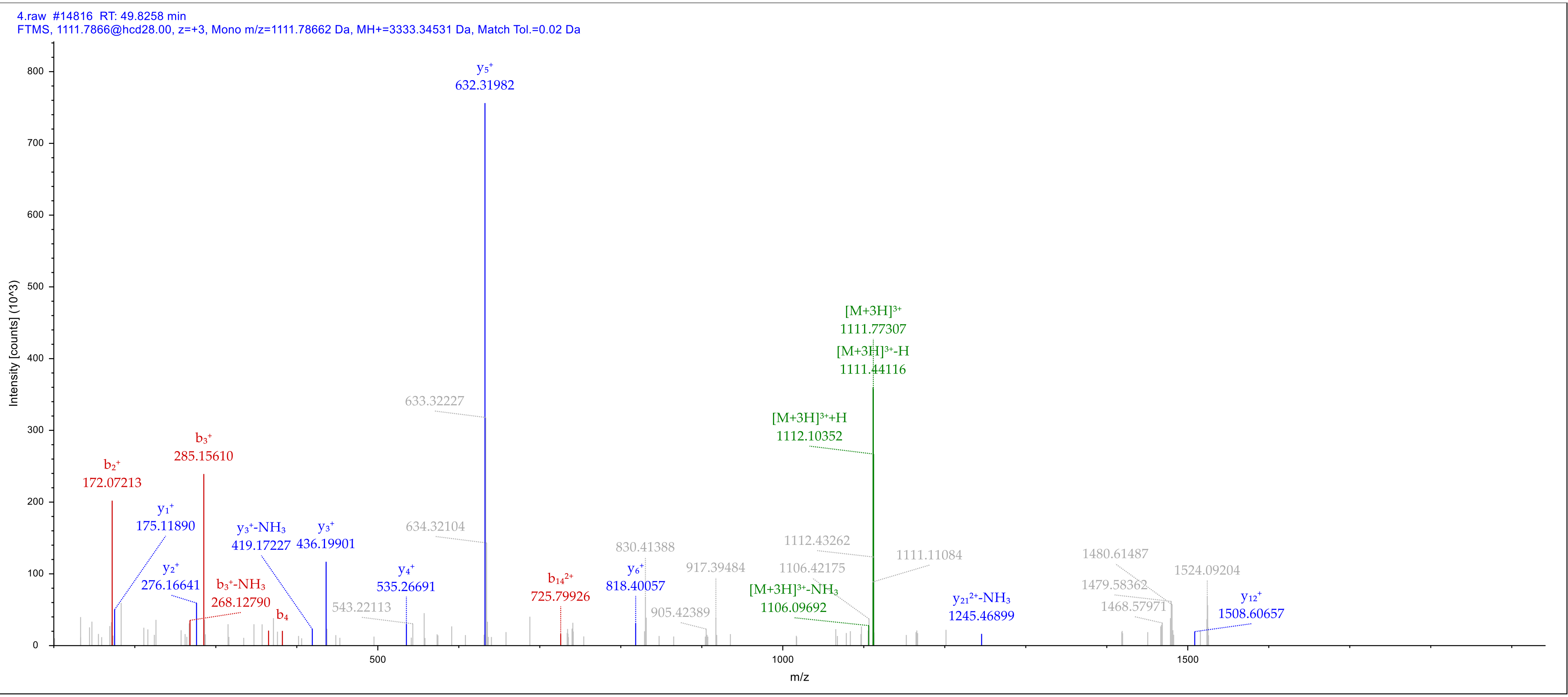

Viba22_NGIPCGESCVFIPCISSVIGCSCK

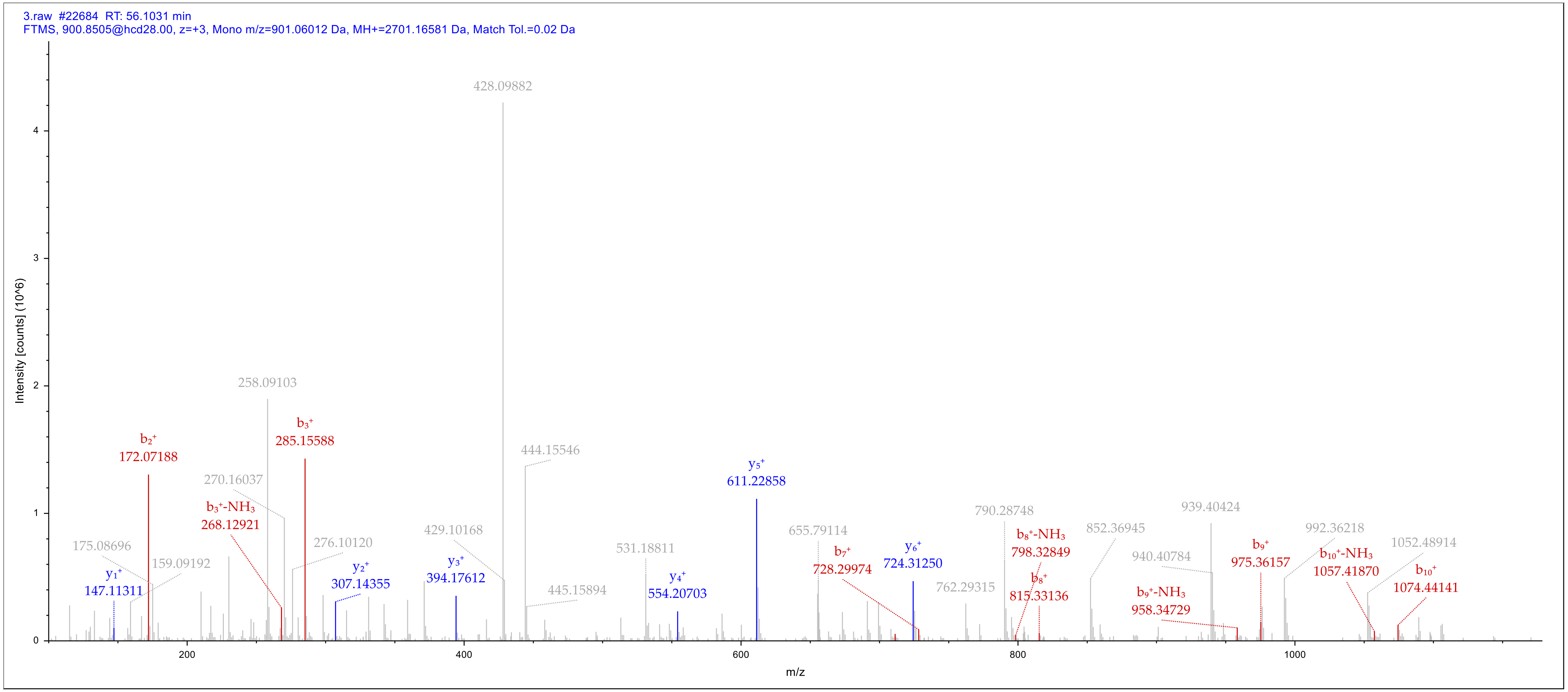

Viba24_IPCGESCVWIPCITTVVGCSCSNK

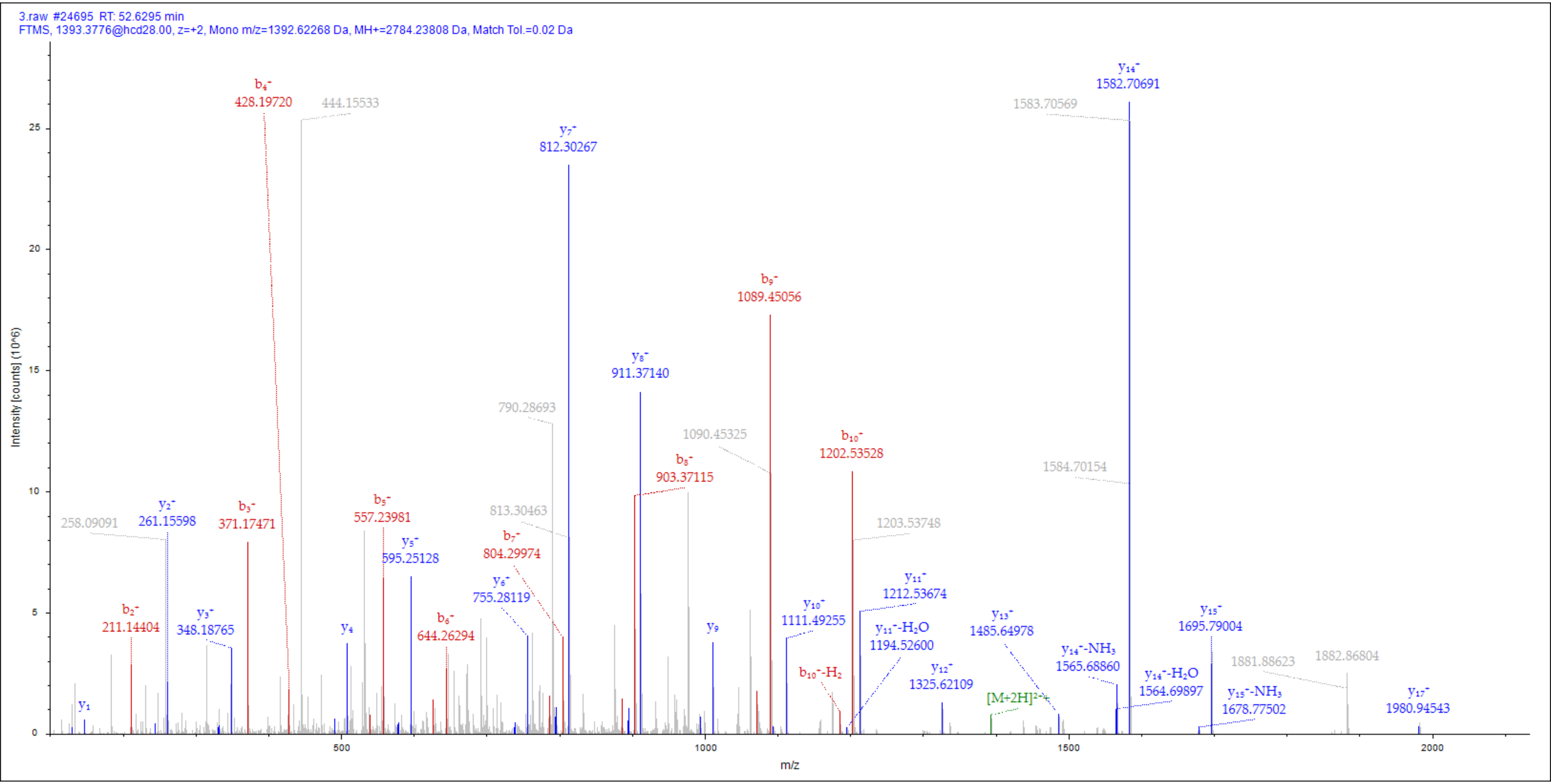

Viba25_VPCGESCVYIPCFTSIAGCSCSDK

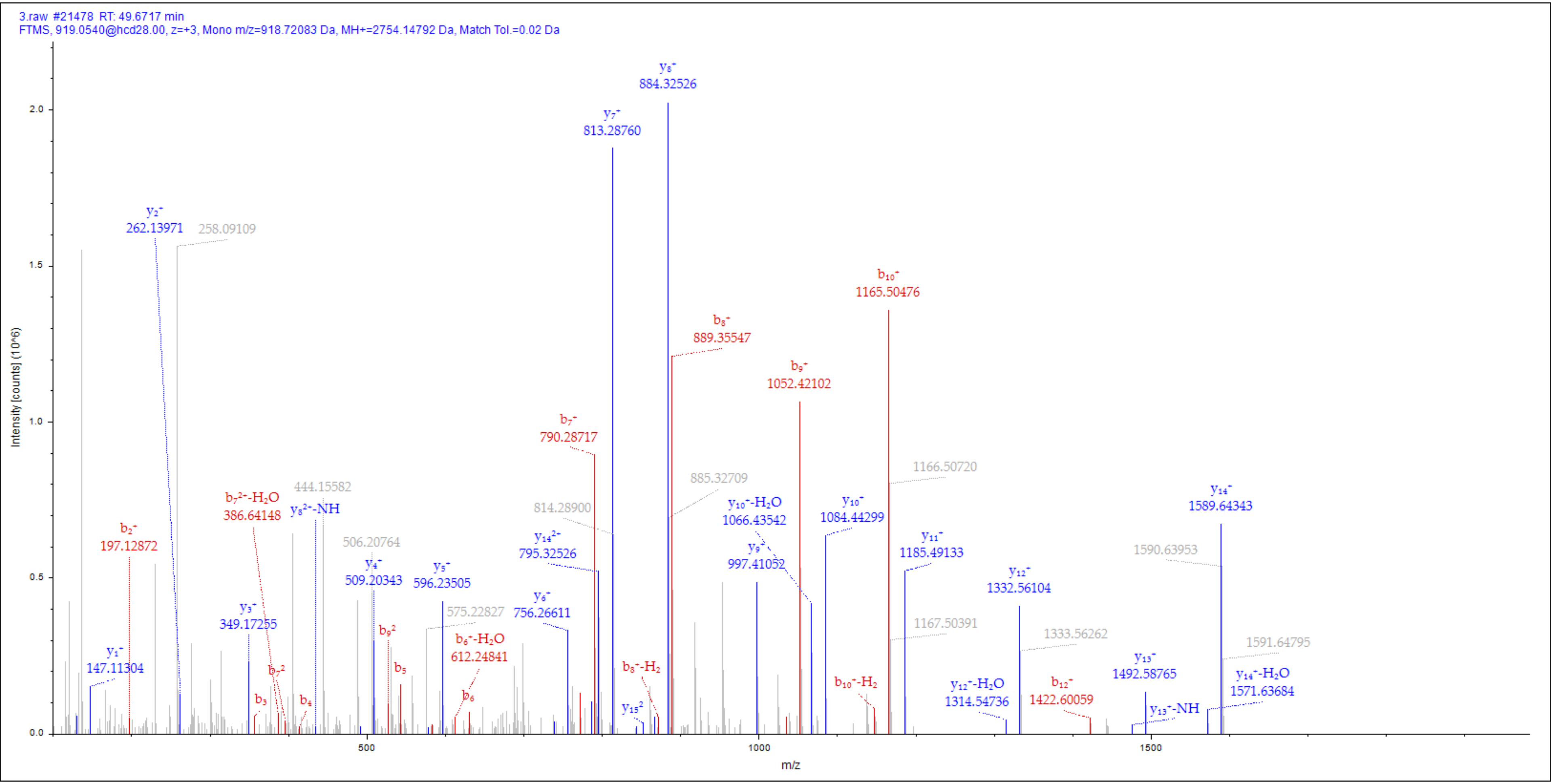

Viba29_NGLPVCGETCVGGTCSTPGCGCSWPVCTR

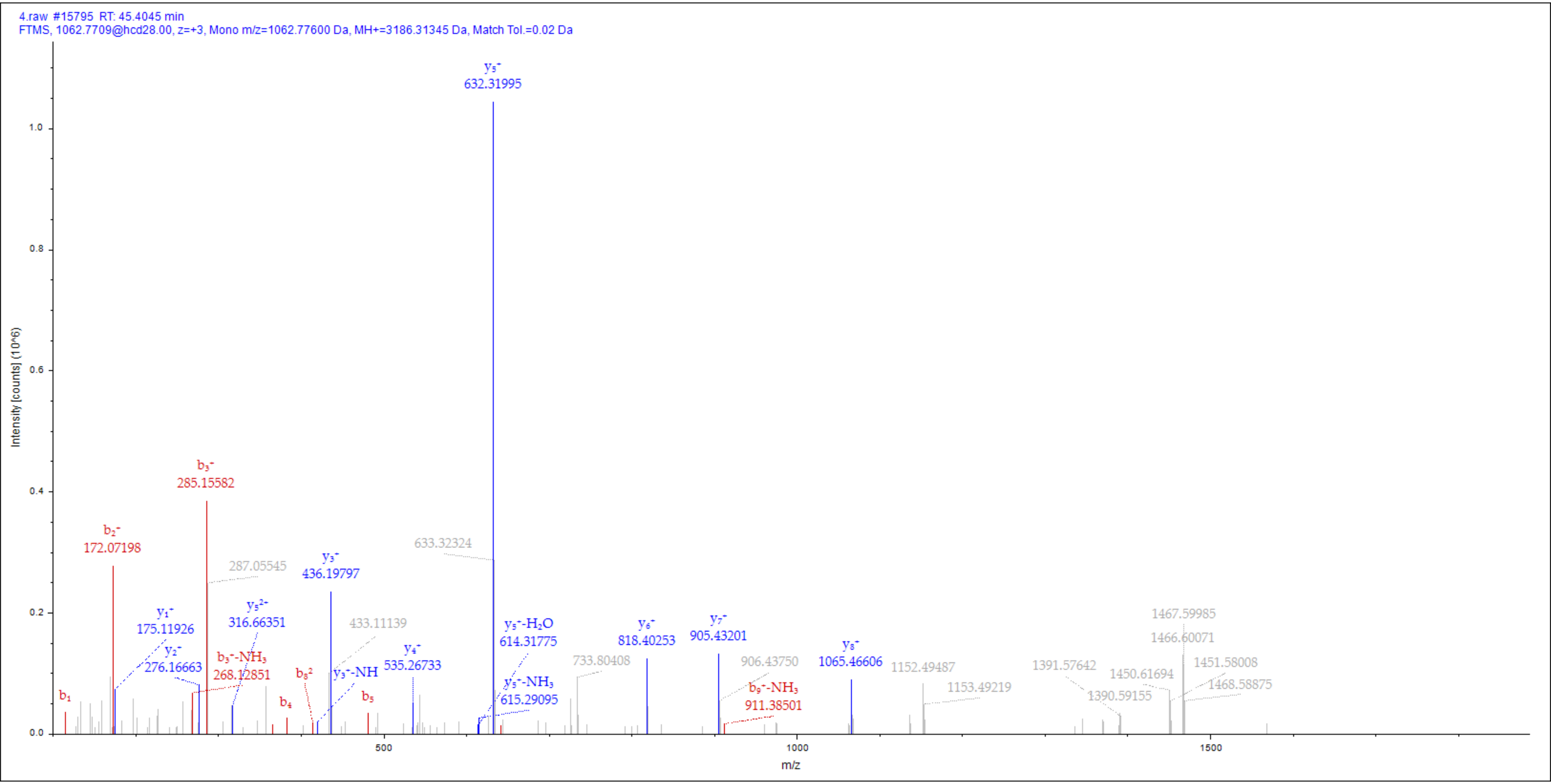

Viba30_NGPPVCGETCVGGTCNTPGCSCSWPVCTR

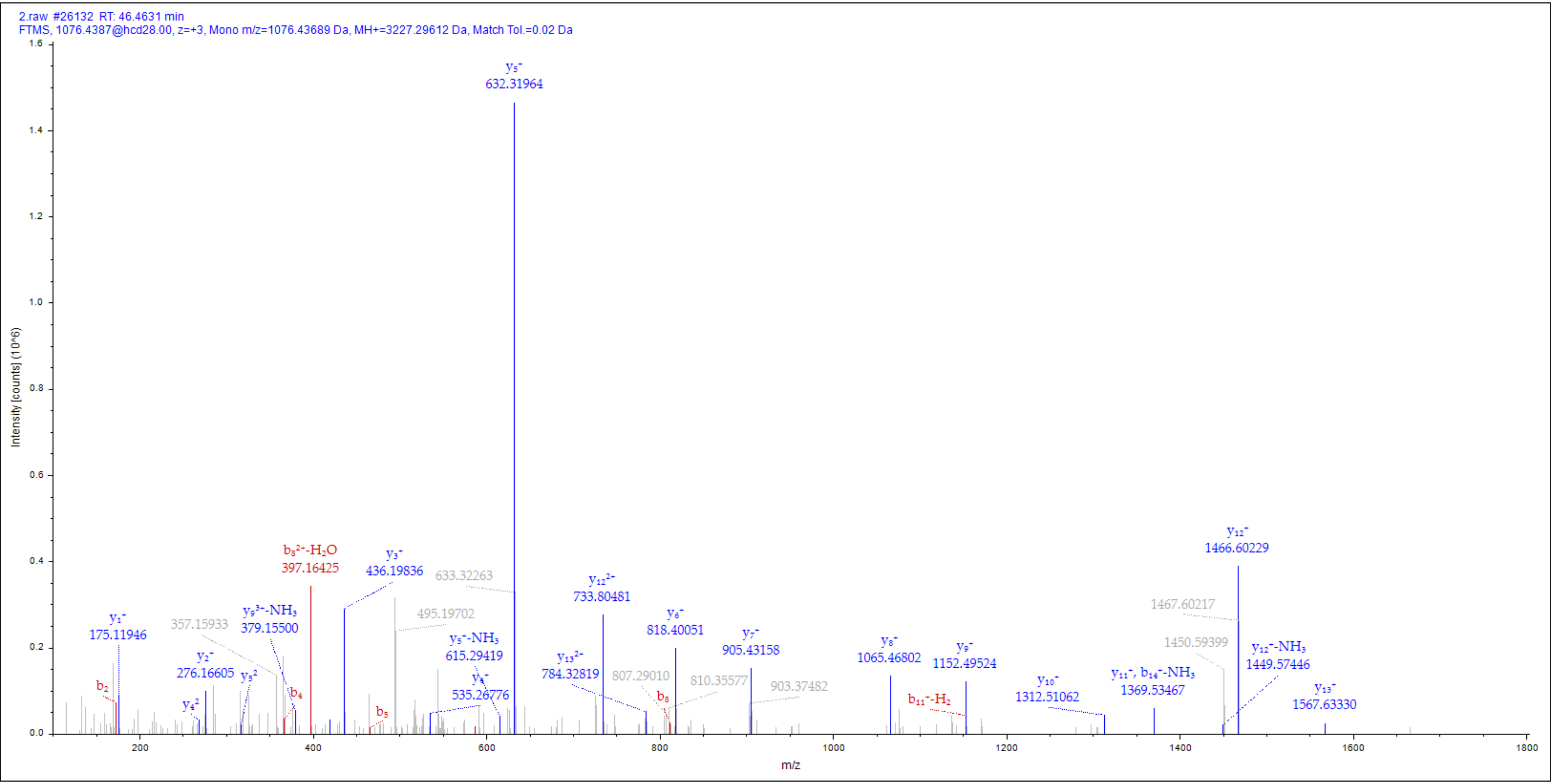

Viba31_NGLPVCGETCVGGACNTPGCACSWPVCTR

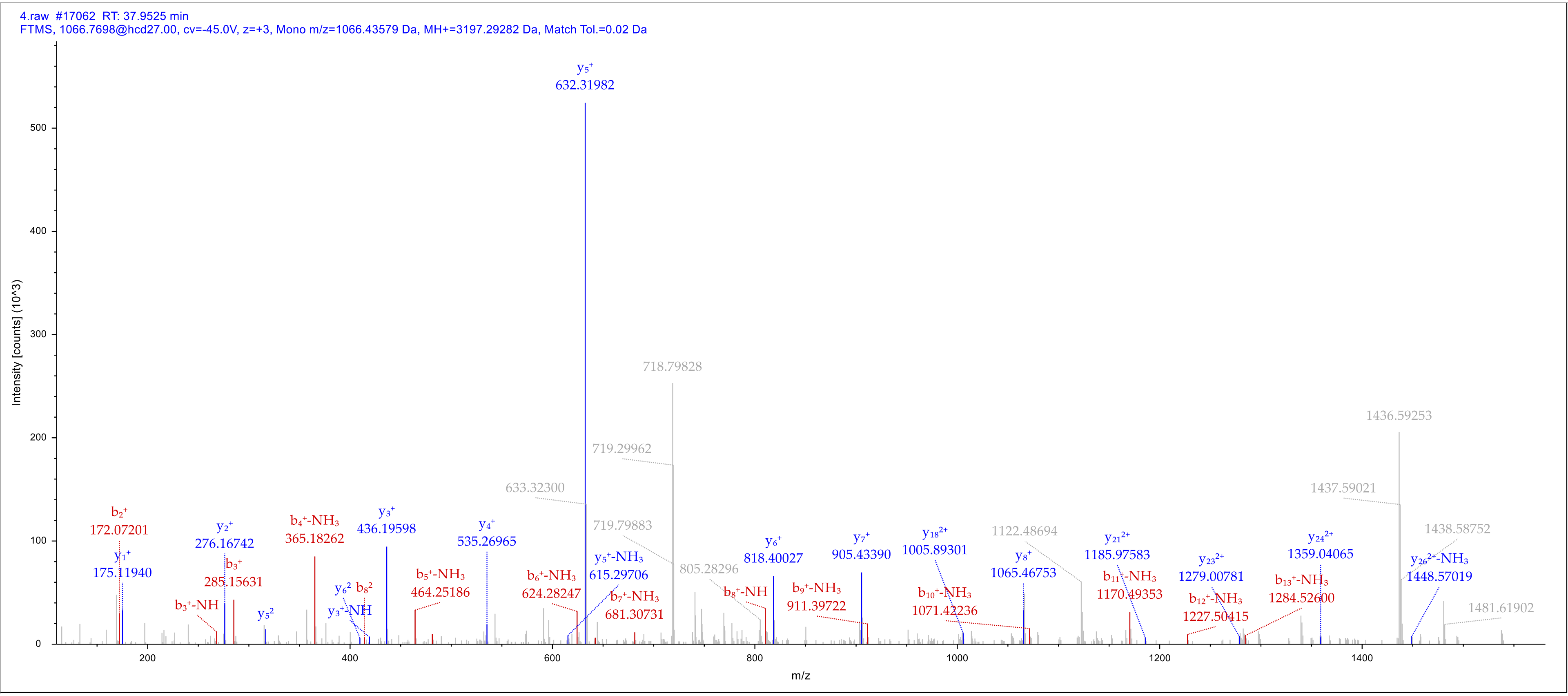

Viba33_NGLPVCGETCFGGTCNTPGCPCEWPVCTR

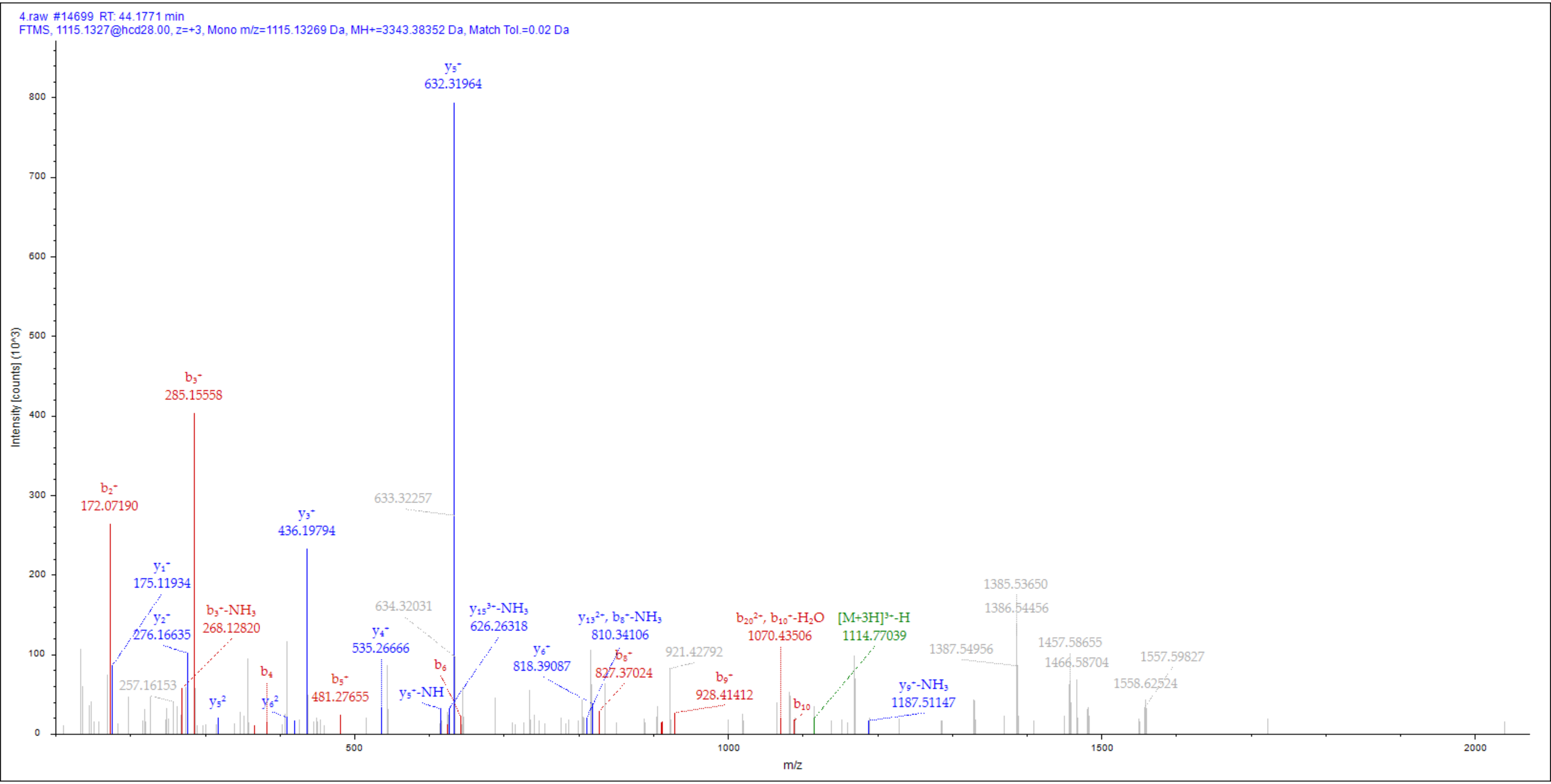

vibe_1_NGIPCGESCVWIPCITSAIGCSCSSK

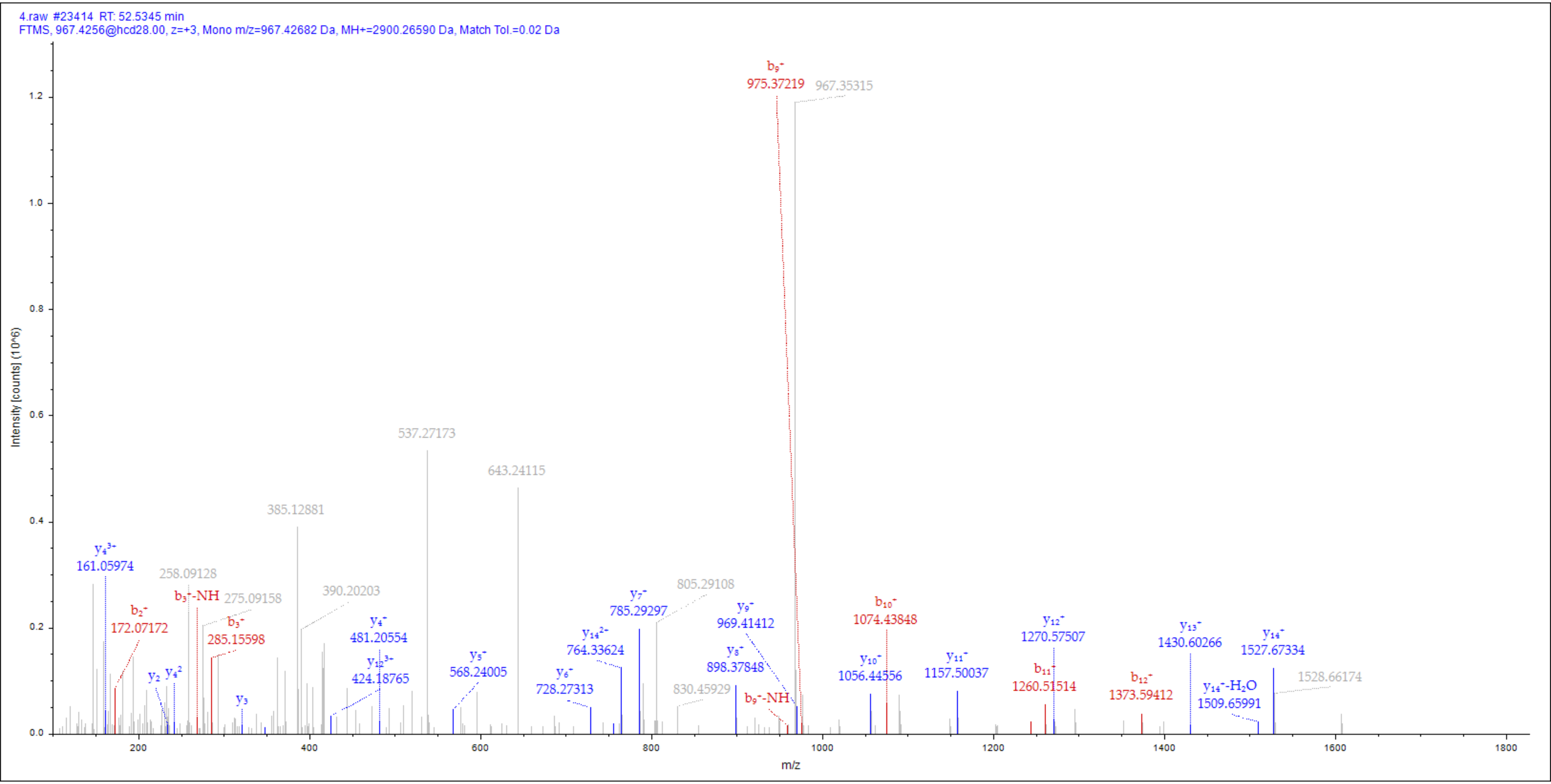

vibe_13_NGLPVCGETCVGGSCYTPGCTCSWPVCTR

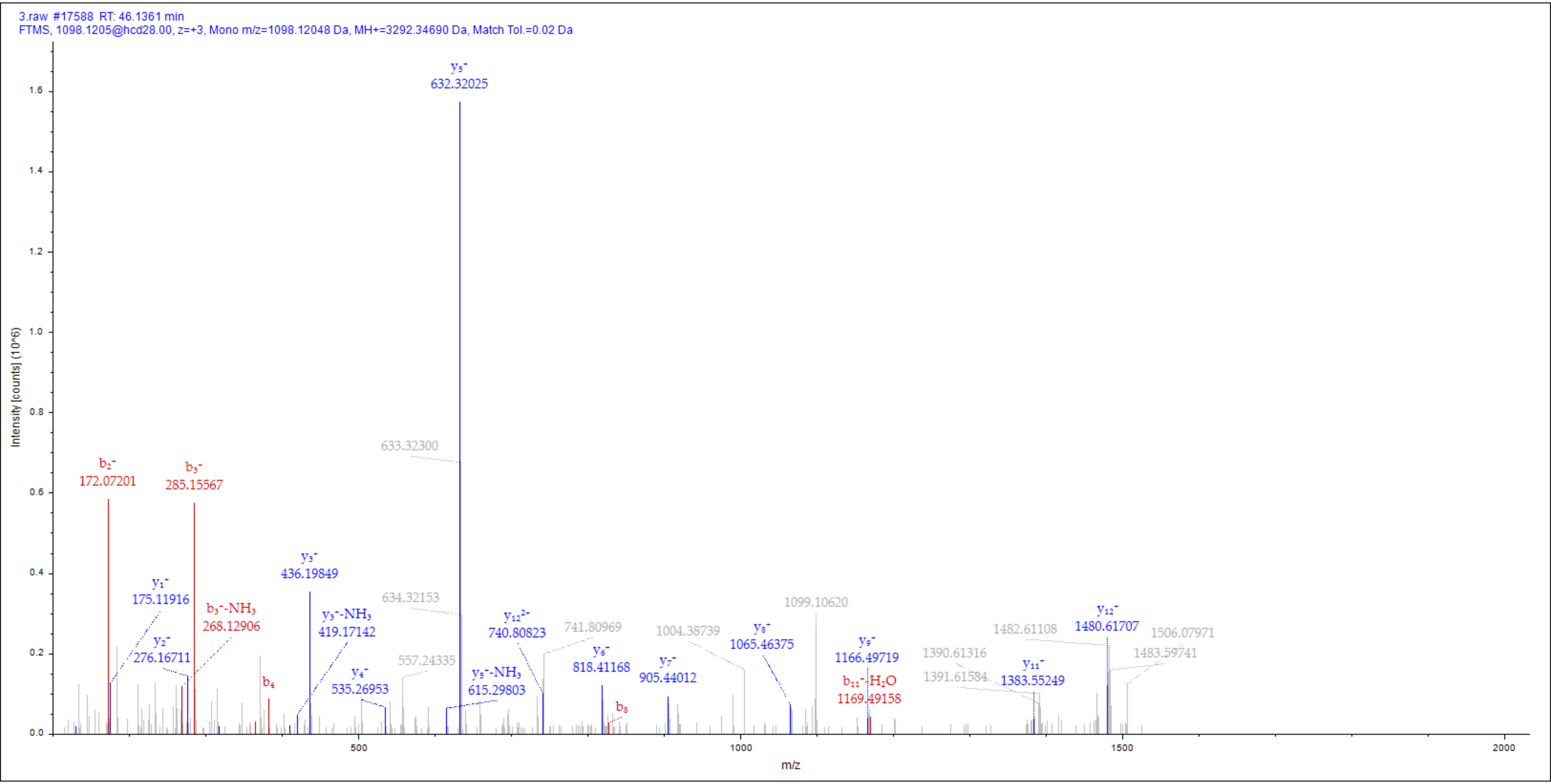

vibe_18_DGSIFNCGETCVFGTCYTPGCSCVYGACSK

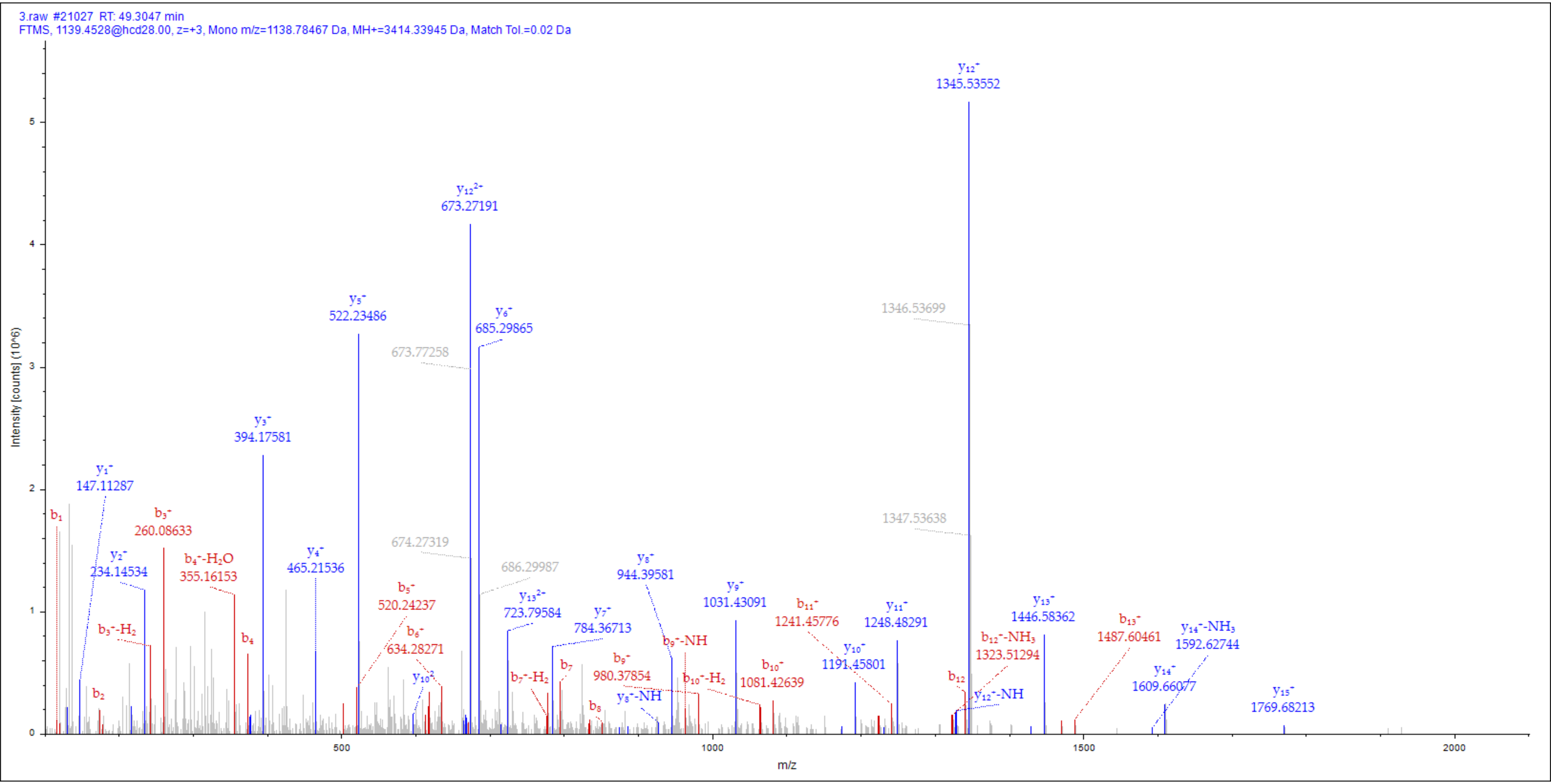

vibe_3_NGTFPCGESCVFIPCISSVVGCSCK

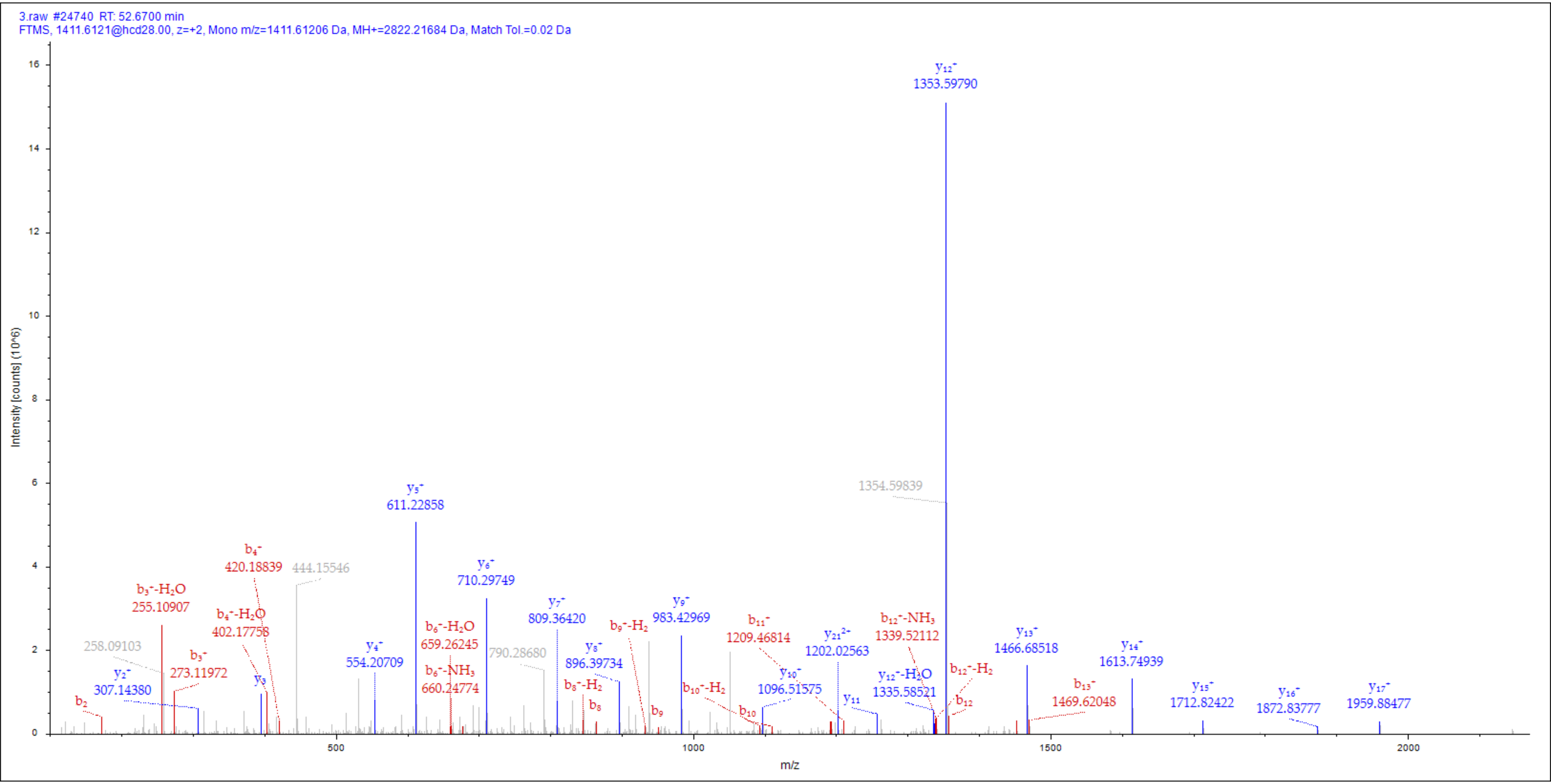

vibe_7_AAVPCGESCVWIPCVTSVVGCSCSNK

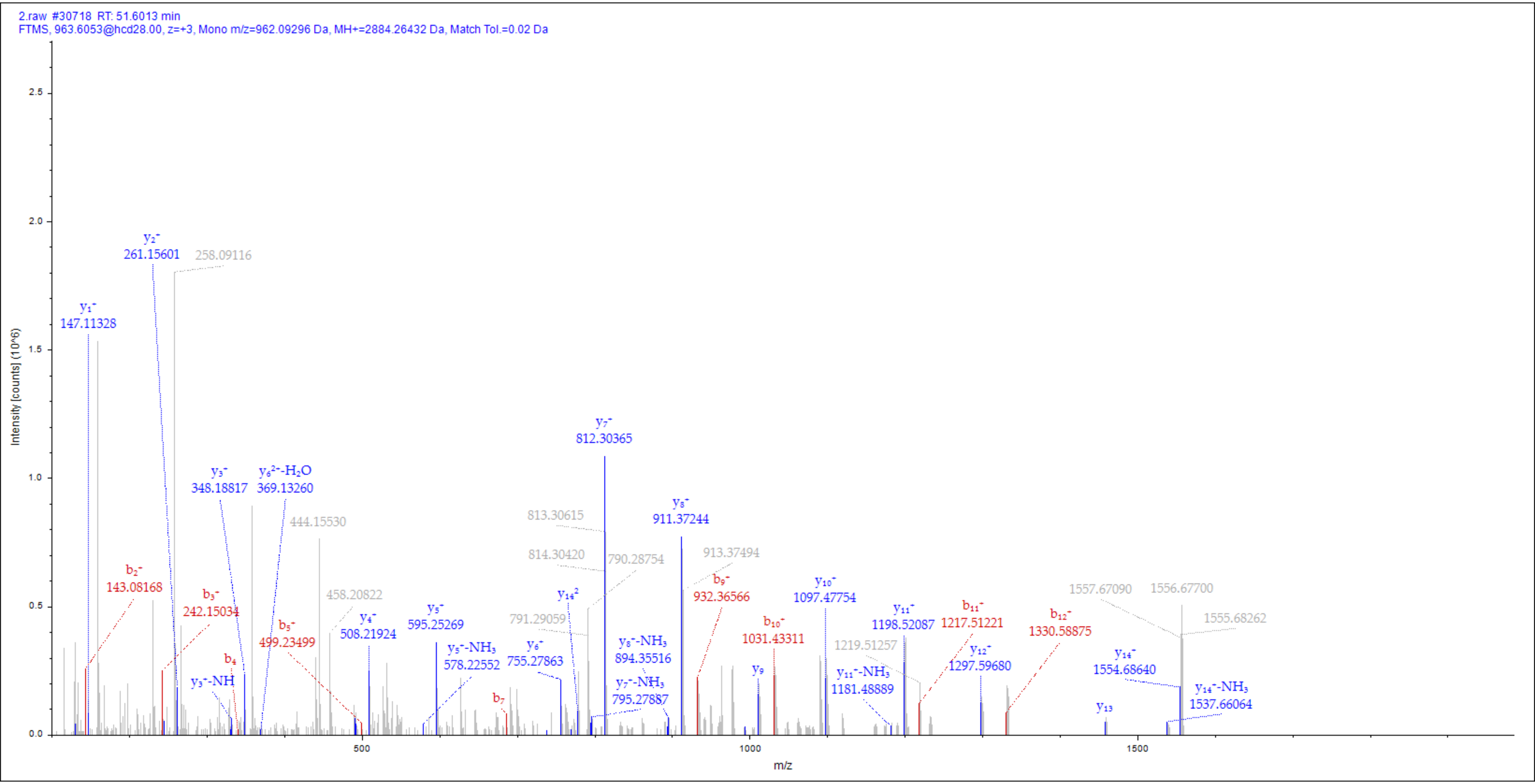

vibi_A_NGLPVCGETCFGGTCNTPGCSCSYPICTR

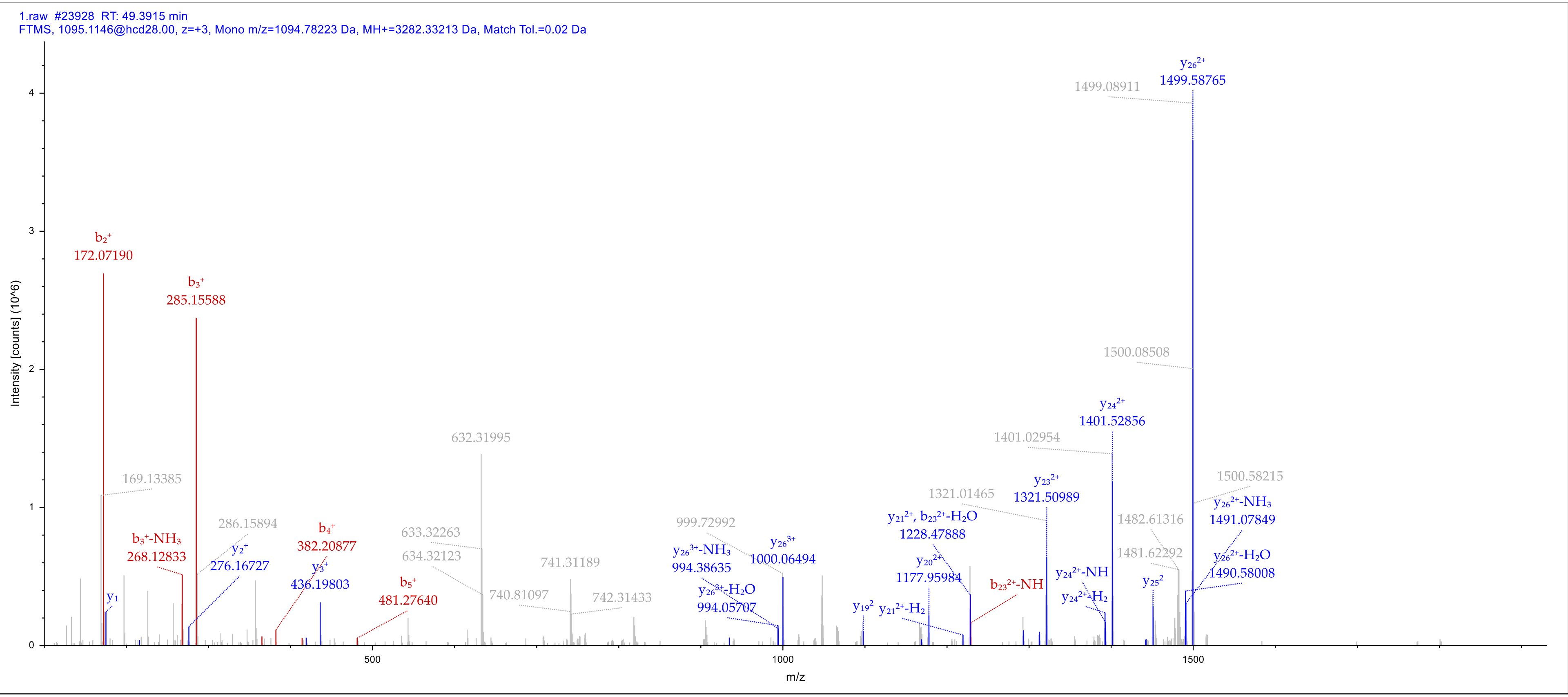

vibi_B_NGLPVCGETCFGGTCNTPGCTCSYPICTR

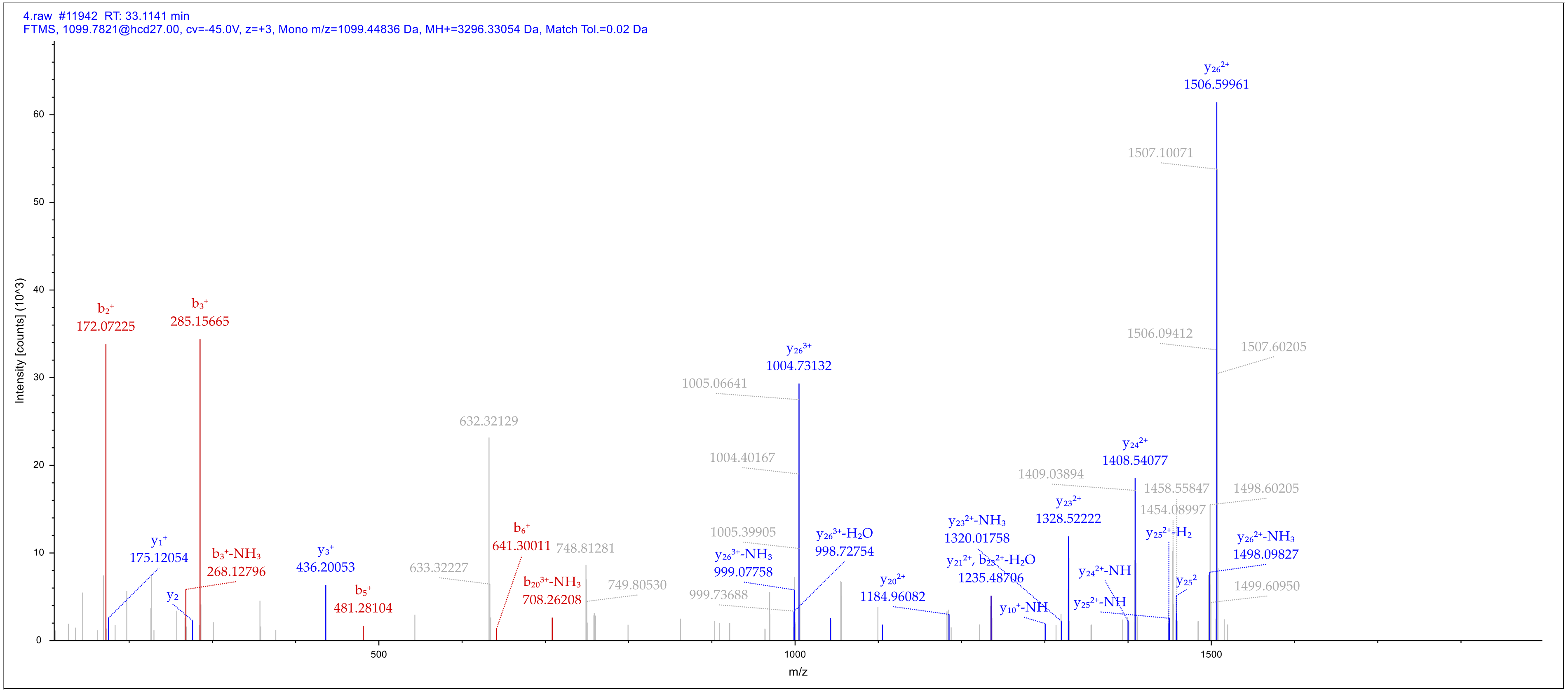

vibi_C_NGLPVCGETCAFGSCYTPGCSCSWPVCTR

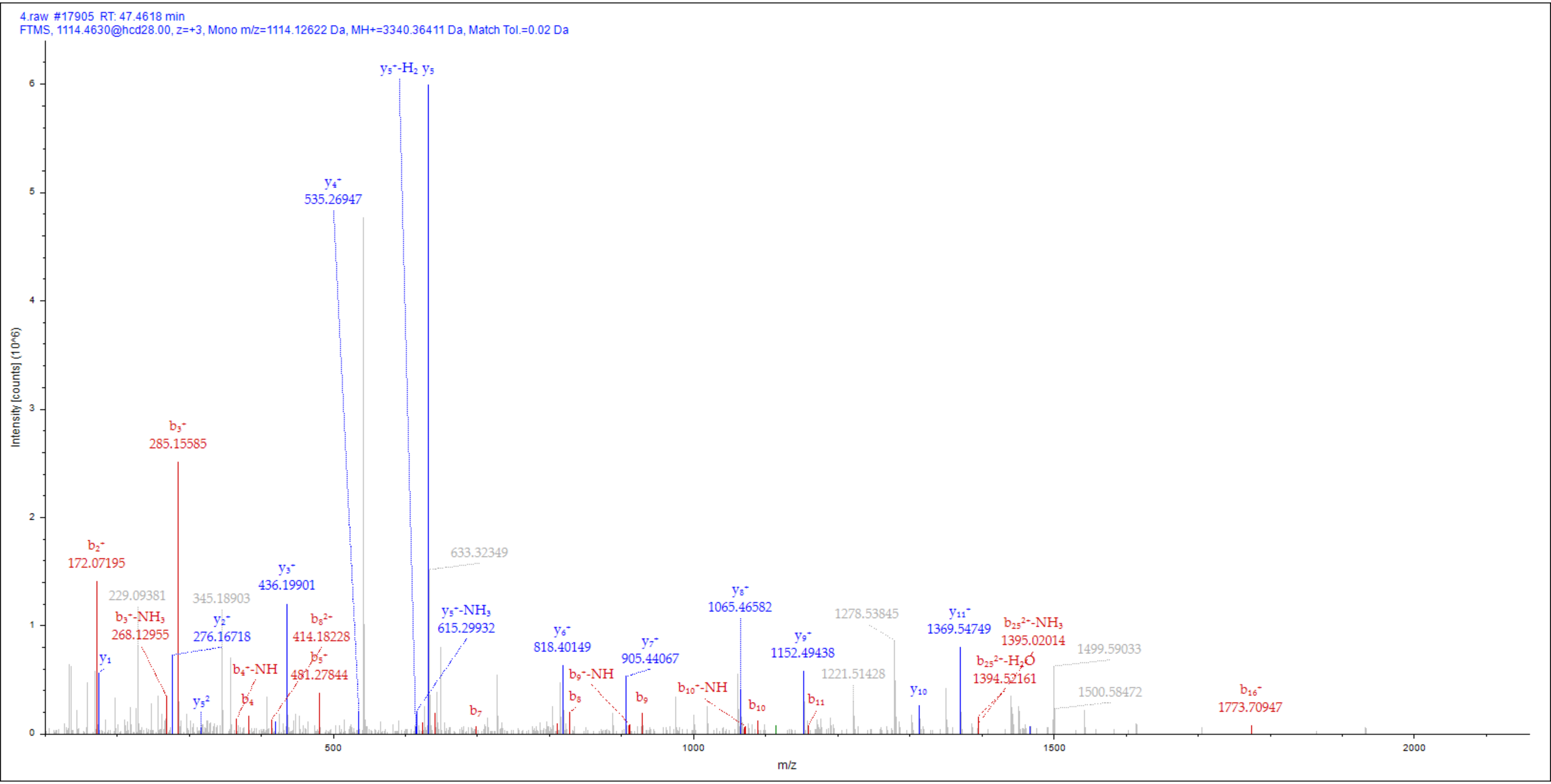

vibi_D_NGLPVCGETCFGGRCNTPGCTCSYPICTR

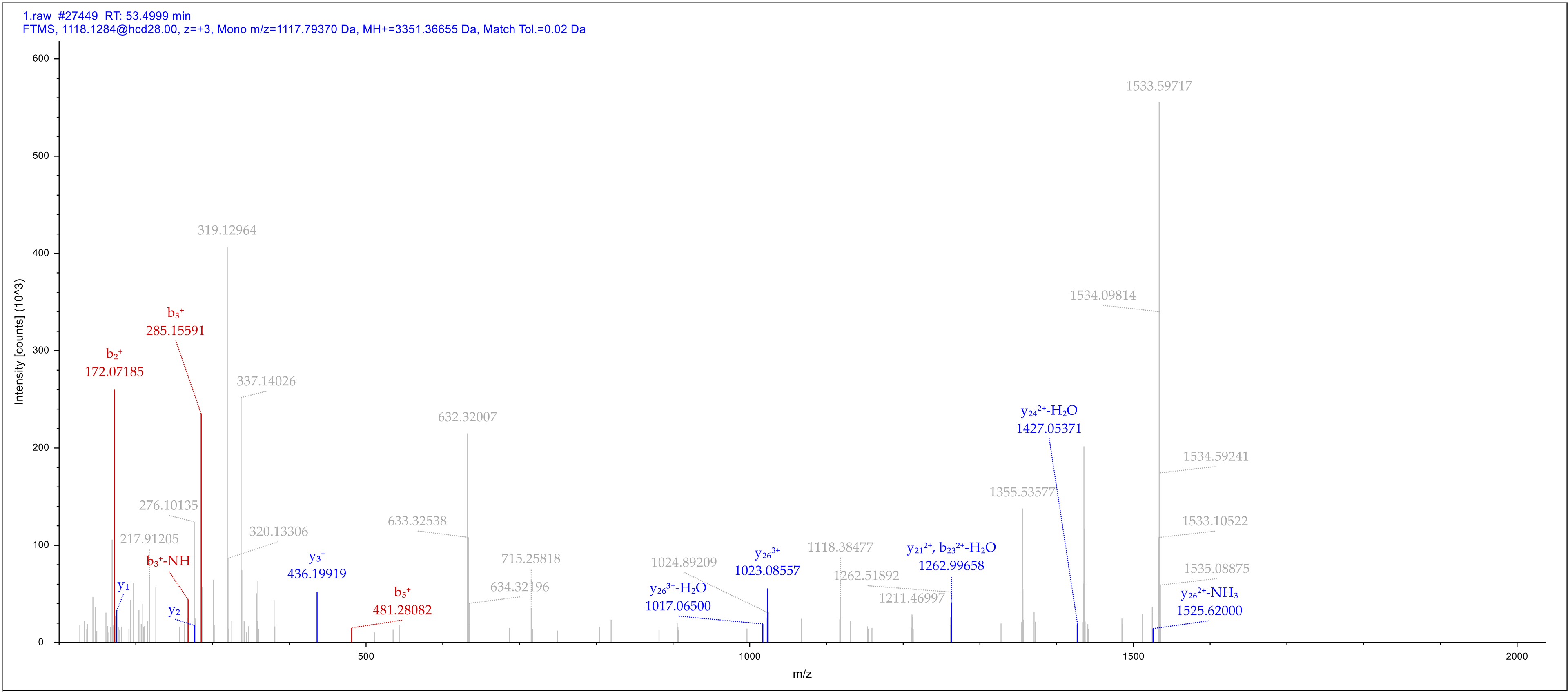

vigno_4_NGLPLCGETCVGGTCNTPACSCSWPVCTR

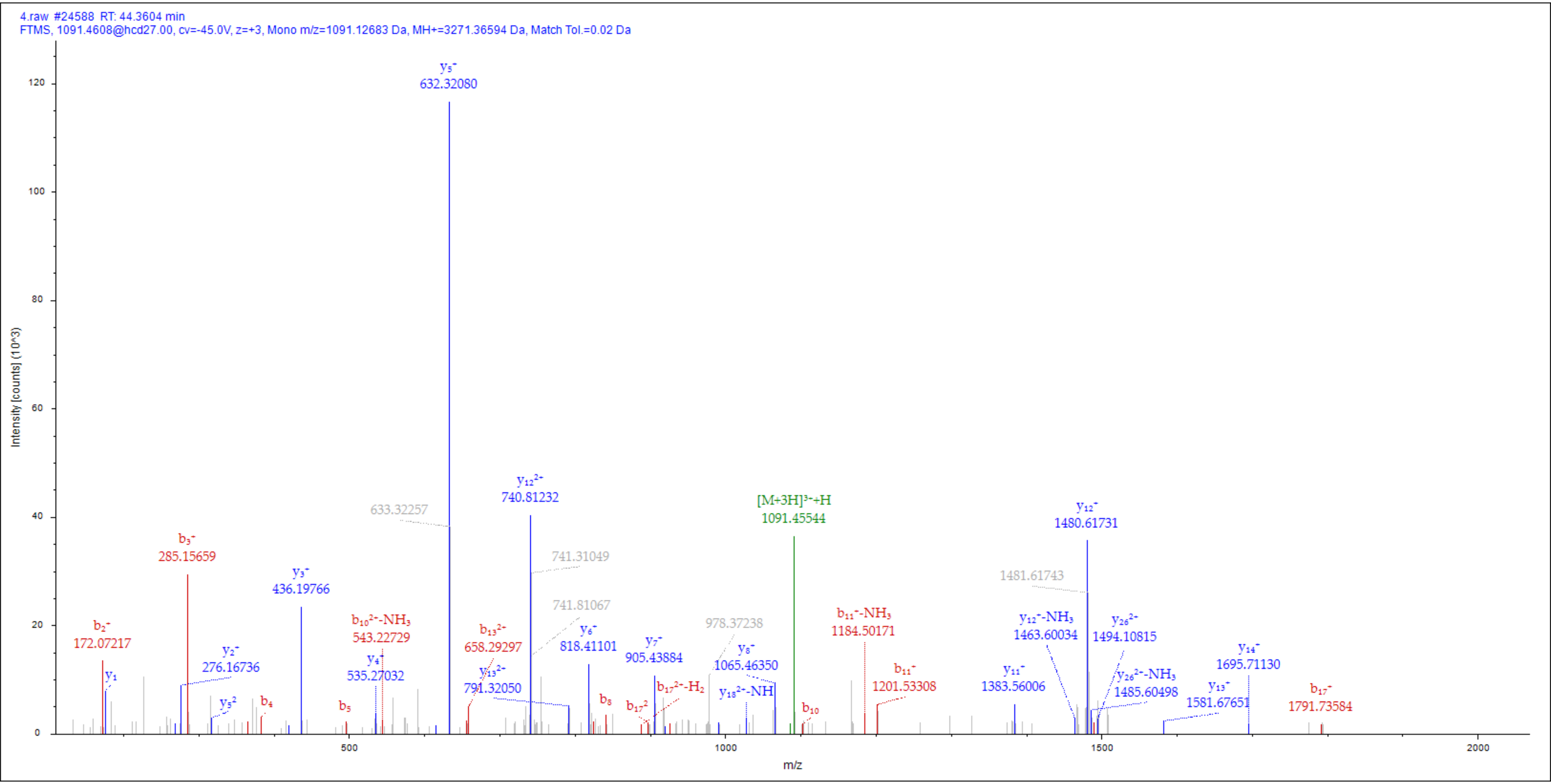

vigno_10_DGTIPCGESCVWIPCISSVVGCSCK

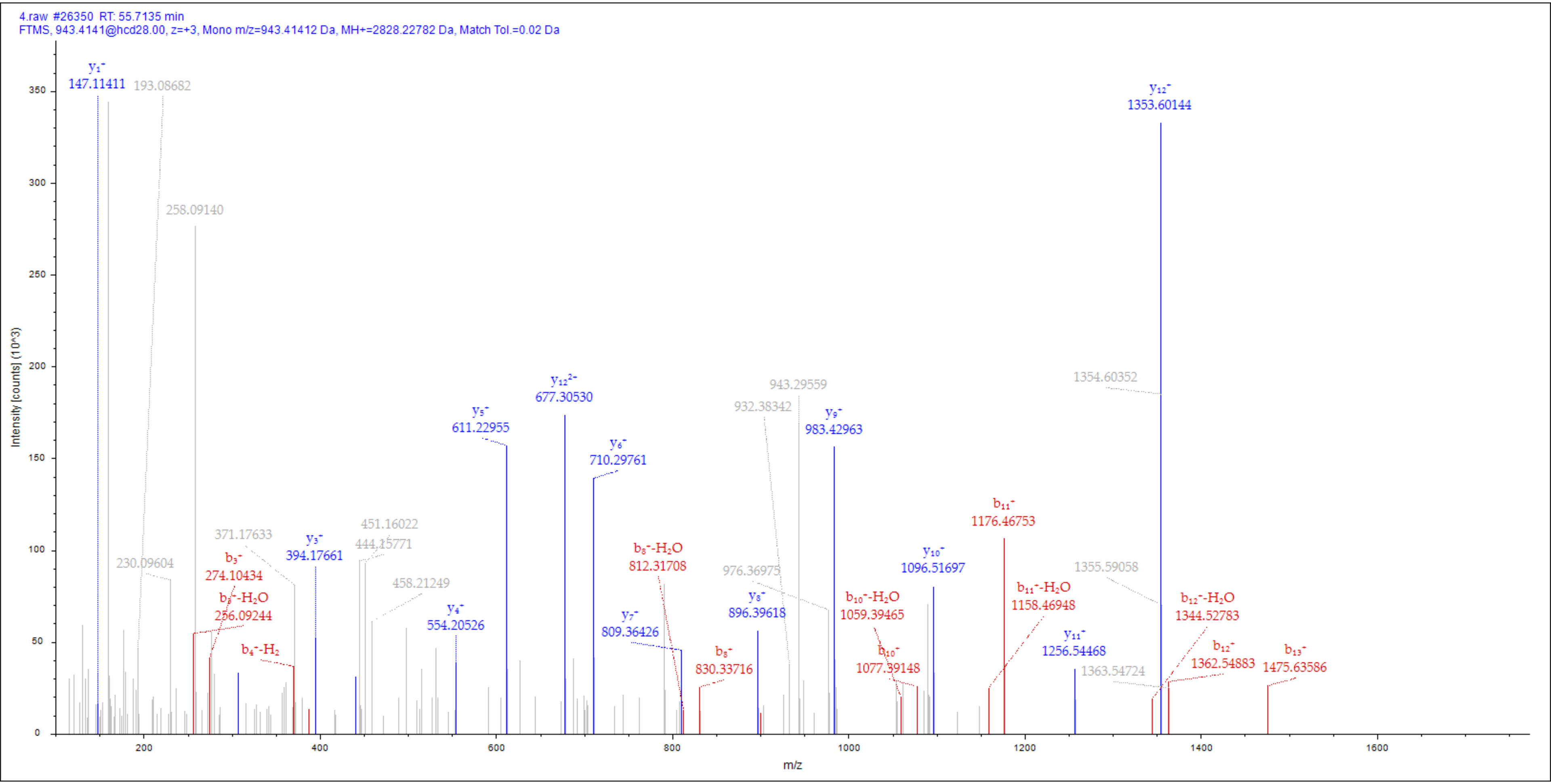

vigno_8_NGIPCGESCVWIPCITSAVGCSCK

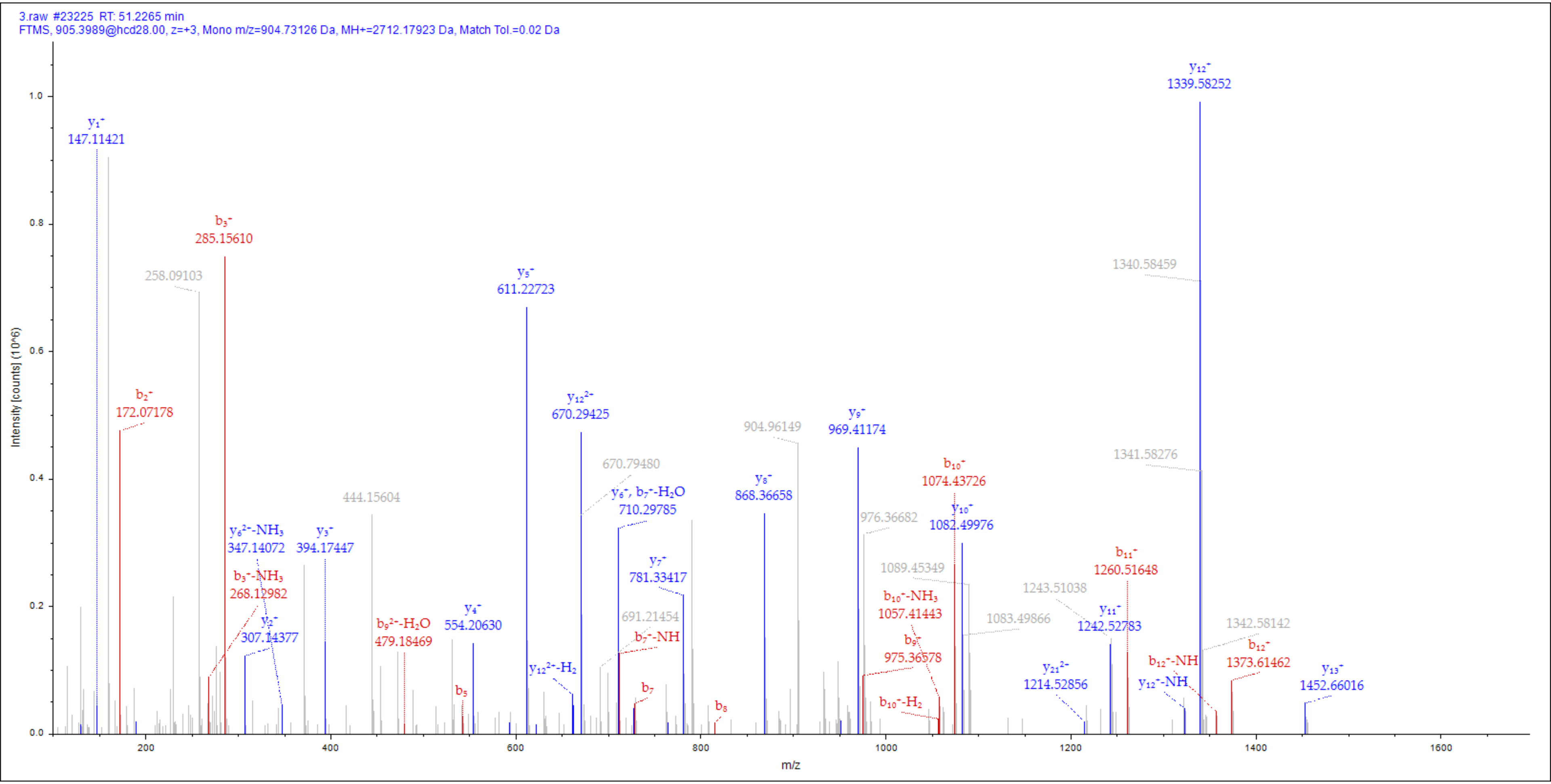

vila_A_DGIPCGESCVWIPCISSAIGCSCK

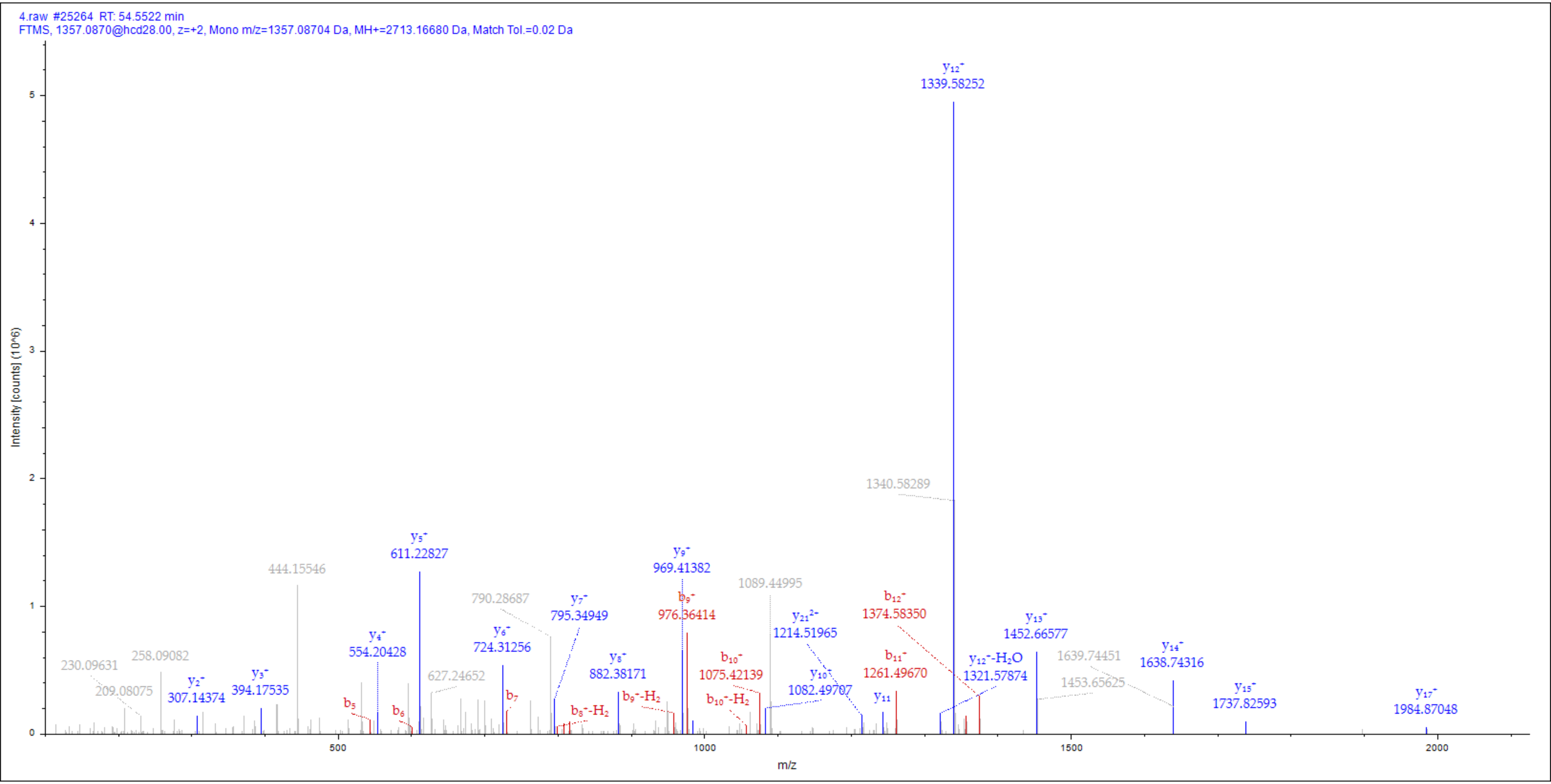

vila_D_NGIPVCGETCAFGTCYTGGCSCSWPVCTR

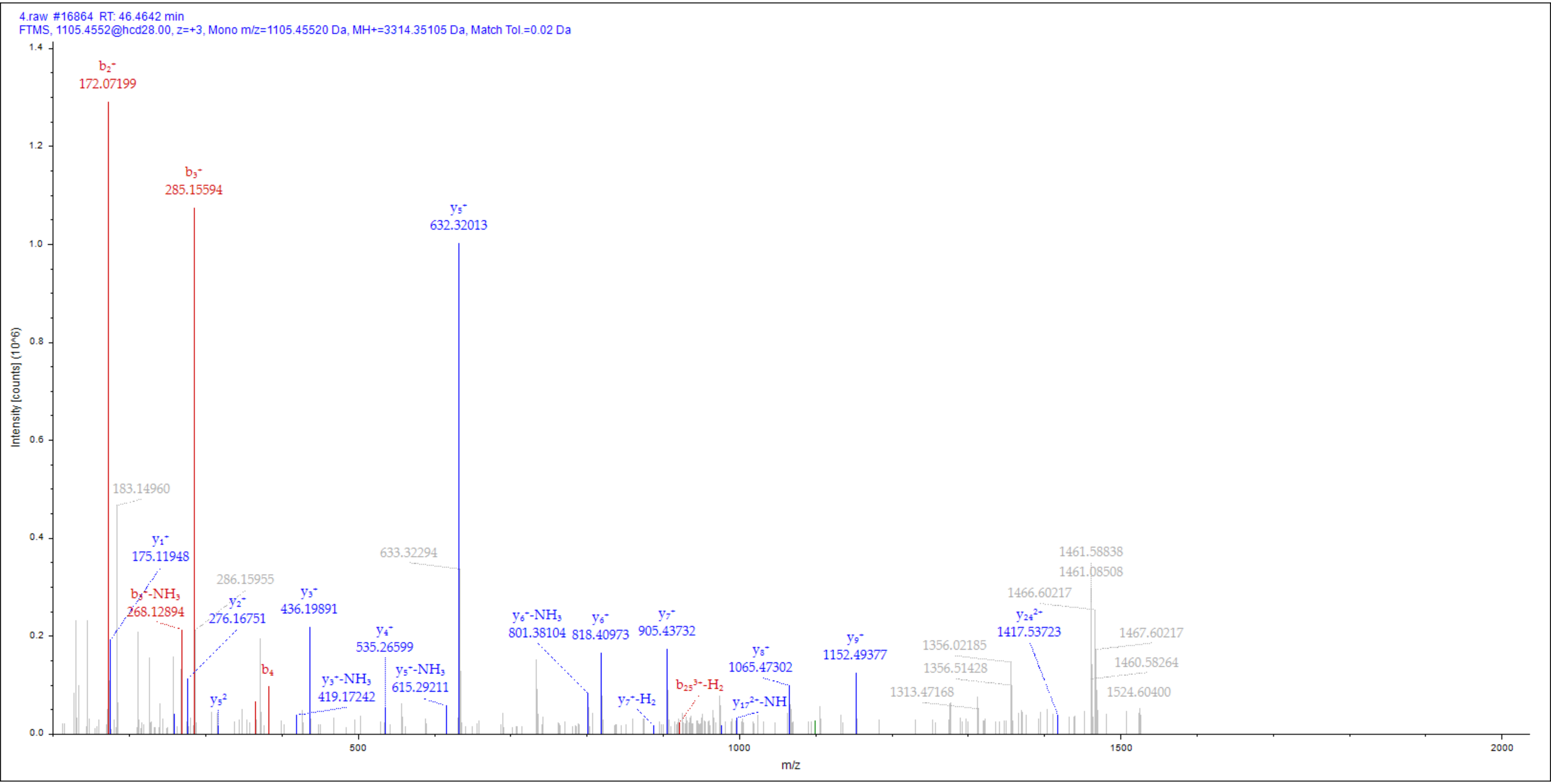

Vinc_A_NGIPVCGETCTLGTCYTAGCSCSWPVCTR

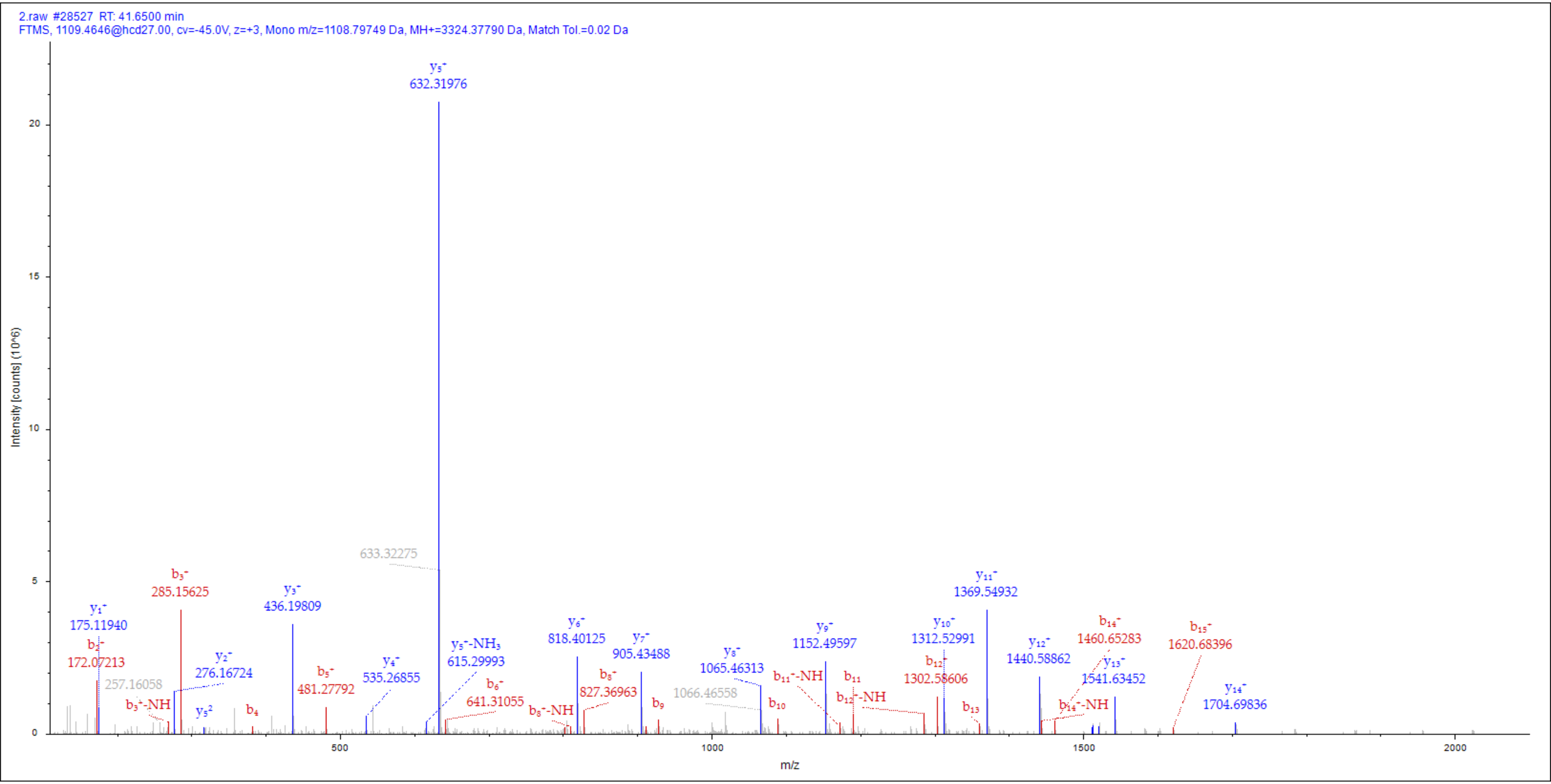

viphi_A_NGSIPCGESCVFIPCISSVIGCACK

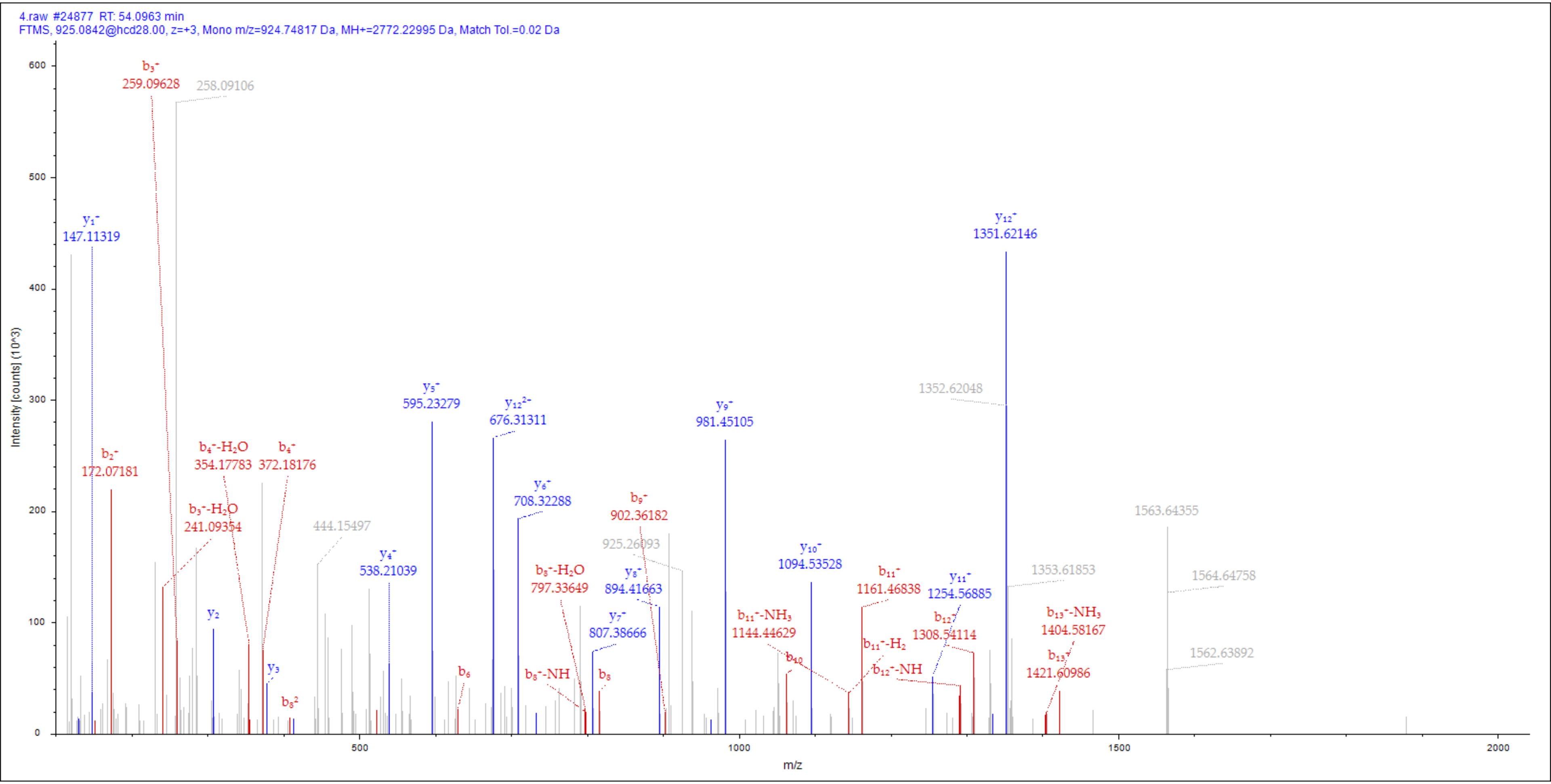

viphi_B_NGLPVCGETCTIGTCYTAGCTCSWPICTR

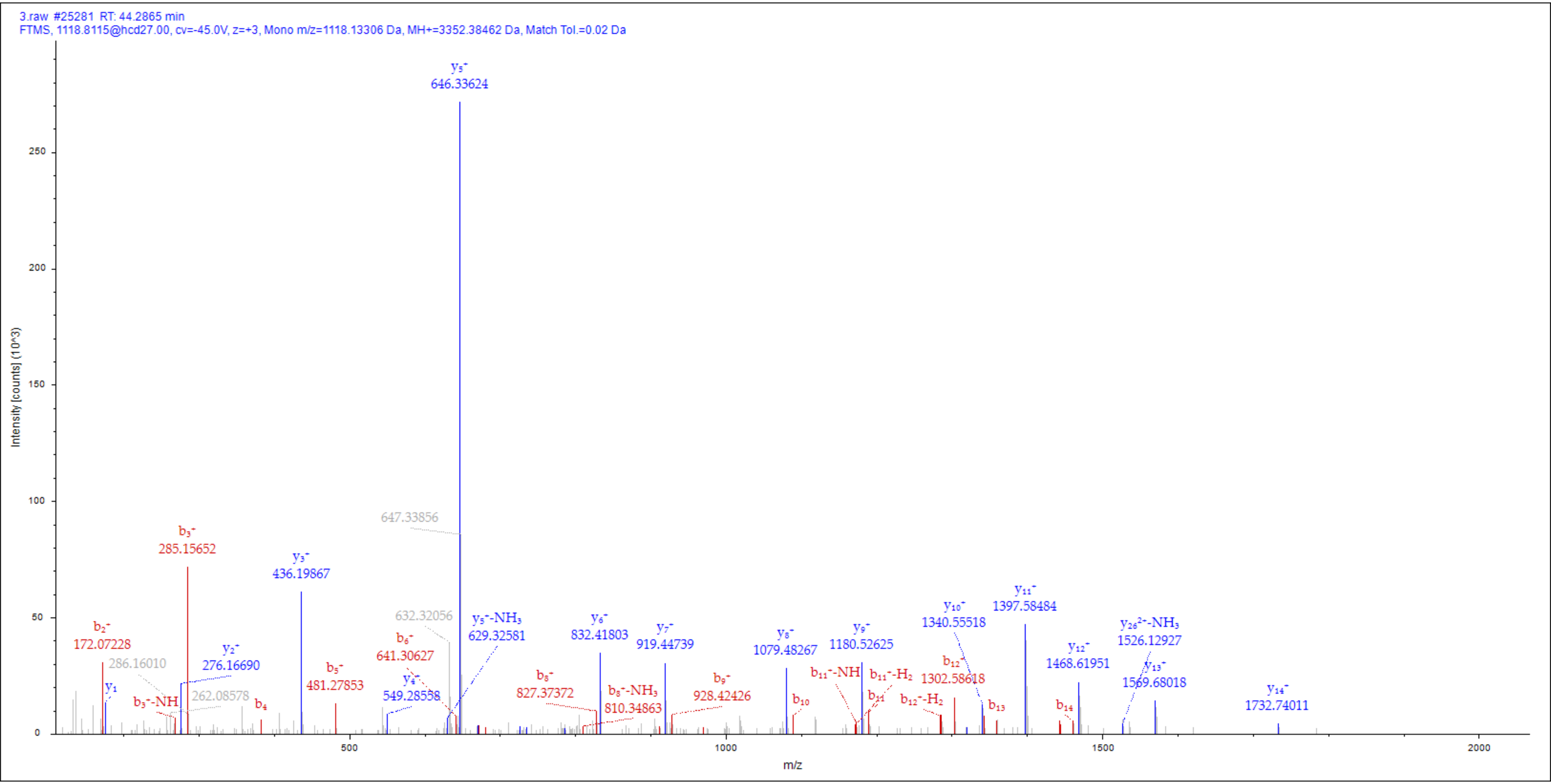

viphi_D_NGIPCGESCVFIPCISSVIGCSCSSK

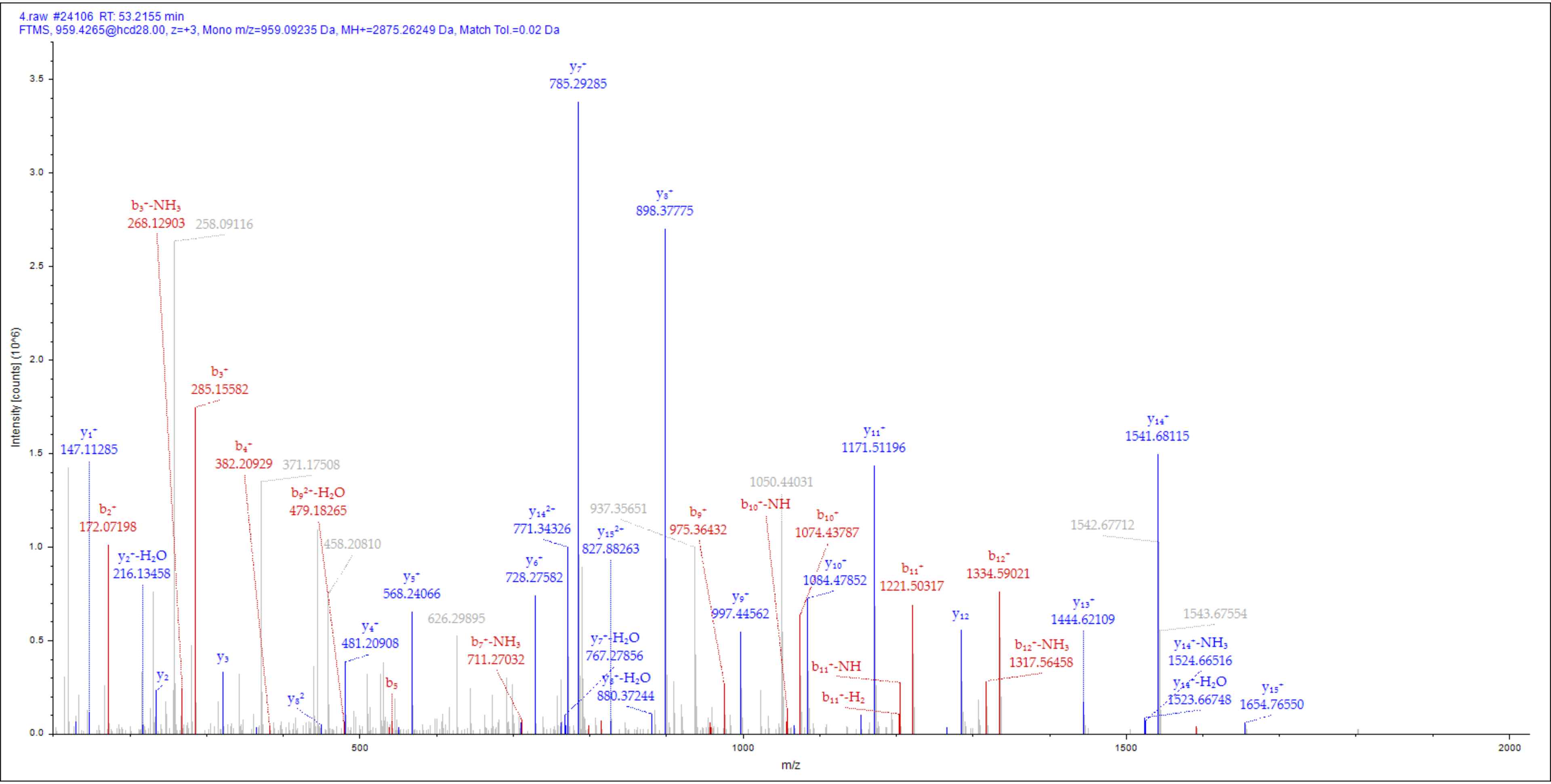

Visu_1_NGLPVCGETCVGGTCNTPGCTCTWPVCTR

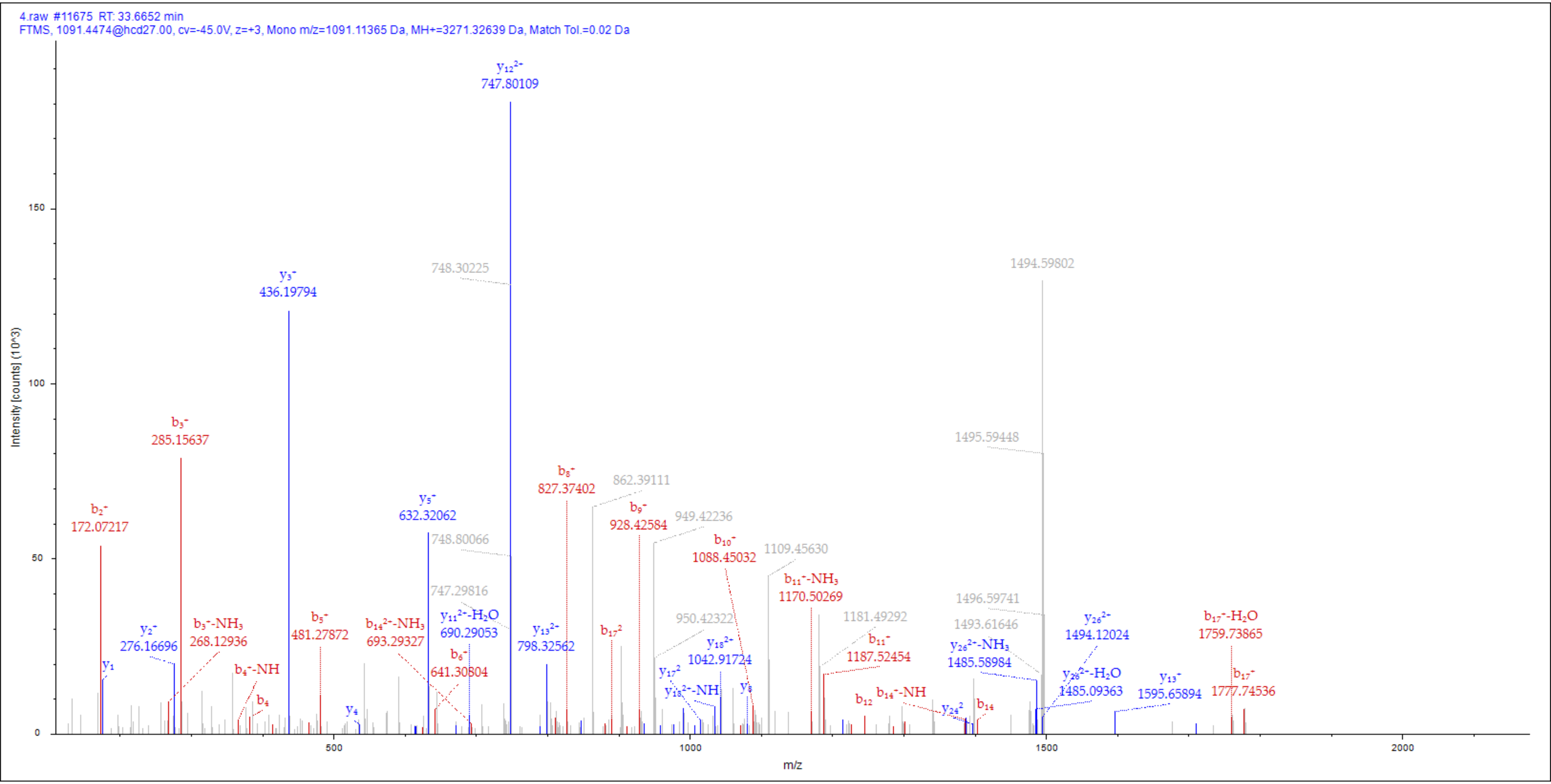

vitri_A_SKVCYRNGIPCGESCVWIPCITSAIGCSCK

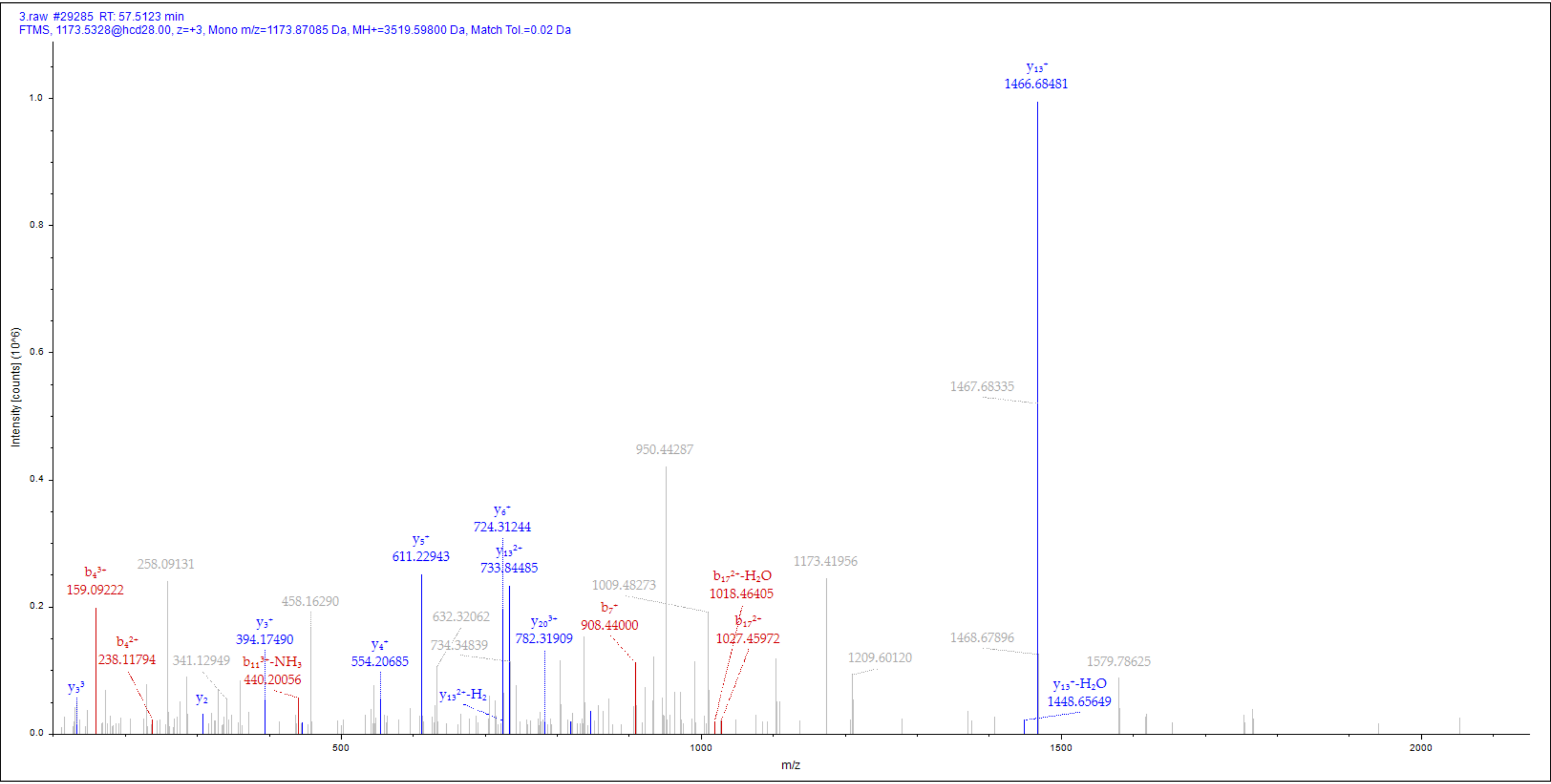

vitri_E_NGLPVCGETCVGGTCNTPGCSCSWPVCFR

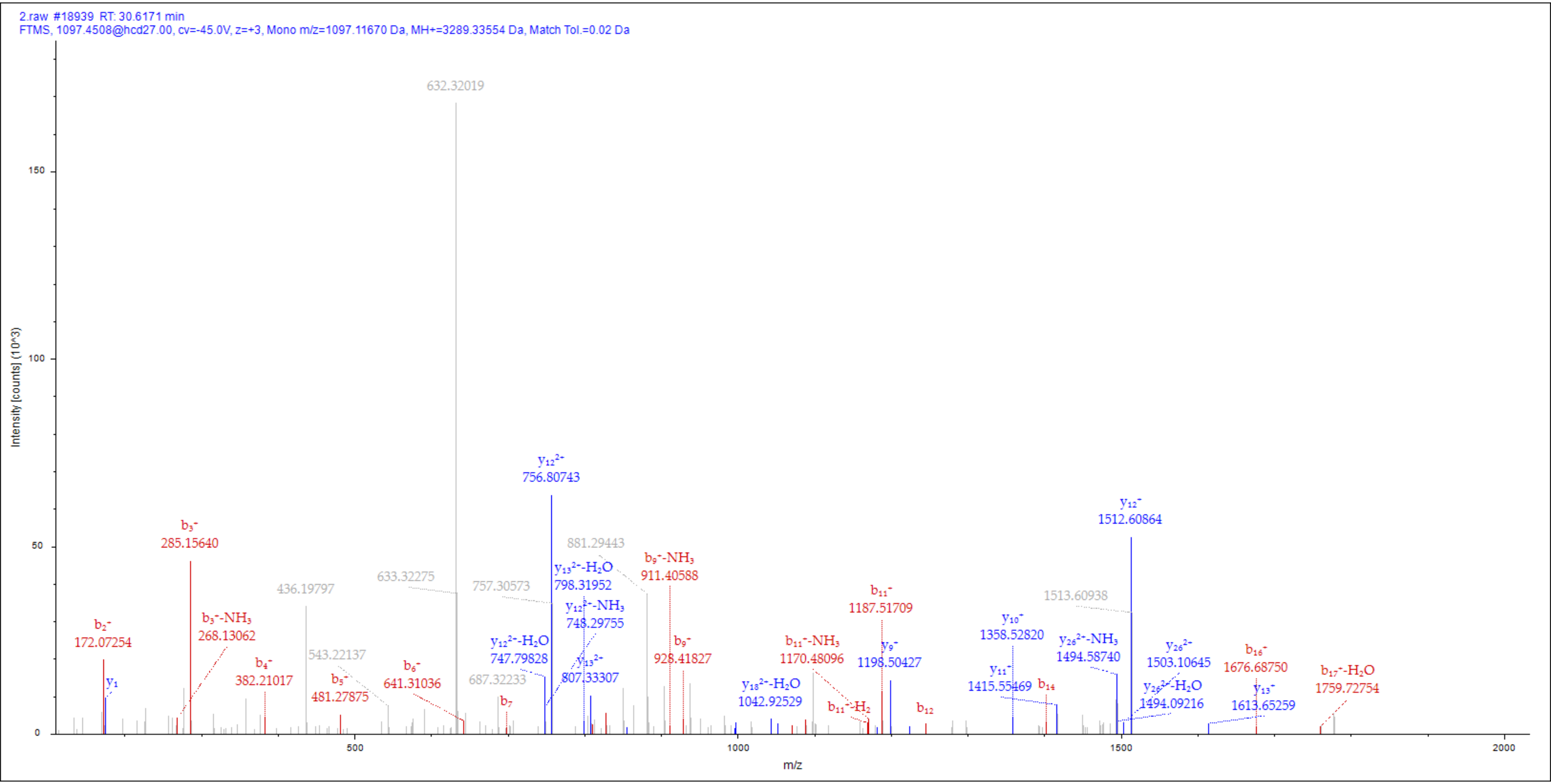

ViulD_VCYKNGIPCGESCVWIPCLTSAIGCSCK

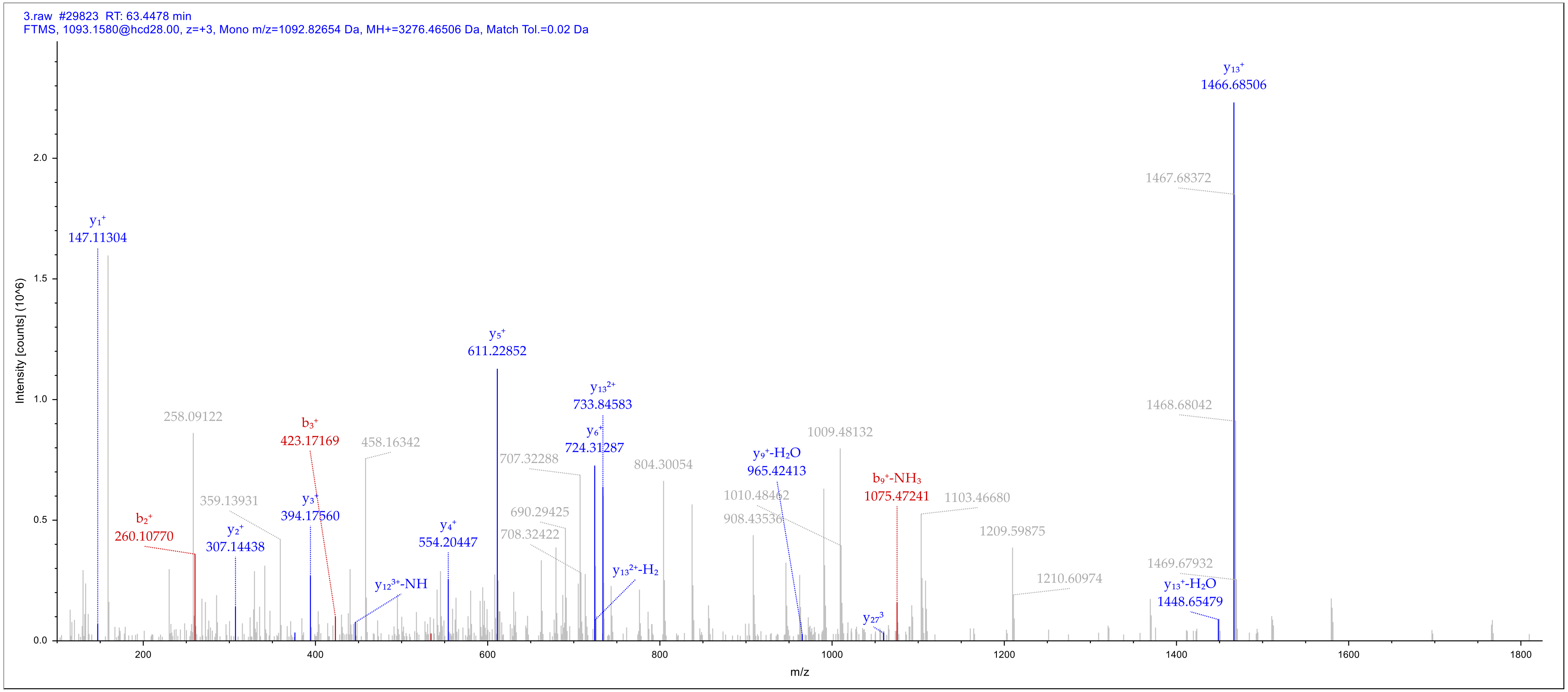

Vodo_L2_NALPVCGETCVGGTCNTPGCSCSWPVCTR

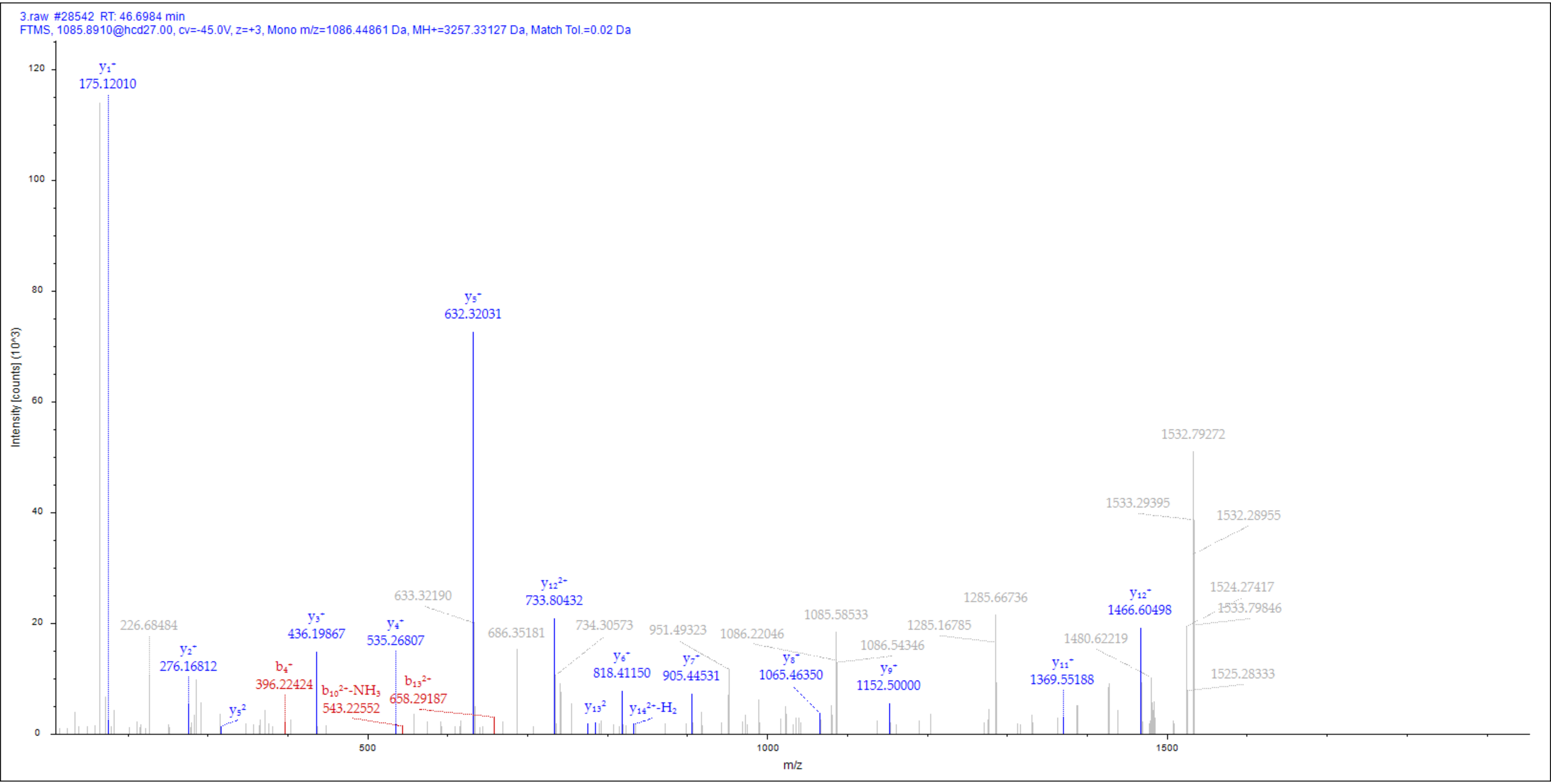

MS2 spectrum of putative novel cyclotides

Viphi I_VPCGDPSPTCVNTCNTPGCSCSWPVCTR

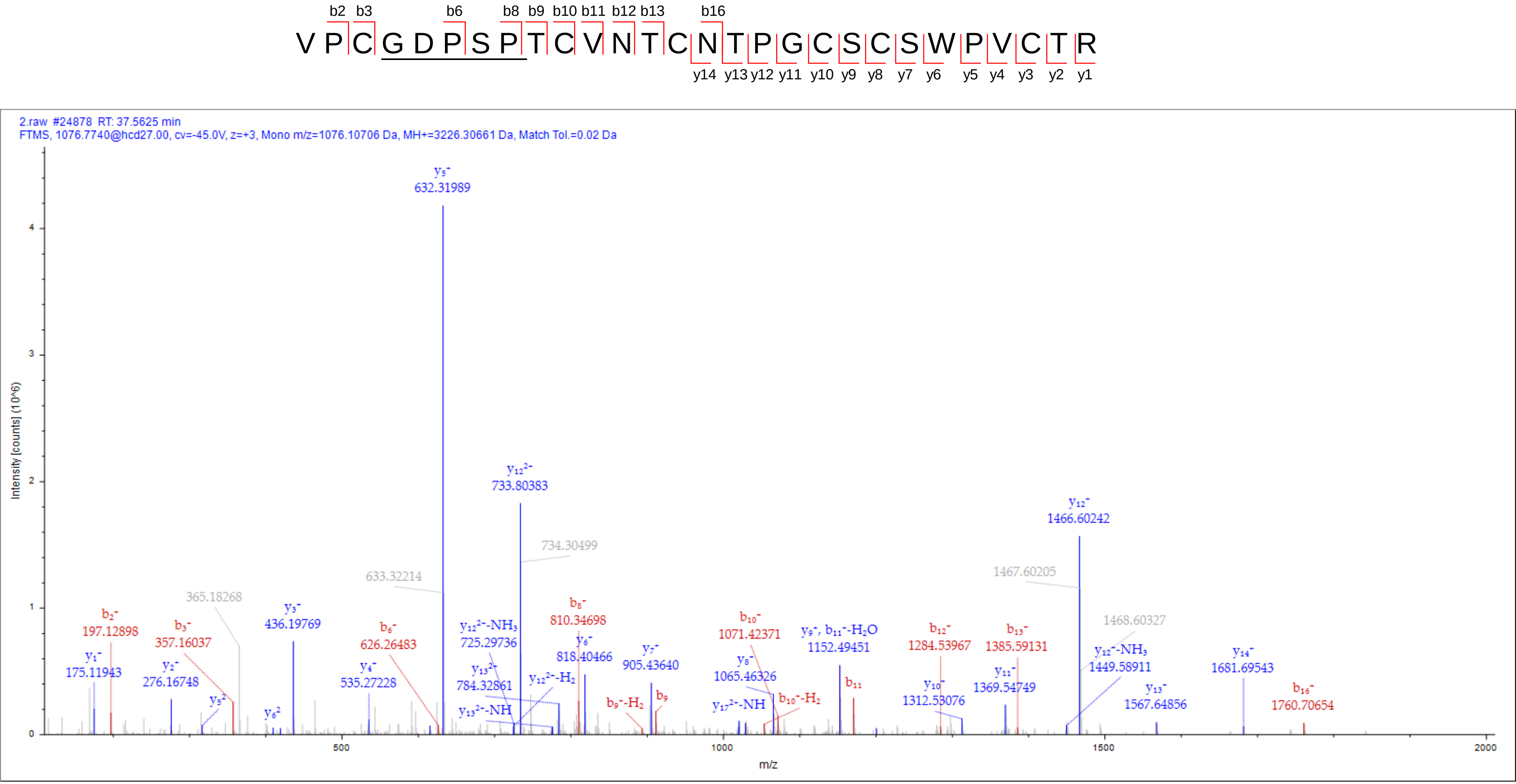

Viphi J_XGPVCADTCTXGTCYTAGCSCSWPVCTR

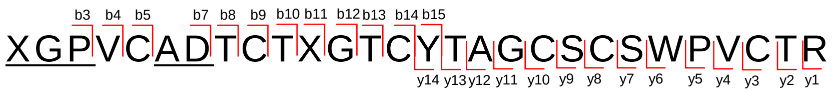

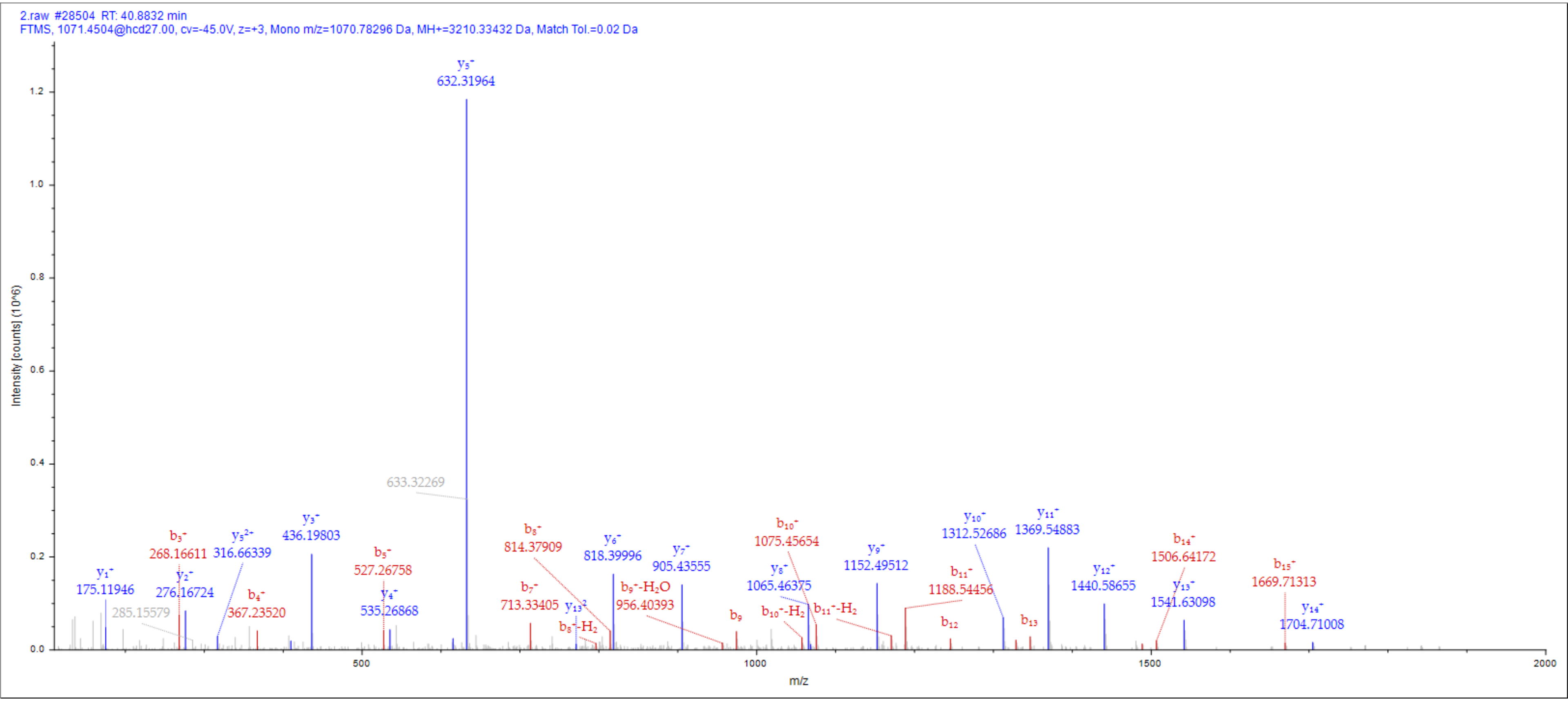

Viphi K_XGPVCGETCTXGTCYTAGCSCSWPVCTR

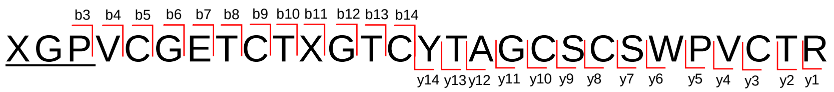

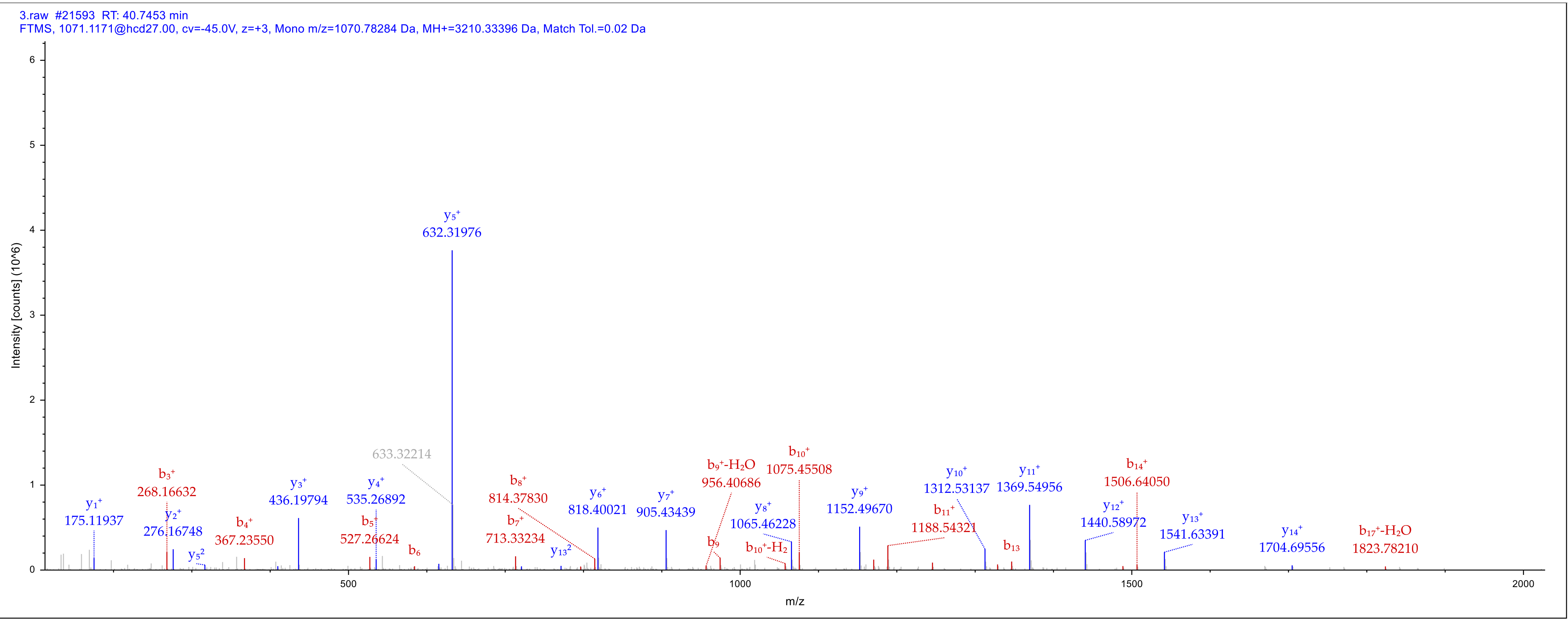

Viphi L_NGXPVCGETCVCYSSDPGCTCSWPVCTR

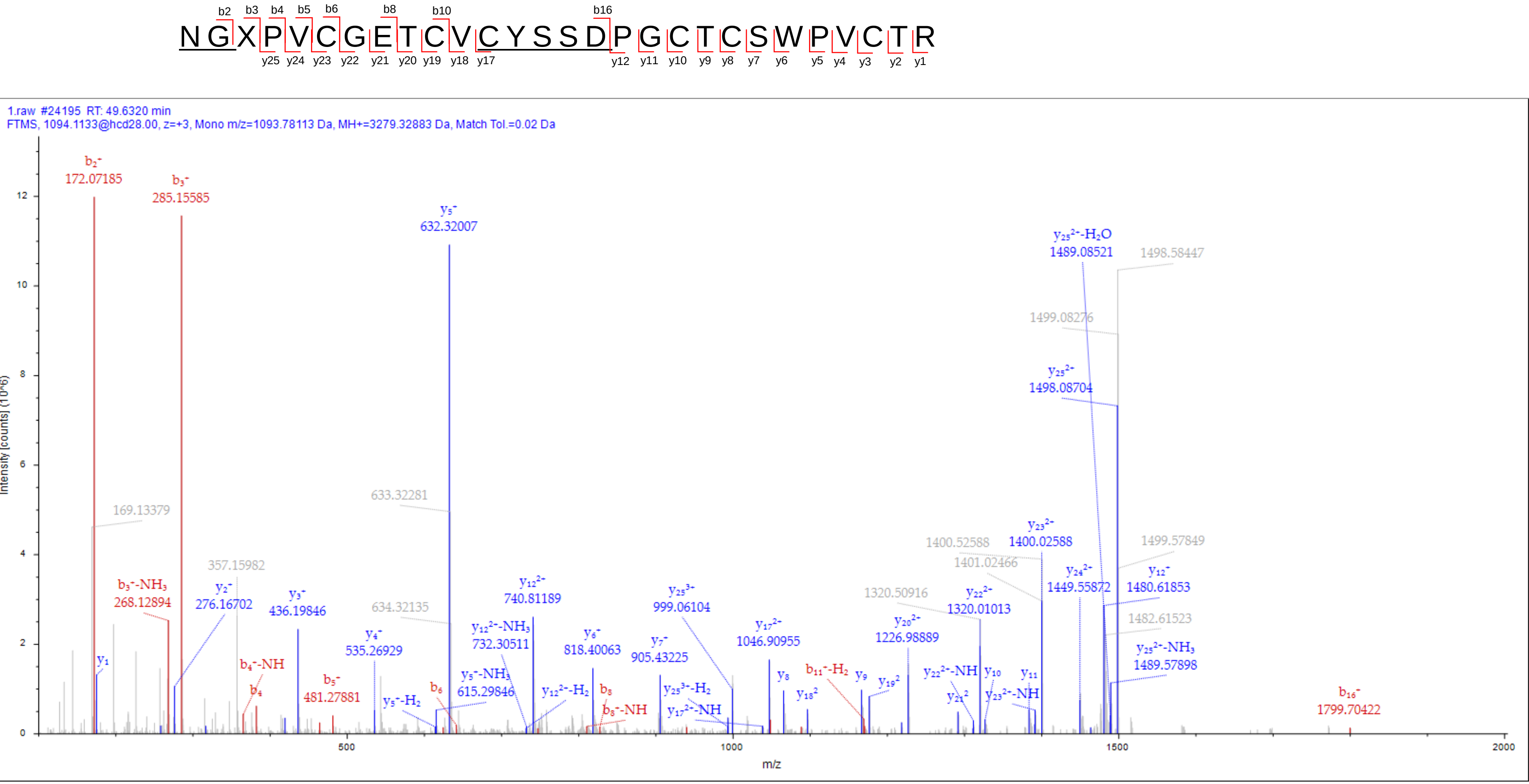

Viphi M_VPCGETCVAVGGTCNTPGCTCSWPVCTR

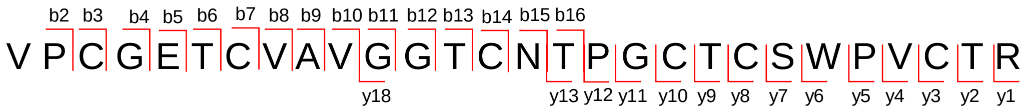

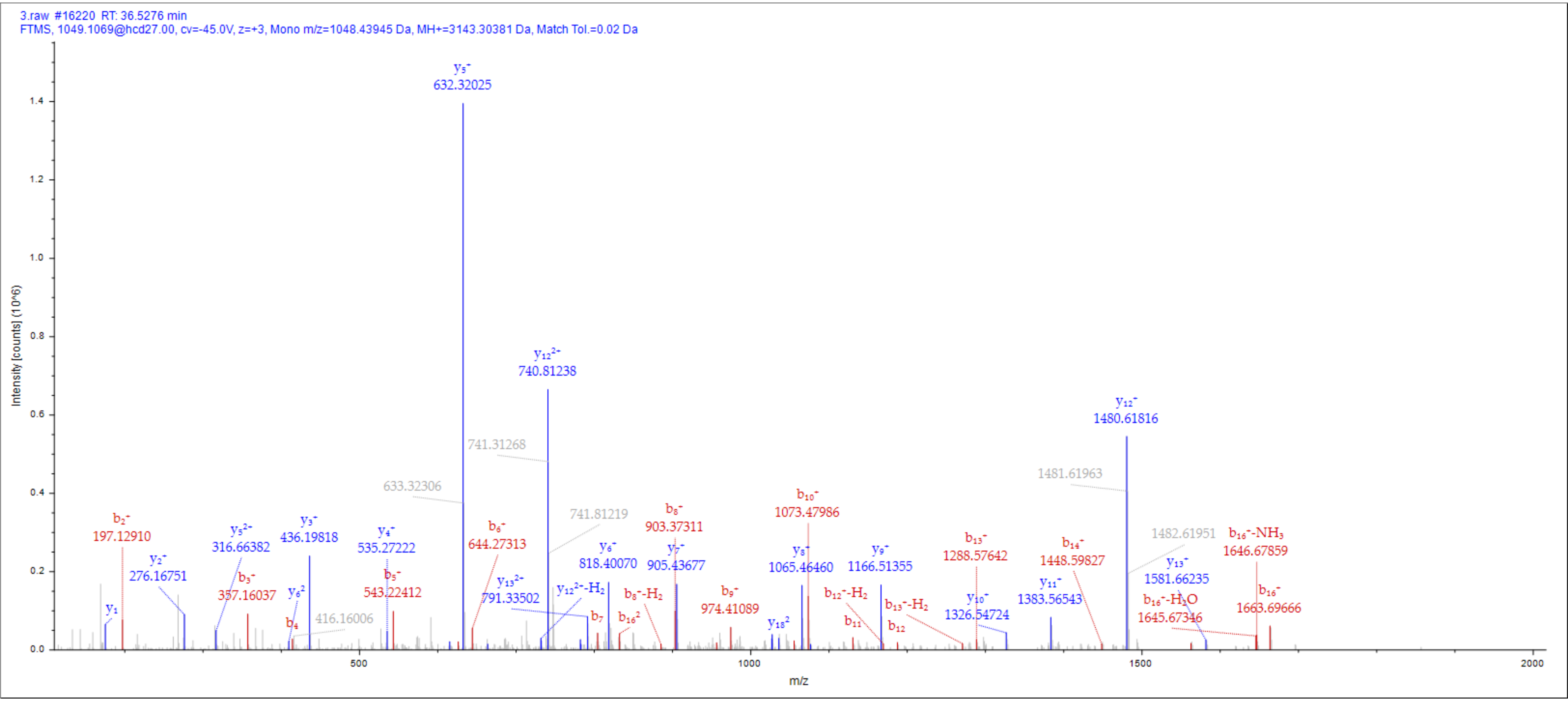

Viphi N_DGXPXCGETCVGGTCNTPGCSCSWPVCTR

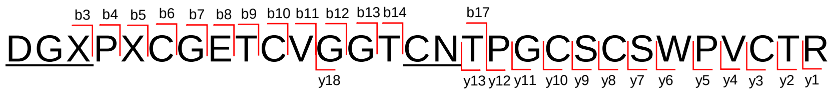

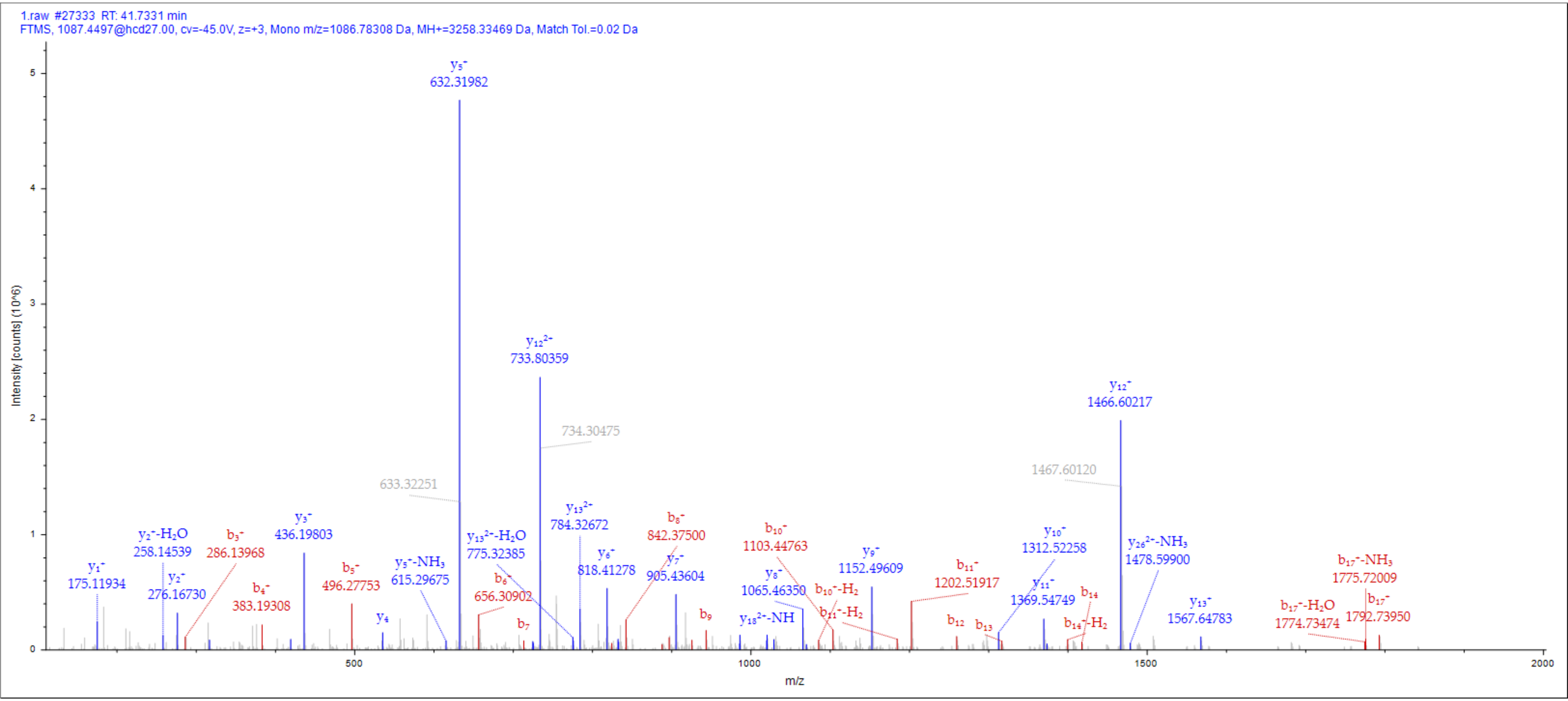

Viphi O_DGXPVCGETCVGGTCNTPGCSCSWPVCTR

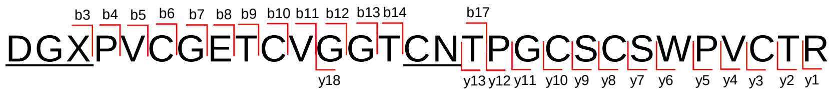

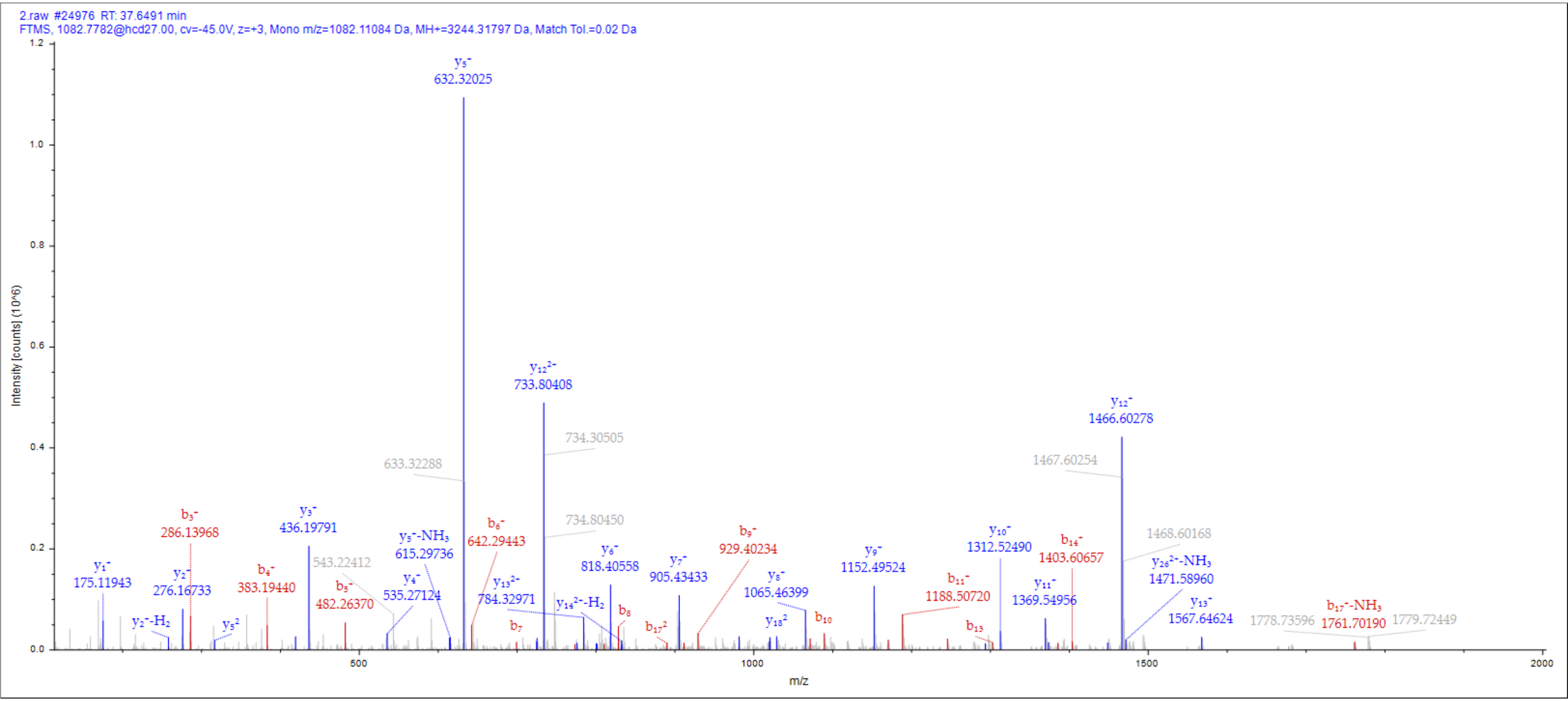

Viphi P_NGXPXCGETCVGGTCNTPGCVCSWPVCTR

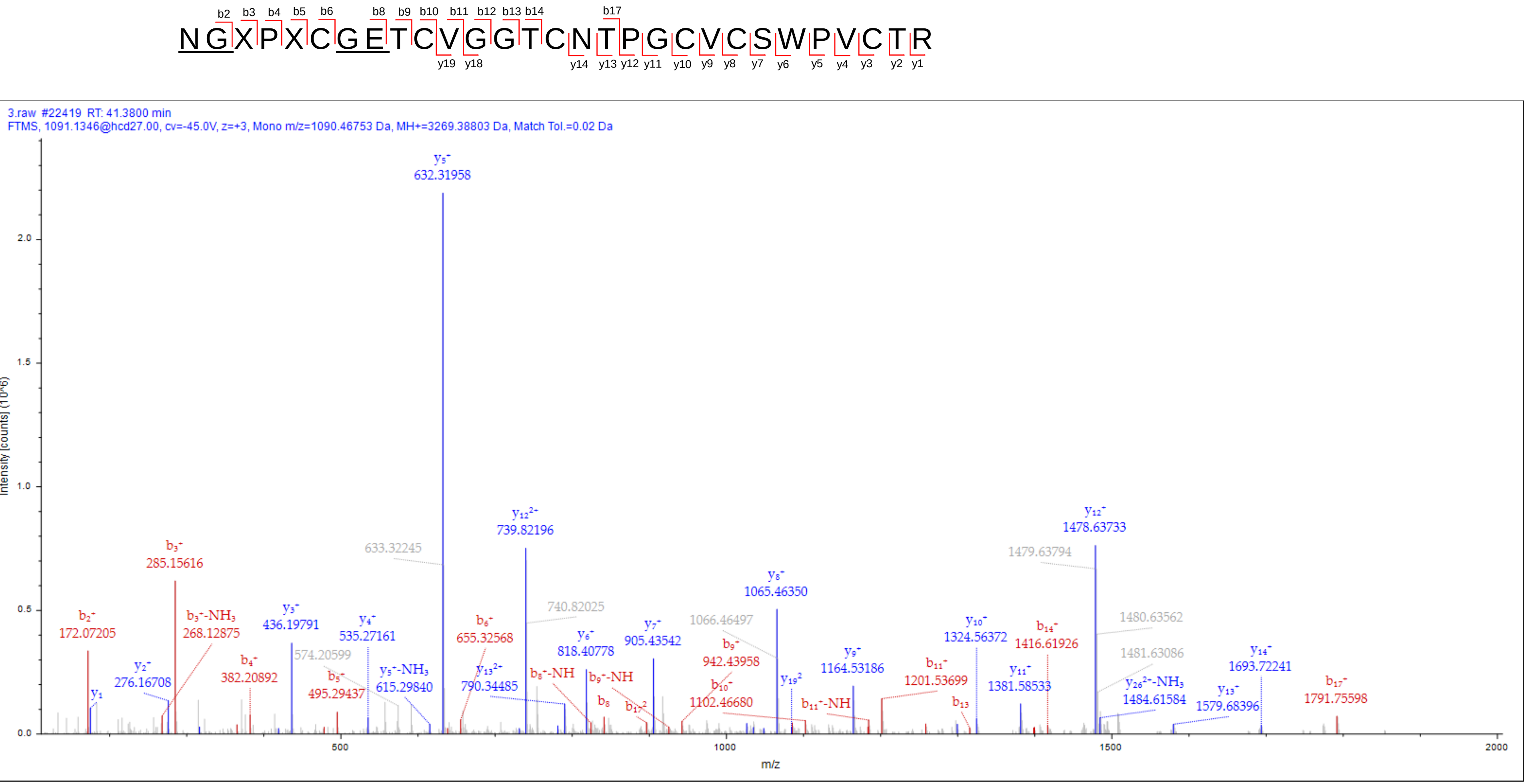

Viphi Q_DGXPVCGETCTXGTCYTAGCSCSWPVCTR

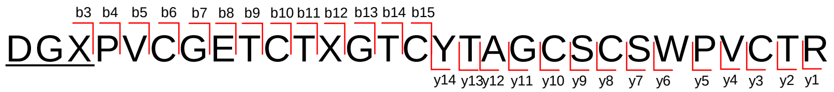

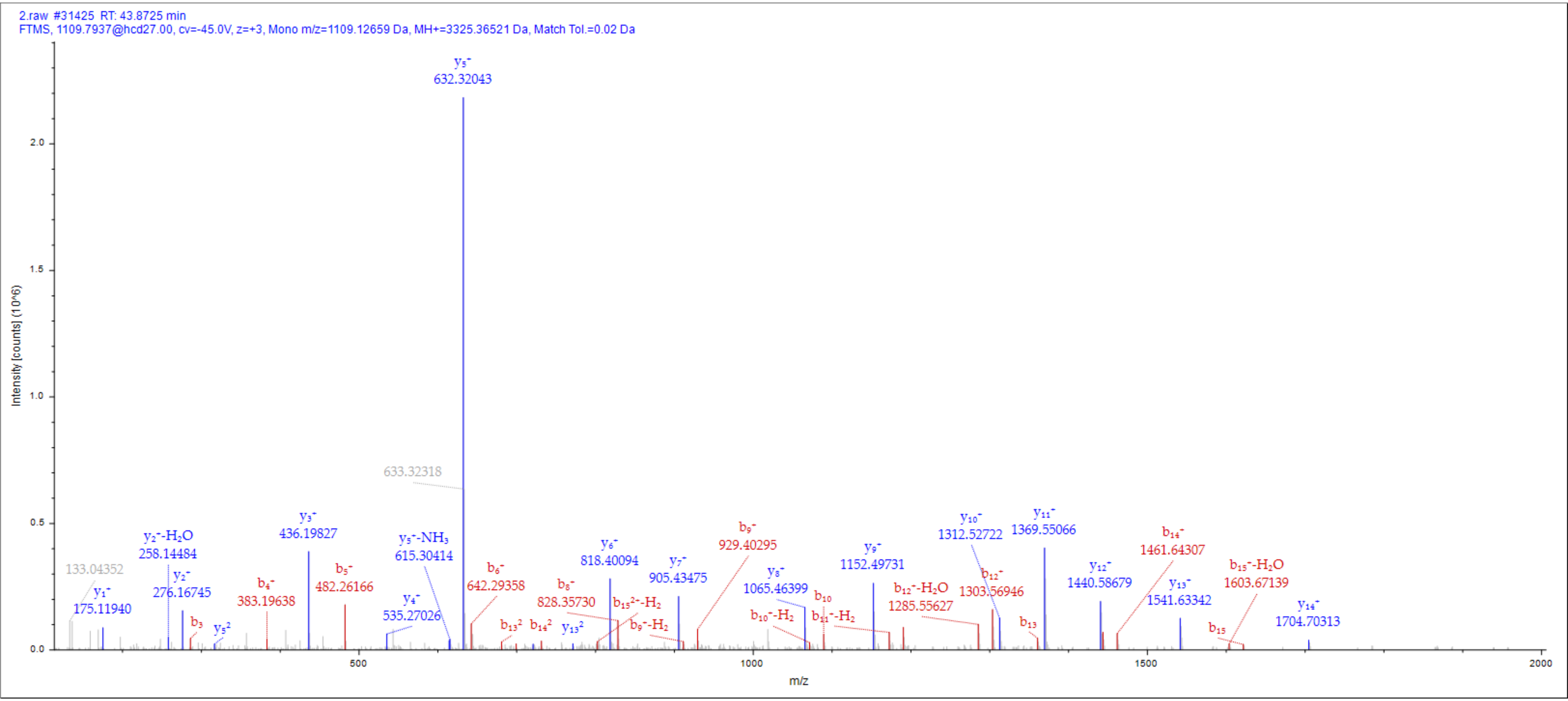

Viphi R_NGXPXCGETCVGGTCDTPGCTCSWPVCTR

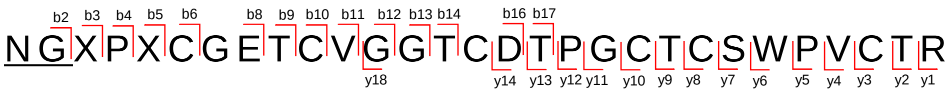

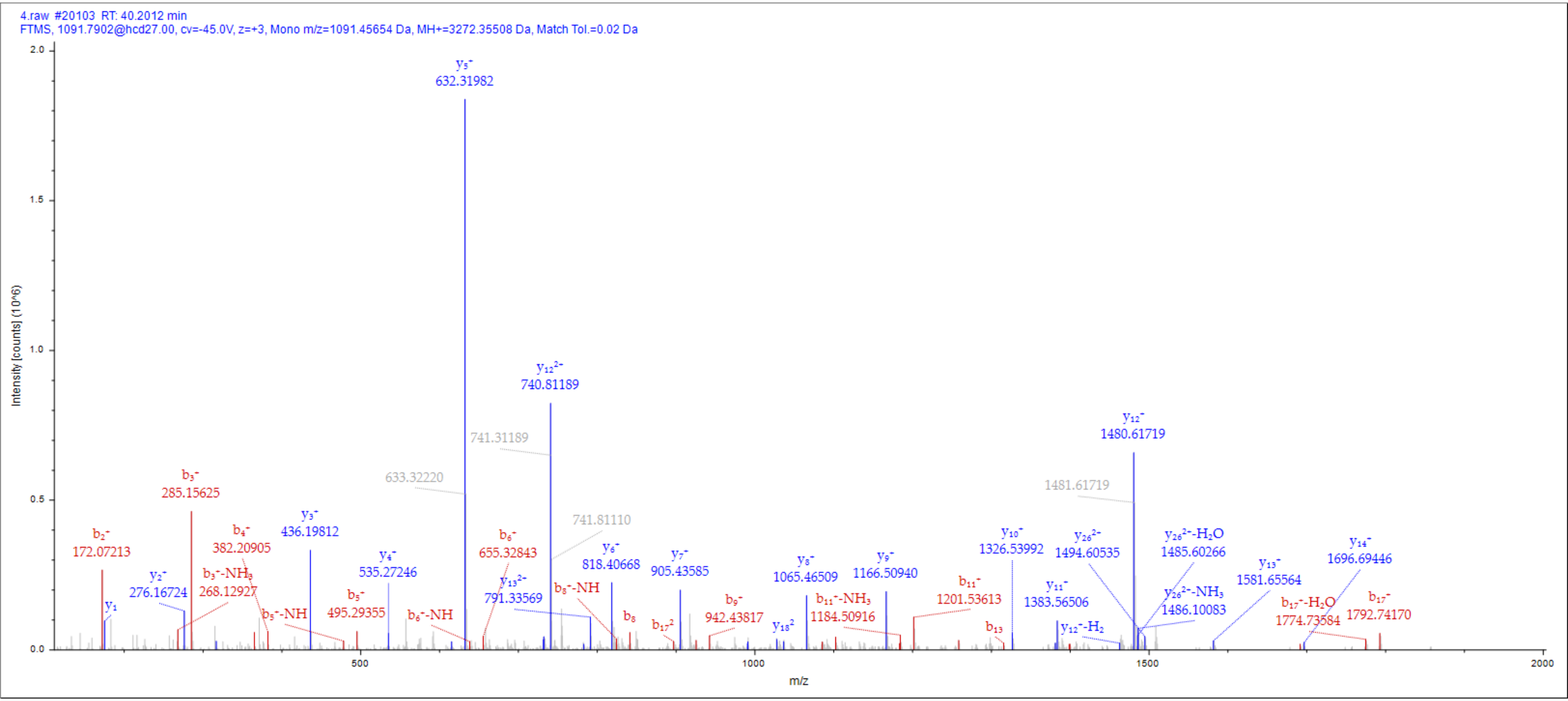

Viphi S_NGXPXCGETCVGDSDPTPGCTCXCPVCTR

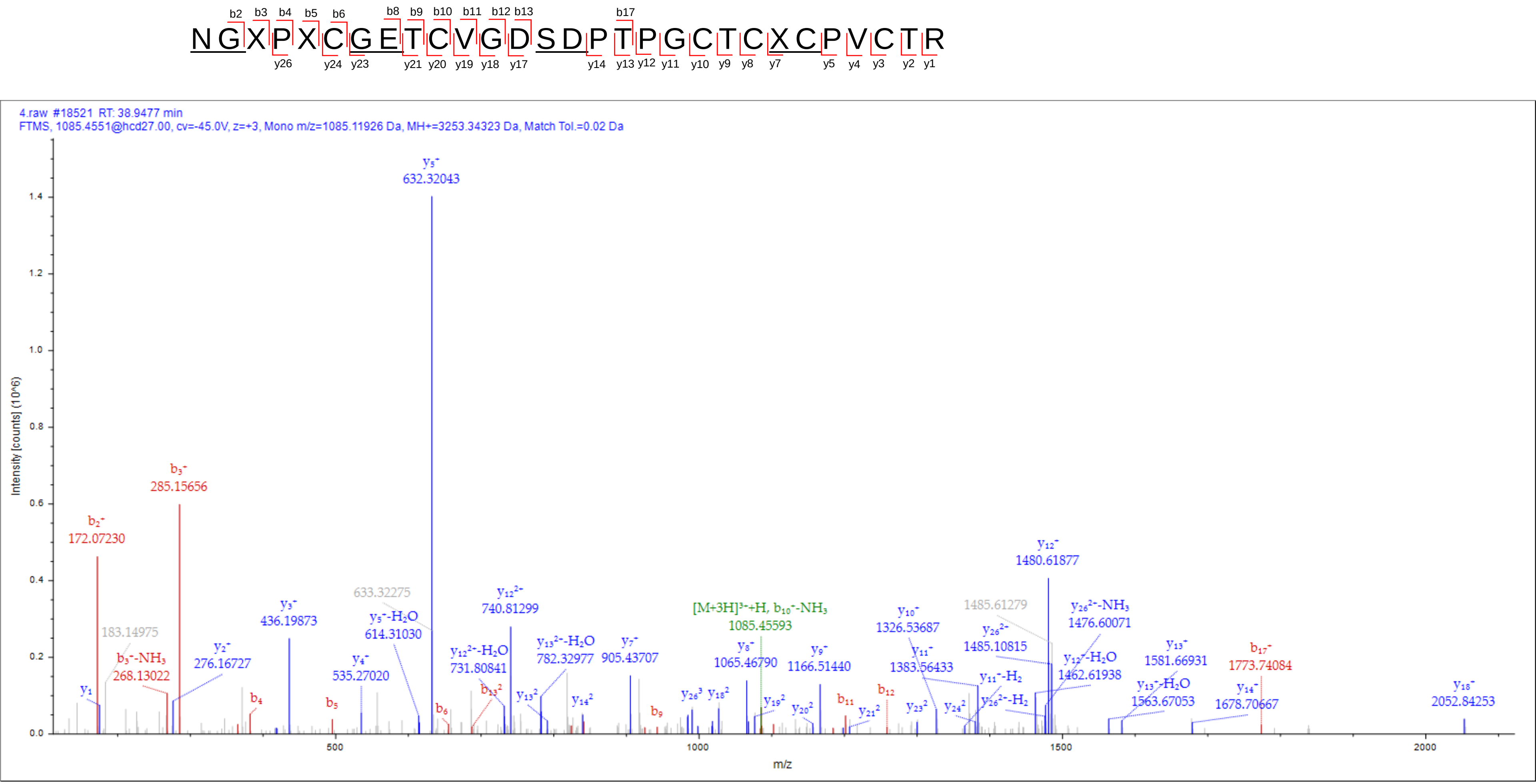

Viphi T_DGXPVCGETCVGGTCNTPGCACSWPVCTR

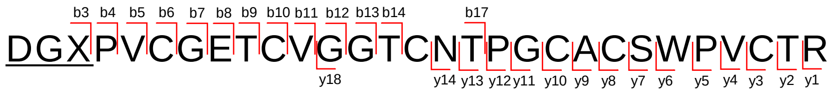

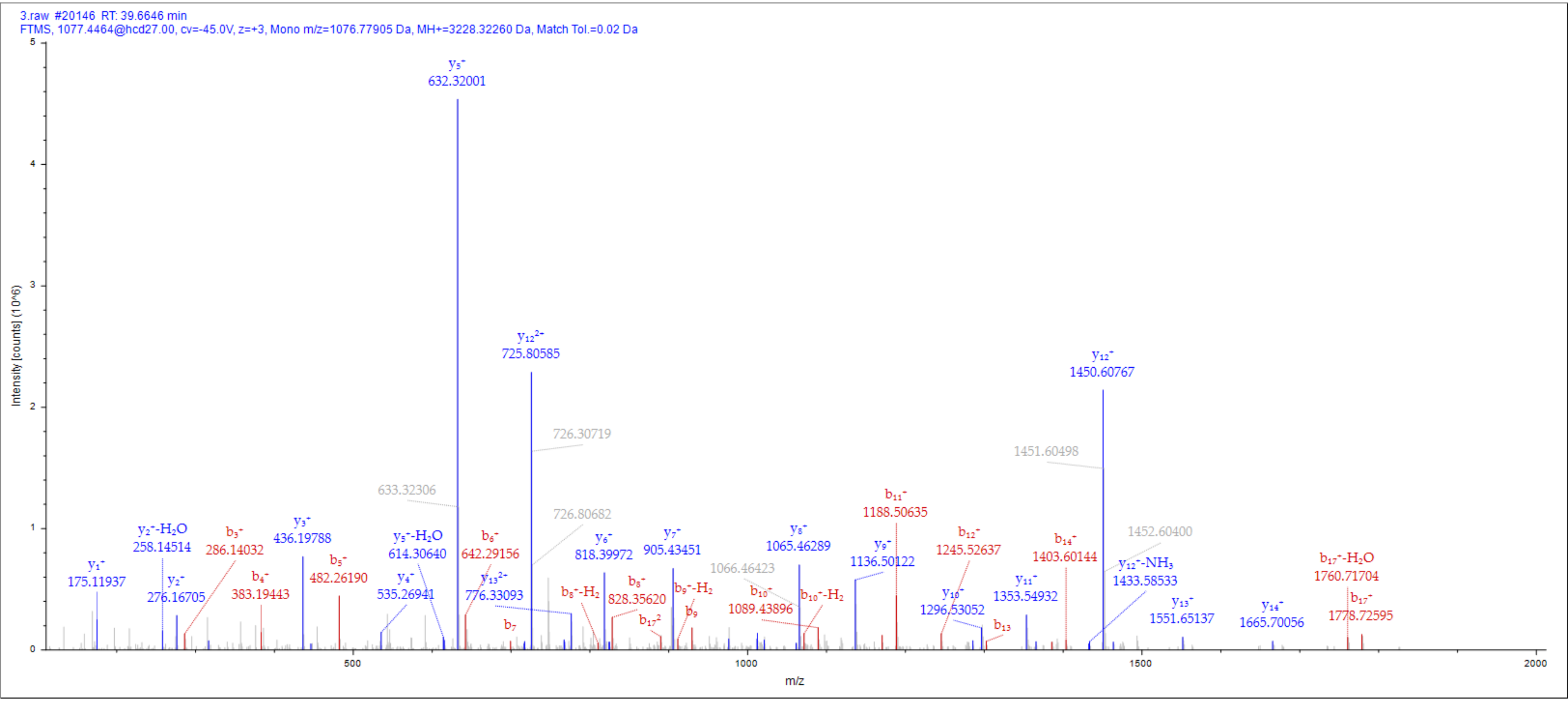

Viphi U_NGXPVCEGTCVGGTCNYGGCSCSWPVCTR

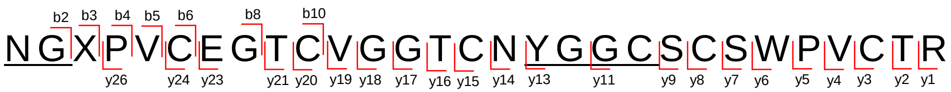

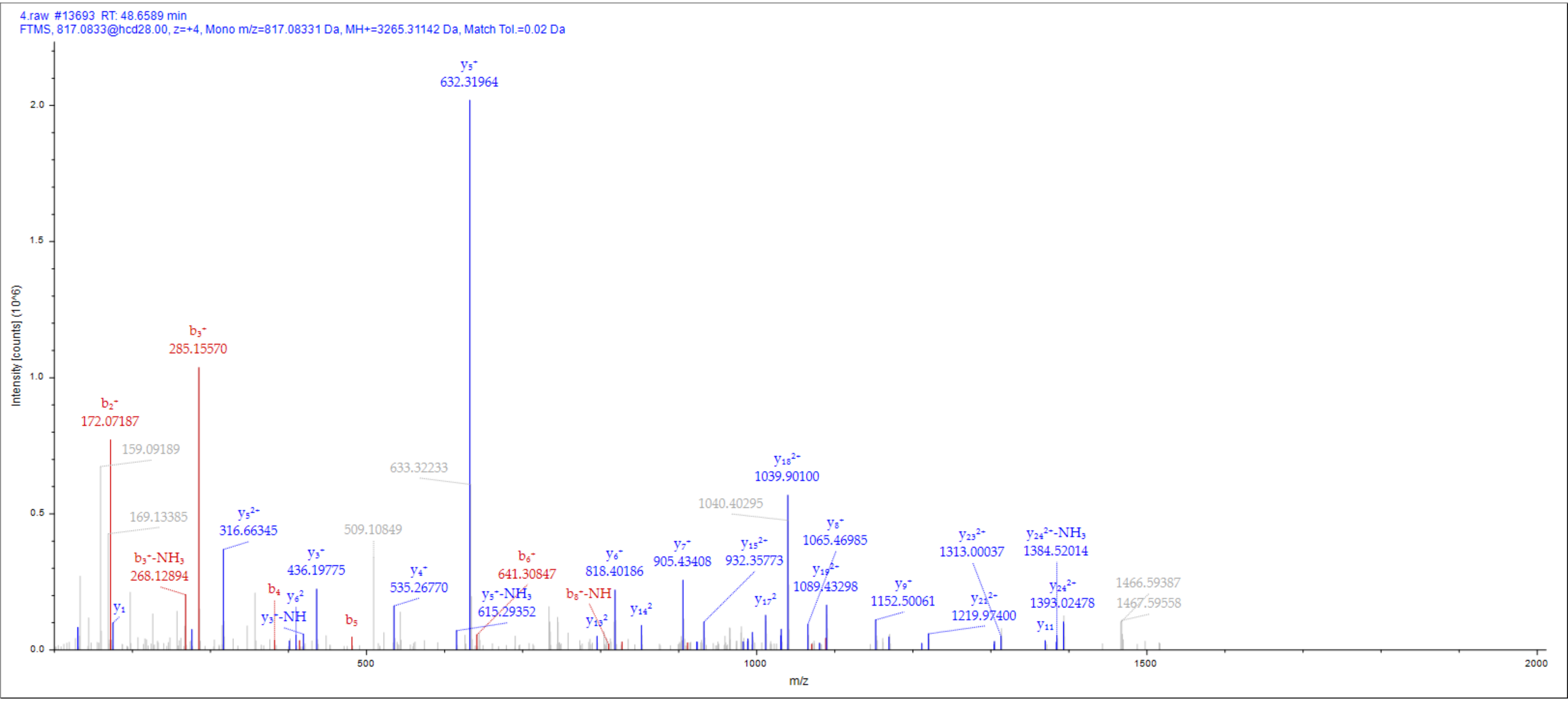

Viphi V_VPCGETCVGGAVCQSNTPGCTCSWPVCTR

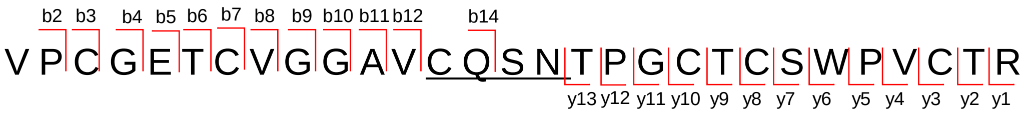

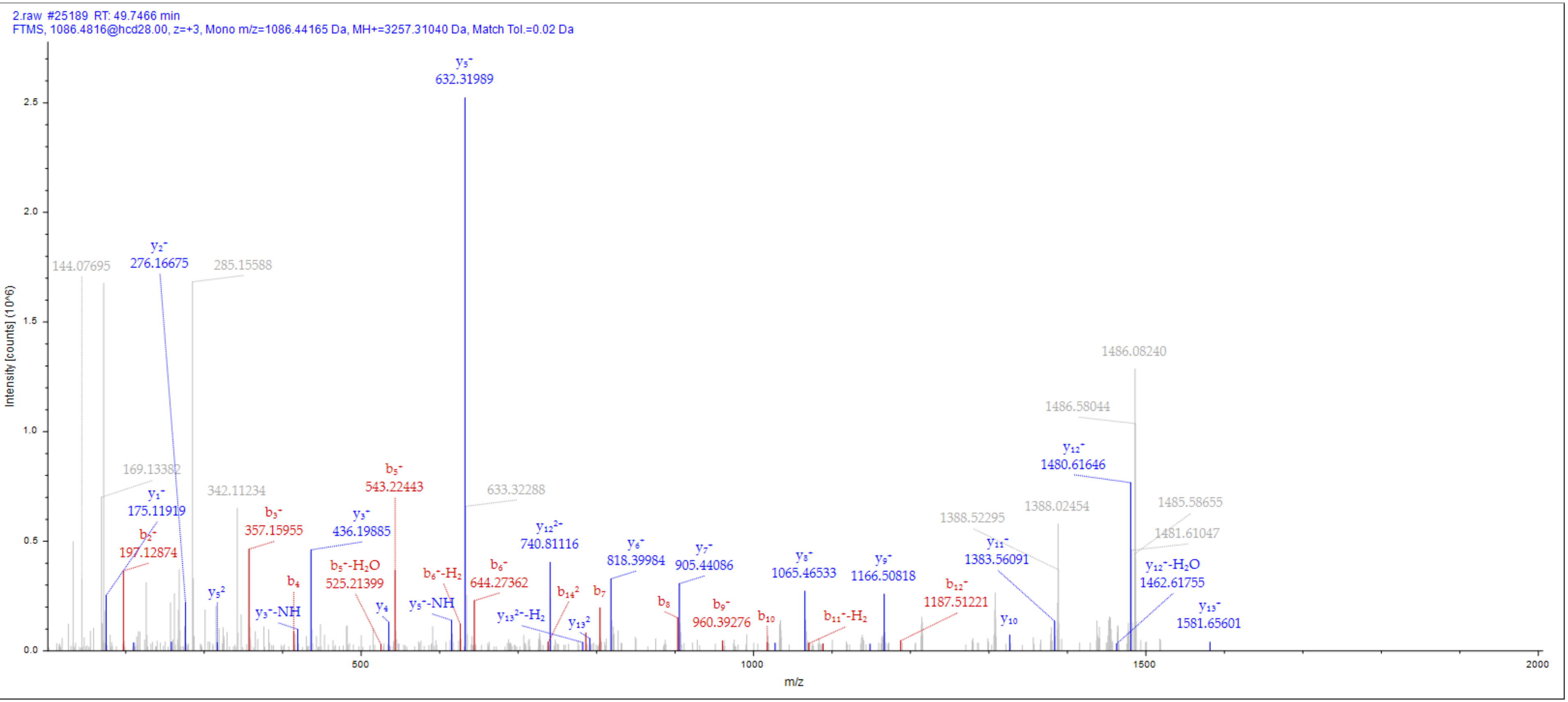

Viphi W_NGXPVCADTCVGGTCNTPGCACYNPVCTR

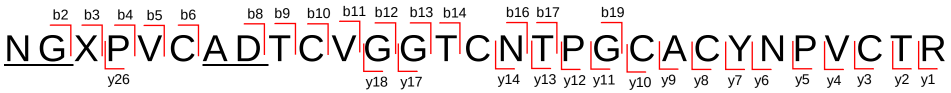

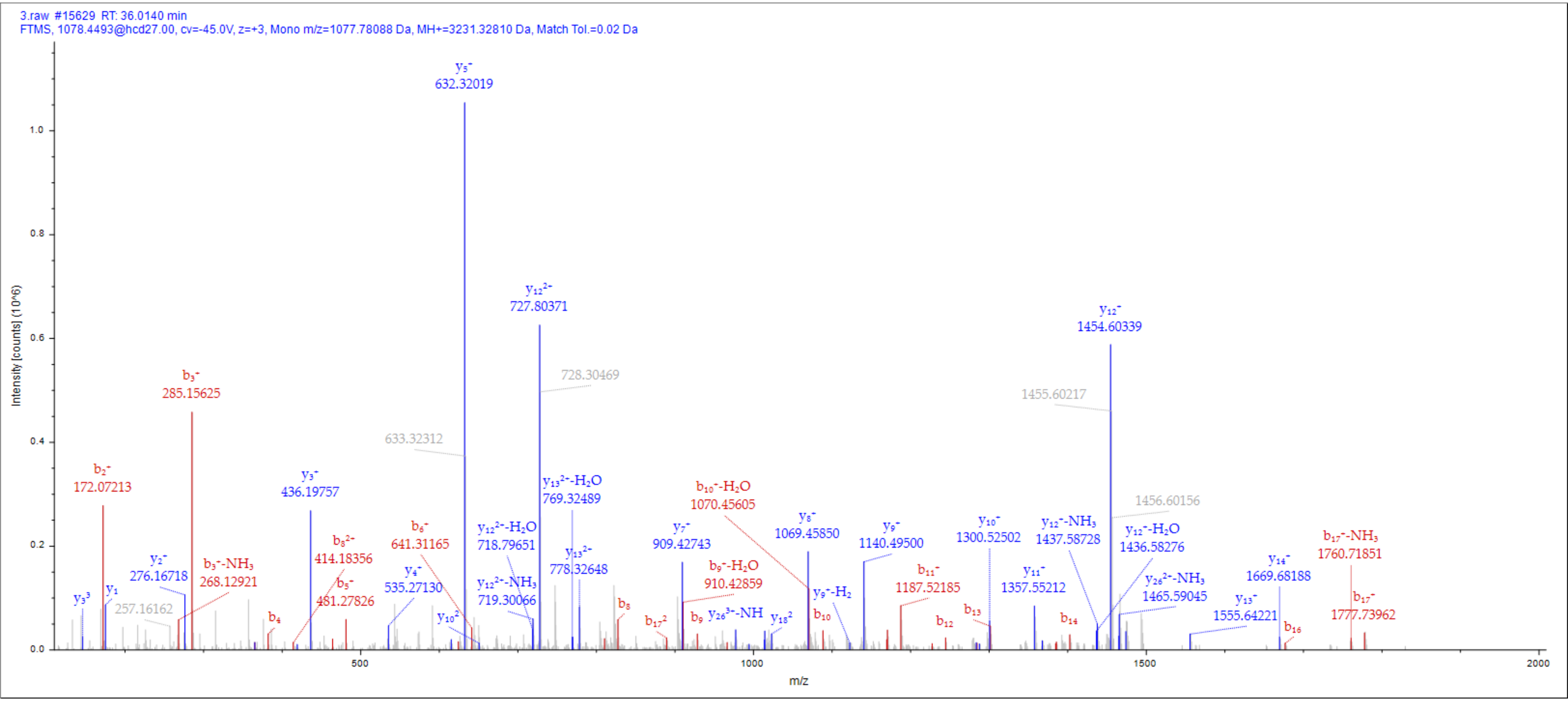

Viphi X_NGXPXCADTCVGGTCNTPGCSCSMAPVCTR

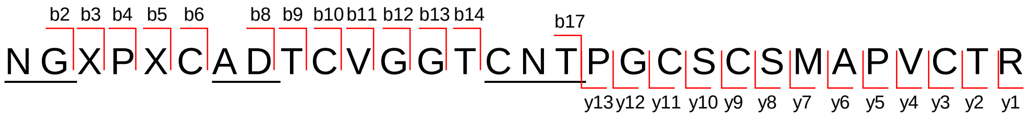

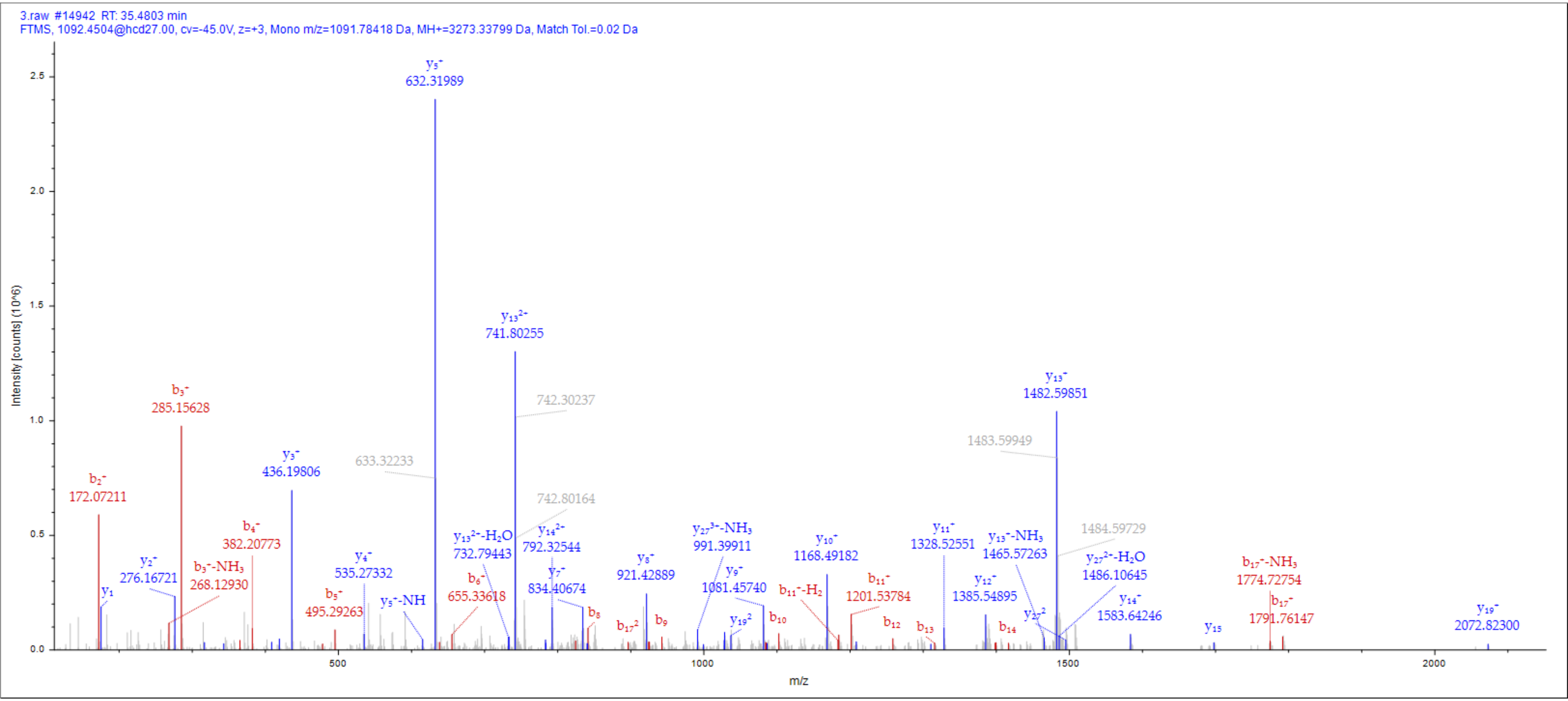

Viphi Y_VCYNGXTMCSSCVWXPCTVTAXVGCSCSDK

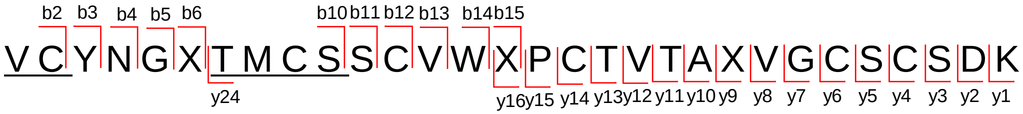

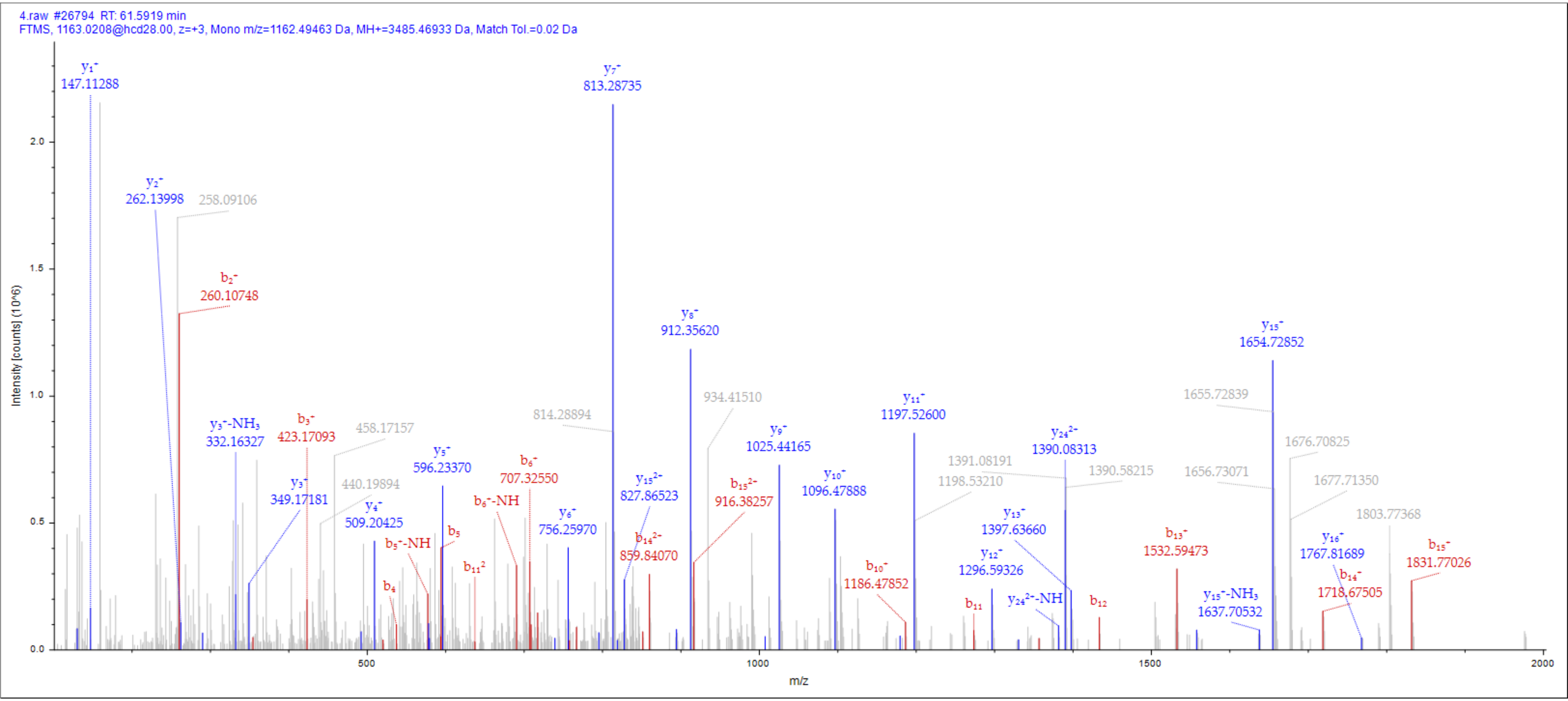

Viphi Z_NGXPXCEGTCVGGTCNTPGCSCSMAPVCTR

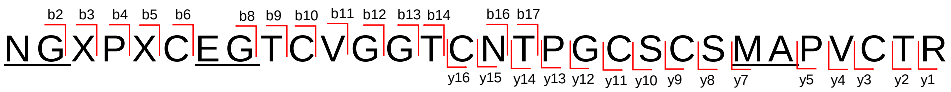

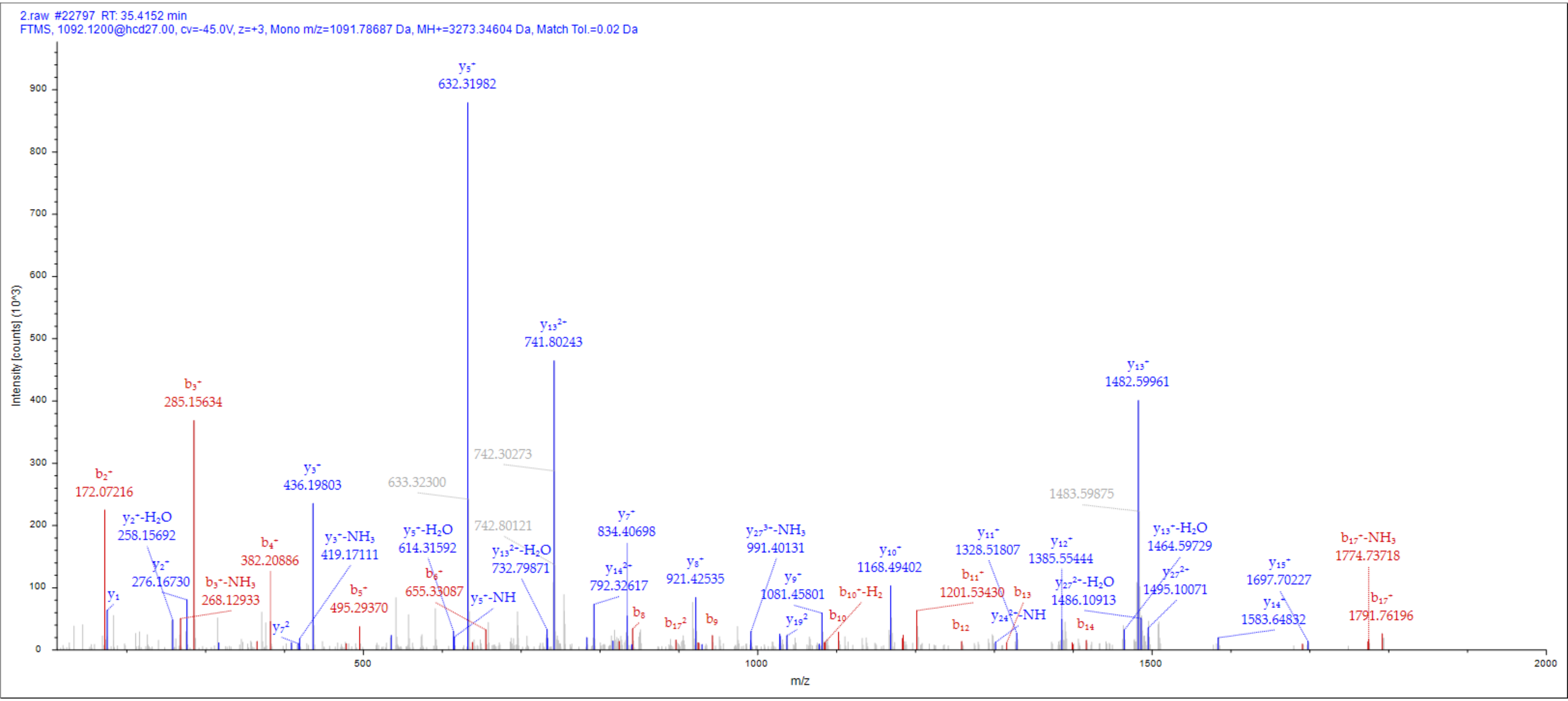

Supplement: Supplementary file 1 [file molecules-29-04344-s001.zip › Supplementary Figures.pdf]
